# Gene-based polygenic risk scores analysis of alcohol use disorder in African Americans

**DOI:** 10.1038/s41398-022-02029-2

**Published:** 2022-07-05

**Authors:** Dongbing Lai, Tae-Hwi Schwantes-An, Marco Abreu, Grace Chan, Victor Hesselbrock, Chella Kamarajan, Yunlong Liu, Jacquelyn L. Meyers, John I. Nurnberger, Martin H. Plawecki, Leah Wetherill, Marc Schuckit, Pengyue Zhang, Howard J. Edenberg, Bernice Porjesz, Arpana Agrawal, Tatiana Foroud

**Affiliations:** 1 Department of Medical and Molecular Genetics, Indiana University School of Medicine, Indianapolis, IN USA; 2 Department of Psychiatry, University of Connecticut School of Medicine, Farmington, CT USA; 3 Department of Psychiatry, University of Iowa, Carver College of Medicine, Iowa City, IA USA; 4 Henri Begleiter Neurodynamics Lab, Department of Psychiatry, State University of New York, Downstate Medical Center, Brooklyn, NY USA; 5 Department of Psychiatry, Indiana University School of Medicine, Indianapolis, IN USA; 6 Department of Psychiatry, University of California, San Diego Medical School, San Diego, CA USA; 7 Department of Biostatistics and Health Data Science, Indiana University School of Medicine, Indianapolis, IN USA; 8 Department of Biochemistry and Molecular Biology, Indiana University School of Medicine, Indianapolis, IN USA; 9 Department of Psychiatry, Washington University School of Medicine, St. Louis, MO USA

**Keywords:** Addiction, Personalized medicine

## Abstract

Genome-wide association studies (GWAS) in admixed populations such as African Americans (AA) have limited sample sizes, resulting in poor performance of polygenic risk scores (PRS). Based on the observations that many disease-causing genes are shared between AA and European ancestry (EA) populations, and some disease-causing variants are located within the boundaries of these genes, we proposed a novel gene-based PRS framework (PRS_gene_) by using variants located within disease-associated genes. Using the AA GWAS of alcohol use disorder (AUD) from the Million Veteran Program and the EA GWAS of problematic alcohol use as the discovery GWAS, we identified 858 variants from 410 genes that were AUD-related in both AA and EA. PRS_gene_ calculated using these variants were significantly associated with AUD in three AA target datasets (*P*-values ranged from 7.61E−05 to 6.27E−03; Betas ranged from 0.15 to 0.21) and outperformed PRS calculated using all variants (*P*-values ranged from 7.28E−03 to 0.16; Betas ranged from 0.06 to 0.18). PRS_gene_ were also associated with AUD in an EA target dataset (*P*-value = 0.02, Beta = 0.11). In AA, individuals in the highest PRS_gene_ decile had an odds ratio of 1.76 (95% CI: 1.32–2.34) to develop AUD compared to those in the lowest decile. The 410 genes were enriched in 54 Gene Ontology biological processes, including ethanol oxidation and processes involving the synaptic system, which are known to be AUD-related. In addition, 26 genes were targets of drugs used to treat AUD or other diseases that might be considered for repurposing to treat AUD. Our study demonstrated that the gene-based PRS had improved performance in evaluating AUD risk in AA and provided new insight into AUD genetics.

## Introduction

Alcohol use disorder (AUD) is one of the most common public health problems [1] and both genetic and environmental factors contribute to risk. Estimates of the heritability of AUD range from 40% to 60% [2–4]. Recently, several large-scale genome-wide association studies (GWAS) of AUD-related phenotypes have reported many genetic variants associated with AUD [5–7]. These GWAS reiterated the highly polygenic underpinnings of AUD and related phenotypes where many variants contribute small effects on AUD. Consequently, polygenic risk scores (PRS) have proven to be a strong approach for assessing AUD genetic liability beyond the genome-wide significant variants [5, 7]. For instance, in our previous study of an European ancestry cohort [8], individuals comprising the top PRS decile were almost twice as likely to meet the criteria for AUD compared to all others, an estimate comparable to those published for the first-degree family history of AUD in national surveys [9, 10]. However, PRS analysis of AUD in admixed populations, such as African Americans (AA), suffer from poor performance due to the much smaller sample sizes of the discovery GWAS [5].

The performance of PRS relies on well-powered discovery GWAS to accurately select the disease-associated variants and estimate their effect sizes, and well-matched target datasets. For admixed populations, the sample sizes of the discovery GWAS comparable to European ancestry (EA) populations (hundreds of thousands to >1 million) require extensive and strategic data collection. Studies have shown that many disease-causing genes are shared among different populations [11–14]. Therefore, large-scale EA GWAS summary statistics can be leveraged to improve the performance of PRS in non-EA populations by increasing the overall discovery GWAS sample size. However, disease-associated variants may have different allele frequencies and effect sizes in different populations, and linkage disequilibrium (LD) patterns are also different [12, 15–18], i.e., the target datasets are not matched to the discovery GWAS. Furthermore, for admixed populations such as AA, the proportions of African ancestry range from close to 0 to almost 100% and are differently distributed across the genome, making AA an extremely heterogeneous population. Therefore, different AA target datasets may also have different LD patterns and allele frequencies, and PRS results from one study cannot be generalized to other studies. Methods aimed to address these challenges have been proposed, but their performance remains far from ideal [19–23].

The majority of variants in the genome are likely not related to a particular condition and including them in PRS calculations will reduce the performance by introducing noises. Ideally, only variants that act on disease-causing genes should be used in calculating PRS. However, most of these variants and genes remain to be discovered. If a variant located in a gene is nominally associated (e.g., *P*-values < 0.05) with a disease in both EA and non-EA populations and has the same direction of effect, then it is more likely to be a shared disease-causing variant and that gene is likely to be a shared disease-causing gene across populations. Therefore, using these variants to calculate PRS is expected to improve the performance by excluding many variants in the genome that are unlikely to be related to a disease, thereby increasing the signal-to-noise ratio. Moreover, since these disease-causing variants are shared among different populations, the discovery GWAS and target datasets do not have to be well-matched and the large-scale EA GWAS can be used to increase the overall discovery GWAS sample size. Based on these observations, we propose a novel gene-based PRS framework aimed at enhancing the performance of PRS in admixed populations. We first used an AA GWAS and an EA GWAS to identify genes that were associated with AUD in both populations, then used variants located within these genes’ boundaries to calculate PRS (PRS_gene_). We compared the performance of PRS_gene_ with PRS calculated using variants located in intergenic regions (PRS_intergenic_) and all available variants (PRS_all_). Furthermore, for genes included in gene-based PRS calculations, we performed gene enrichment analysis using Gene Ontology (http://geneontology.org/) to test whether they were enriched in AUD or other biological processes that could provide novel insight into AUD mechanism. In addition, we tested in which tissues these genes were enriched. We also searched a publically available drug target database [24] to evaluate whether these genes were potential drug targets for AUD treatment, or drug targets for the treatment of other diseases but may be repurposed to treat AUD.

## Methods

### Discovery GWAS

The discovery GWAS were from the meta-GWAS of problematic alcohol use in EA cohorts (EA-PAU) (*N* = 435,563) [7] and the AA GWAS of AUD from the Million Veteran Program (AA-AUD) (*N* = 56,648) [5], the largest GWAS of AUD-related phenotypes to date in EA and AA populations, respectively. The EA-PAU was a meta-analysis of problematic alcohol use [7] comprised of the AUD GWAS of the Million Veteran Program Phase I [5] and Phase II data, the alcohol dependence GWAS from the Psychiatric Genomics Consortium [25], and the GWAS of scores from the problem subscale of the Alcohol Use Disorder Identification Test (AUDIT items 4 to 10) in the UK Biobank [6]. Across both discovery GWAS, A/T, or C/G variants were excluded to avoid strand ambiguity. As we were focusing on AUD-associated variants shared between EA and AA, only variants having the same directions of effects in both EA-PAU and AA-AUD were included. Both GWAS summary statistics were downloaded from the database of genotypes and phenotypes (dbGaP: phs001672.v3.p1, https://www.ncbi.nlm.nih.gov/projects/gap/cgi-bin/study.cgi?study_id=phs001672.v3.p1).

### Target datasets

AA target datasets were drawn from 3 sources: the Collaborative Study on the Genetics of Alcoholism (COGA: *N* = 3375) [26], Study of Addiction: Genetics and Environment (SAGE: *N* = 930) [27], and YalePenn (*N* = 2010) [28]. COGA is a family cohort, in which alcohol-dependent probands and their family members from inpatient and outpatient alcohol dependence treatment facilities in seven sites were invited to participate. COGA also recruited community comparison families from a variety of sources in the same areas [26, 29]. The study was approved by Institutional review boards from all sites. Every participant provided informed consent. The Semi-Structured Assessment for the Genetics of Alcoholism (SSAGA) was administered to individuals 18 or over and the child version of the SSAGA was used for those younger than 18 [30, 31]. SAGE (phs000092.v1.p1, https://www.ncbi.nlm.nih.gov/projects/gap/cgi-bin/study.cgi?study_id=phs000092.v1.p1) and YalePenn (phs000425.v1.p1, https://www.ncbi.nlm.nih.gov/projects/gap/cgi-bin/study.cgi?study_id=phs000425.v1.p1) were downloaded from dbGaP. Since COGA had more phenotypic information, if a sample in the COGA dataset was also in SAGE and/or YalePenn, it was only analyzed as part of the COGA data. SAGE and YalePenn were mixes of related and unrelated individuals, although most were unrelated. Only AA samples from COGA, SAGE, and YalePenn were used. Across all three datasets, AUD was defined as meeting lifetime criteria for DSM-IV alcohol dependence [32] or DSM-5 alcohol use disorder [33]. All other individuals were considered as controls.

The gene-based PRS_gene_ were calculated using variants located in AUD genes implicated in both AA and EA (i.e., *P*-values < 0.05 in both populations), consequently, they should be applicable to both populations. To test this proposition, an EA target dataset was also tested. As some EA samples of COGA and SAGE data were part of EA-PAU, they were not included as the target EA datasets, instead, EA individuals were drawn from the Indiana Biobank (https://indianabiobank.org/). The Indiana Biobank is a state-wide collaboration that provides centralized processing and storage of specimens that are linked to participants’ electronic medical information via Regenstrief Institute at Indiana University. All Indiana Biobank individuals included in this study were unrelated. AUD in Indiana Biobank was diagnosed based on ICD9 (303 and 305.0) and ICD10 (F10) codes. Individuals not diagnosed as AUD and without AUD-associated conditions such as alcohol-associated pancreatitis were defined as controls.

### Genotype data processing and imputation

Detailed information about COGA, SAGE, and YalePenn data processing has been reported previously [34–36]. Briefly, all data were combined and a common set of high quality (minor allele frequency (MAF) > 10%, missing rate <2%, Hardy-Weinberg Equilibrium (HWE) *P*-values > 0.001) and independent (defined as *R*^2^ < 0.5) variants (*N* = 24,135) was used to identify duplicate samples among different target datasets and confirm the reported family structures using PLINK [37, 38]; family structures were updated as needed. The same set of common variants was also used to estimate the principal components (PCs) of population stratification using Eigenstrat [39] with 1000 Genomes data (Phase 3, version 5, NCBI GRCh37) as the reference panel. These PCs were also used to determine AA samples (first PC between -0.0043 and 0.0115 and second PC between -0.0035 and 0.0059). Due to the different arrays used, each target dataset was imputed separately to 1000 Genomes by using SHAPEIT2 [40] followed by Minimac3 [41]. Before imputation, variants with A/T or C/G alleles, missing rates >5%, MAF < 3%, and HWE *P*-values < 0.0001 were excluded. Imputed variants with *R*^2^ ≥ 0.30 and MAF ≥ 1% were included in all analyses. Indiana Biobank samples were genotyped using Illumina Infinium Global Screening Array (GSA, Illumina, San Diego, CA) by Regeneron (Tarrytown, NY). Variants with missing rate >5%, MAF < 1%, HWE *P*-value < 1E−10 among cases and 1E−6 in controls were excluded as reported previously [42]. Population stratification was then estimated using the SNPRelate package [43] from Bioconductor [44]. Indiana Biobank data were also imputed to 1000 Genomes using the Michigan Imputation Server (https://imputationserver.sph.umich.edu/index.html#!pages/home) [41]. Imputed variants with *R*^2^ < 0.30 and MAF < 1% were excluded.

### PRS calculation

We used PRS-CSx, a recently developed method designed for cross-ethnic polygenic prediction that showed better performance than other methods in simulation studies and real data analysis [23]. The posterior effect size of each variant was estimated via a Bayesian regression framework using continuous shrinkage priors. African and European samples from the 1000 Genomes Project were used as the LD reference panels. PRS-CSx can estimate posterior effect sizes of AA only, EA only, and meta-analysis of EA-PAU and AA-AUD. The authors of PRS-CSx recommend using estimated AA- and EA-only posterior effect sizes, then testing different linear combinations of them with different weights in a validating dataset, and choosing the one with the best performance for testing in independent datasets [23]. If the validation dataset and independent datasets are similar, e.g., having similar LD patterns and allele frequencies, this method will have more power. However, if they are different, then the weights estimated from the validating dataset will be biased toward that dataset and different from the independent datasets, resulting in loss of power. As we noted earlier, AA is a very heterogeneous population. The three AA target datasets in this study were recruited under different ascertainment strategies and in different regions, therefore, meta-analyzed posterior effect sizes were used in this study. In addition, since we only focused on AUD-associated variants implicated in both AA and EA, meta-analysis posterior effect sizes should provide more accurate estimates for those variants. We first selected variants that had *P*-values < 0.05 in both EA-PAU and AA-AUD (i.e., at least showing marginal associations) and had the same directions of effects (referred to as concordant variants). For our gene-based PRS (PRS_gene_), only concordant variants located within gene boundaries (defined as within the region containing the gene plus 1 kb upstream of the transcription start site and 1 kb downstream of the transcription end site; annotated using ANNOVAR [45] based on NCBI RefSeq GRCh37) were used. To test whether using any concordant variants regardless of location would do as well, we calculated PRS using concordant variants located outside gene boundaries (referred to as PRS_intergenic_). We also tested whether further extending gene boundaries used to calculate PRS_gene_ improved results by setting different window sizes: 10 kb, 25 kb, 50 kb, 100 kb, 250 kb, 500 kb, 1 Mb, 50 Mb, and 100 Mb. Lastly, we also used all variants across the entire genome to calculate PRS (PRS_all_) for comparison purposes. For all AA target datasets, PRS_gene,_ PRS_intergenic_, and PRS_all_ were calculated using exactly the same sets of variants, respectively, thus they were directly comparable and can be combined. PLINK [37, 38] was used to calculate PRS using the posterior effect sizes estimated by PRS-CSx and imputation dosages. All PRS were standardized as mean = 0 and standard deviation = 1 in AA (all three datasets combined) and EA target datasets separately.

### Statistical analysis

As COGA, SAGE, and YalePenn include related individuals, generalized linear mixed models were used with a random effect to adjust for family relationships. For Indiana Biobank, which is a cohort of unrelated individuals, logistic regression models were used. We also stratified individuals based on PRS deciles and compared each to the bottom decile. Since the sample sizes in COGA, SAGE, and YalePenn had insufficient sample sizes in each decile, we combined all three target datasets for the stratified analyses. For all models, sex and the first 10 PCs were included as covariates. For the combined analysis of COGA, SAGE, and YalePenn data, we also included the cohort indicator as an additional covariate. Associations with *P*-values < 0.05 across all three target datasets were considered statistically significant for PRS_gene_, PRS_integenic_, and PRS_all,_ respectively.

### Gene enrichment analyses, searching GWAS catalog and potential drug target genes

For genes included in calculating PRS_gene_, we performed gene ontology enrichment analysis using PANTHER (released 2021-01-24) [46] implemented in the Gene Ontology (GO) Resource (http://geneontology.org/, released 2021-08-18). We focused on GO Biological Processes (GOBPs). We also used Functional Mapping and Annotation of Genome-Wide Association Studies (FUMA) [47] to test whether these genes were enriched in Differentially Expressed Gene (DEG) sets calculated using 54 tissues from The Genotype-Tissue Expression (GTEx V8) project [48]. We searched the GWAS catalog (https://www.ebi.ac.uk/gwas/. accessed: 2021-10-11) [49] to check whether these genes had been previously implicated in GWAS of AUD-related phenotypes. Lastly, we checked whether these genes could be potential drug targets by searching the gene list for targets of the FDA-approved drugs as well as those in current clinical trial investigations, compiled by Wang et al. [24] derived from the Informa Pharmaprojects database (https://pharmaintelligence.informa.com/products-and-services/data-and-analysis/pharmaprojects).

## Results

Samples used in this study are summarized in Table 1. In all target datasets, about 60% of cases were males while <42% of controls were males.

**Table 1:** Sample summary.

Population	Target dataset	# case (%male)	# control (%male)	# total	# families
AA	All^a^	2786 (60.80)	3529 (39.27)	6315	3322
	COGA	875 (62.06)	2500 (41.60)	3375	590
	SAGE	387 (59.17)	543 (37.02)	930	869
	YalePenn	1524 (60.50)	486 (29.84)	2010	1863
EA	Indiana Biobank	539 (62.15)	3515 (40.40)	4054	4054

^a^COGA, SAGE, and YalePenn combined.

PRS-CSx estimated posterior meta-analysis effect sizes for 1 126 428 variants and they were used to calculate PRS_all_. In total, there were 1 533 variants with *P*-values < 0.05 in both EA-PAU and AA-AUD and having the same directions of effects (i.e., concordant). Among them, 858 (Table S1) and 675 (Table S3) variants were located within (410 genes, Table S2) and outside gene boundaries, respectively. As shown in Table 2, for AA target datasets, both PRS_gene_ and PRS_all_ had *P*-values < 0.05 in all target datasets except PRS_all_ for COGA. PRS_intergnic_ had *P*-values ≥ 0.10 in all target datasets, demonstrating that concordant variants located within genes better stratify risk for AUD than those located in intergenic regions. Effect sizes ranged from 0.15-0.21 for PRS_gene,_ −0.02 to 0.12 for PRS_intergenic_, and 0.06-0.18 for PRS_all_, respectively. In EA, 847 of 858 variants (Table S1), and 1 061 130 of 1 126 428 variants were present in Indiana Biobank after QC; both PRS_gene_ and PRS_all_ had *P*-values < 0.05 (PRS_gene_ Beta=0.11, SE = 0.02; PRS_all_ Beta = 0.34, SE = 0.05) but not PRS_intergenic_ (Beta = 0.02, SE = 0.05). Results of using different window sizes to extend gene boundaries are in Table S4 and Fig. S1. The numbers of variants increased slightly with larger window sizes, and windows 50 and 100 Mb had the same number of variants, indicating that most variants are located within or close to genes. Overall, the results were similar, therefore, we kept variants within gene boundaries because it was more straightforward to determine AUD genes, as larger distances often contained multiple genes, and it is challenging to assign intergenic variants to a gene.

**Table 2:** Associations between AUD and PRS_gene,_ PRS_intergenic_, and PRS_all_ in AA and EA.

		PRS_gene_				PRS_intergenic_				PRS_all_			
Population	Target dataset	Beta	SE	*P*-value	# Variants	Beta	SE	*P*-value	# Variants	Beta	SE	*P*-value	# Variants
AA	All^a^	0.17	0.03	**3.55E−08**	858	0.03	0.03	0.32	675	0.12	0.03	**9.42E−05**	1,126,428
	COGA	0.15	0.04	**9.67E−04**	858	0.02	0.04	0.61	675	0.06	0.04	0.16	1,126,428
	SAGE	0.18	0.07	**6.27E−03**	858	0.12	0.07	0.10	675	0.18	0.07	**0.01**	1,126,428
	YalePenn	0.21	0.05	**7.61E−05**	858	−0.02	0.06	0.76	675	0.17	0.06	**7.28E−03**	1,126,428
EA	Indiana Biobank	0.11	0.05	**0.02**	847	0.02	0.05	0.59	666	0.34	0.05	**2.35E−21**	1,061,130

Significant *P*-values are in bold.

*PRS*_*gene*_ PRS calculated using concordant variants located in genes associated with AUD in both AA and EA, *PRS*_*intergenic*_ PRS calculated using concordant variants located outside genes associated with AUD in both AA and EA, *PRS*_*all*_ PRS calculated using all variants.

^a^COGA, SAGE, and YalePenn combined.

**Fig. 1:**
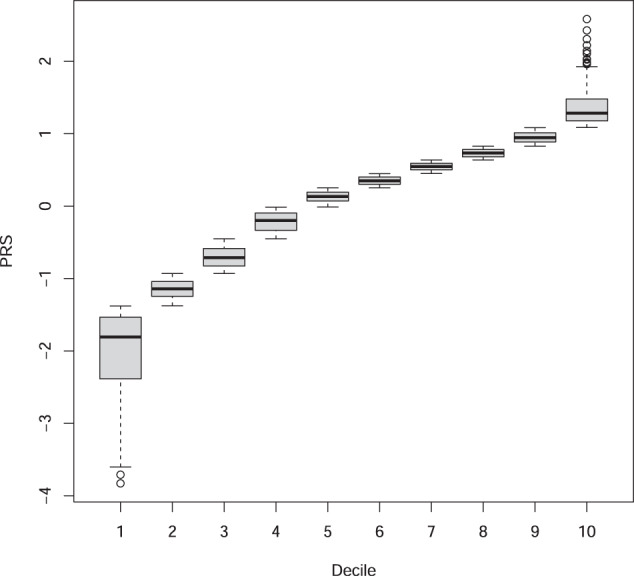
Distributions of PRS_gene_ in each decile in AA. Box-plots of each PRS_gene_ decile.

The association between PRS and AUD increased from the bottom decile (1st decile) to the top decile (10th decile) (Fig. 1). Using the bottom decile as the reference group, all except the 2nd and 3rd deciles showed statistically significant association with the increased odds for AUD (ORs: 1.37–1.76. Table 3) after adjusting for covariates.

**Table 3:** Odds ratios when comparing each PRS_gene_ decile with the bottom decile in AA (COGA, SAGE, and YalePenn combined).

Decile	OR	OR 95%CI	*P*-value
10	1.76	1.32–2.34	**1.03E−04**
9	1.72	1.29–2.29	**2.05E−04**
8	1.52	1.15–2.00	**2.93E−03**
7	1.64	1.27–2.12	**1.49E−04**
6	1.43	1.09–1.87	**0.01**
5	1.37	1.05–1.79	**0.02**
4	1.48	1.13–1.93	**4.55E−03**
3	1.22	0.91–1.62	0.18
2	1.11	0.85–1.45	0.44

Significant *P*-values are in bold.

Of the 410 genes, 353 were uniquely mapped to the GO database. The unmapped genes were non-coding RNAs, anti-sense RNAs, pseudo-genes, and read-throughs. Fifty-four GOBPs had false discovery rate (FDR) *P*-values < 0.05, including ethanol oxidation, synaptic signaling, synapse organization, synaptic plasticity, startle response, neurogenesis, nervous system development, learning or memory, protein metabolic process, cell adhesion, cell development, cell junction organization, movement of cell or subcellular component, cell-cell signaling, regulation of signaling, etc. (Table S5). Three hundred and seventy-four genes were mapped to GTEx V8 DEG sets and enrichment results are in Fig. S2. The majority of the enrichment sets were found in brain tissues but also included liver, kidney, and other tissues as well (Fig. S2).

Only 47 genes were identified in the previous GWAS of AUD-related phenotypes (Table S2). Twenty-six genes were targets of drugs approved by the FDA or in clinical trials. Among them, four (*DRD2*, *PDE4B*, *GRM5*, and *SLC6A9*) were drug target genes for AUD treatment (Table S6); for those 22 genes that were targets of drugs to treat diseases other than AUD, 21 were involved in the significant GOBPs identified and five (*EIF4E*, *ESR1*, *MAPT*, *METAP1*, *and TNKS*) were reported by previous GWAS of AUD-related phenotypes (Table S2).

## Discussion

In this study, we found that gene-based PRS (PRS_gene_) calculated using 858 variants from 410 genes were significantly associated with AUD in both AA and EA, and outperformed the PRS calculated using all variants (PRS_all_) in AA. Compared to the bottom decile, those at the top PRS_gene_ decile were nearly twice as likely to be AUD cases (OR = 1.76) in AA. The 410 genes included in calculating PRS_gene_ were enriched in 54 GOBPs, and many of them are likely to be AUD-related. They were also enriched in brain tissues. In addition, four genes were targets of drugs in Phase II or III clinical trials to treat AUD; 22 genes were targets of drugs approved by the FDA or in clinical trials to treat other diseases but may be repurposed to treat AUD. Together, these findings showed that biologically meaningful polygenic scores can be characterized in non-European ancestry individuals by leveraging methods that focus on intragenic signals with concordant directions of effects across ancestries. Furthermore, the process identified drugs already under development that could be evaluated for their potential to treat AUD.

To improve the performance of PRS, more disease-associated variants should be included and unrelated variants should be excluded. AUD is caused by many genes with small effects and in GWAS of AUD, due to the large number of variants tested, many variants that are unrelated to AUD show some degree of association (e.g., *P*-values < 0.05) purely by chance (i.e., false positives). If sample sizes are large (e.g., hundreds of thousands of participants or more), while the majority of AUD-associated variants are still not genome-wide significant, they usually have smaller *P*-values than those false positives and can still contribute to the calculation of PRS. However, when the discovery GWAS sample sizes are small to moderate, the discrimination between AUD-related and unrelated variants narrow. This leads to a reduction in PRS performance. Using large-scale EA discovery GWAS could mitigate this problem, but the improvement is limited even with sophisticated statistical methods due to the differences between the discovery GWAS and the target datasets [23]. Our gene-based PRS framework leverages the concordant variants across different populations and discriminates variants unrelated to the disease of interest leading to the improved performance of PRS. Using concordant variants also reduces the chance of selecting the wrong independent index variants due to a mismatch of LD patterns among the discovery and target datasets, as well as the external LD reference panels. Moreover, as PRS_intergenic_ were not associated with AUD in our analyses, the performance of PRS_gene_ was further improved by focusing on concordant variants within gene boundaries. RRS_gene_ had superior performance in all our AA target datasets, thus, we conclude that this strategy can be used to improve the performance of PRS when the discovery GWAS sample sizes are not sufficiently large, notable in admixture populations, and other groups that have been underrepresented in GWAS studies to date.

While PRS_gene_ outperformed PRS_all_ in AA, the opposite was observed in EA. This was expected for the following reasons. First, many GWAS findings, such as variants in *KLB* and *GCKR*, which reached genome-wide significance in EA, had *P*-values > 0.05 in AA (i.e., these genes may not be AUD-related in AA for some unknown mechanisms, or variants acting on these genes in AA have not been identified), therefore, they were not included in calculating PRS_gene_ but were used in calculating PRS_all_ in EA. Second, even within genes that have shown associations with AUD in both AA and EA, different causal variants may have been important in each ancestral group. One example is rs2066702 in the *ADH1B* gene. While relatively common in AA individuals (MAF = 0.18), the variant is rare in EA individuals (MAF = 0.002) (https://www.ncbi.nlm.nih.gov/snp/rs2066702?vertical_tab=true#frequency_tab). This was the only variant selected in *ADH1B* in calculating PRS_gene_, resulting in no contribution of *ADH1B* when calculating PRS_gene_ in EA individuals from the Indiana Biobank. However, for PRS_all_, multiple common EA variants in *ADH1B* (e.g., rs2066701, rs1042026, and rs2075633) were included, thus increasing the performance of PRS_all_. Third, we limited inclusion to variants within gene boundaries. Although PRS_intergenic_ and PRS_gene_ with extended boundaries analyses showed that overall including intergenic concordant variants did not increase the PRS performance, however, some AUD variants are not located within gene boundaries and this may affect AA and EA disproportionately. For example, rs1229978, which is located between *ADH1B* and *ADH1C*, is much more common in EA (MAF = 0.39) than in AA (MAF = 0.15) (https://www.ncbi.nlm.nih.gov/snp/rs1229978?vertical_tab=true); therefore, not including this variant in PRS calculations had a larger impact in EA than in AA. Nevertheless, the significance of PRS_gene_ in both AA and EA suggested that most of these genes were AUD-related in these two populations.

More than half of the 410 genes (244) were involved in 54 significant GOBPs. As expected, ethanol oxidation was among them and four genes (*ADH1B, ADH1C, ADH4*, and *ADH5*) were involved. Compromised executive functioning (i.e., neuroadaptation) is one of the major mechanisms contributing to AUD [50] and not surprisingly, several significant GOBPs related to synaptic systems (synaptic signaling, synapse organization, synaptic plasticity, startle response) were identified (46 genes). Although the role of the synaptic system in AUD is well-established [50], however, only nine genes (*CSMD1*, *DCC*, *DRD3*, *EIF4E*, *ERC2*, *LINGO2*, *MAPT*, *NRXN2*, and *TENM2*) were implicated in previous GWAS of AUD-related phenotypes. We also found significant GOBPs related to learning and memory (27 genes), consistent with previous findings that AUD and neurodegenerative diseases share some genetic liability [51]. Nervous system development-related GOBPs were significant (69 genes), and genes involved may predispose to AUD via mechanisms yet to be discovered. GOBPs such as protein metabolic process, cell adhesion, cell development, cell junction organization, movement of cell or subcellular component, cell-cell signaling, and regulation of signaling were also significant. Intuitively, these GOBPs may not seem to be AUD-related, however, among 148 genes only involved in these processes, 20 of them were reported in previous GWAS of AUD-related phenotypes with some of them, e.g., *FTO*, *PDE4B*, and *SLC39A8*, being genome-wide significant in recent large-scale GWAS of AUD [7]. In addition, there were seven genes (*EHBP1*, *EYS*, *FNBP4*, *LOC100507053*, *TNRC6A*, *WDR7*, and *ZNF462*) that were not involved in any significant GOBPs but were reported by previous GWAS of AUD-related phenotypes. Further studies are needed to elucidate the roles of these genes in predisposing to AUD. Tissue-specific enrichment showed that most genes were enriched in brain tissues as expected, however, other tissues such as liver, kidney, and pancreas also showed enrichment. Except liver, how these tissues relate to AUD remain to be discovered. By searching the drug target gene database, we found four genes (*DRD2*, *PDE4B*, *GRM5*, and *SLC6A9*) were already targets of AUD treatment drugs (Table S6). We also found 22 genes that were targets of drugs to treat other diseases (Table S6) and could be examined and/or repurposed to treat AUD. Studies have found that gene-targeted drugs were more likely to get FDA approval [24, 52, 53], therefore, identifying the roles of genes used in calculating PRS_gene_ could facilitate the development of novel treatment methods.

This study has several limitations. First, we limited to concordant variants in both AA and EA, thus, variants that may have discordant but true effects were excluded, reducing the performance of PRS_gene_. Second, although most intergenic concordant variants did not contribute to the PRS signal as shown in the analyses of PRS_intergenic_ and extended gene boundaries with different window sizes, some of them are truly AUD related and contribute to the risk of AUD, and excluding them leading to a further reduction in the performance of PRS_gene_. Third, we used posterior effects estimated from the meta-analysis of AA-MVP and EA-PAU. As EA-PAU had a much larger sample size (>7 times of sample size of AA-MVP), more weight was put on effects estimated from the EA samples. Therefore, for those variants that had different sizes of effects between AA and EA, effects from the meta-analysis were biased toward the EA GWAS. Fourth, studies have shown that using functional annotations can improve the performance of PRS and increase the transferability of PRS between different populations [11, 54–56]. However, most of the available functional annotation databases were generated using European ancestry samples and were not related to AUD, therefore, those functional information were not used in this study, which may also reduce the performance of PRS_gene_.

In summary, we calculated PRS for evaluating AUD risk that worked cross populations based on our novel gene-based PRS framework. Not only our new framework outperformed the PRS calculated using all variants in AA, but also the genes included in calculating PRS showed enrichment for biological plausible processes and are potential targets for drug development, therefore, this novel framework demonstrates the utilities of PRS beyond disease risk evaluation to the identification of biological processes and drug targets, and shed light on the genetic mechanism of AUD.

## Supplementary information

858 concordant variants located within gene boundaries.

410 genes included in calculating PRSgene.

675 intergenic concordant variants.

PRSgene calculated using different window sizes.

Gene ontology enrichment analysis results.

Drug target genes.

Negative logP-values of PRSgene using different window sizes to extend gene boundaries.

Tissue-specific Differentially Expressed Gene enrichment analysis.
