# Supplementary material for: Gene-based polygenic risk scores analysis of alcohol use disorder in African Americans
Source: Transl Psychiatry. 2022 Jul 5;12:266. doi: 10.1038/s41398-022-02029-2 (PMC9256707; doi:10.1038/s41398-022-02029-2)
Supplement: Supplementary file 1 — 858 concordant variants located within gene boundaries. [file 41398_2022_2029_MOESM1_ESM.docx]

**Table S1**: 858 concordant variants located within gene boundaries.

| chr | SNP | bp | Allele1 | Allelel2 | AA.Pvalue | EA.Pavlue | Function | Gene | Gene Detail | Exonic Function | Amino Acid Change | Not in IB |
| --- | --- | --- | --- | --- | --- | --- | --- | --- | --- | --- | --- | --- |
| 1 | rs2274976 | 11,850,927 | T | C | 0.04 | 0.01 | exonic | *MTHFR* | . | synonymous SNV | MTHFR:NM_001330358:exon12:c.G1904G:p.R635R,MTHFR:NM_005957:exon12:c.G1781G:p.R594R |  |
| 1 | rs17037397 | 11,862,163 | A | C | 0.04 | 0.02 | intronic | *MTHFR* | . | . | . |  |
| 1 | rs3767150 | 21,894,214 | A | G | 0.04 | 0.01 | intronic | *ALPL* | . | . | . |  |
| 1 | rs3738097 | 21,894,816 | C | T | 0.04 | 9.72E-03 | intronic | *ALPL* | . | . | . |  |
| 1 | rs3767145 | 21,895,802 | G | A | 0.04 | 6.82E-03 | intronic | *ALPL* | . | . | . |  |
| 1 | rs11210886 | 44,071,546 | A | G | 0.04 | 1.70E-03 | intronic | *PTPRF* | . | . | . |  |
| 1 | rs7523468 | 44,493,795 | A | G | 6.38E-03 | 1.45E-03 | intronic | *SLC6A9* | . | . | . |  |
| 1 | rs2485653 | 44,594,966 | A | G | 0.02 | 2.05E-03 | intronic | *KLF17* | . | . | . |  |
| 1 | rs369493 | 44,740,024 | T | C | 0.04 | 1.05E-06 | intronic | *ERI3* | . | . | . |  |
| 1 | rs370658 | 44,740,244 | A | G | 0.03 | 1.02E-06 | intronic | *ERI3* | . | . | . |  |
| 1 | rs401595 | 44,747,972 | T | C | 0.02 | 1.33E-06 | intronic | *ERI3* | . | . | . |  |
| 1 | rs1295803 | 44,757,425 | C | T | 0.05 | 1.63E-03 | intronic | *ERI3* | . | . | . |  |
| 1 | rs2301767 | 44,773,839 | T | C | 0.02 | 6.09E-07 | intronic | *ERI3* | . | . | . |  |
| 1 | rs12026967 | 44,776,240 | A | C | 2.01E-03 | 1.39E-06 | intronic | *ERI3* | . | . | . |  |
| 1 | rs2074536 | 44,785,686 | T | C | 0.02 | 9.48E-07 | intronic | *ERI3* | . | . | . |  |
| 1 | rs12739892 | 66,304,567 | T | G | 0.04 | 3.39E-04 | intronic | *PDE4B* | . | . | . |  |
| 1 | rs4077431 | 66,309,392 | A | C | 6.66E-04 | 6.17E-06 | intronic | *PDE4B* | . | . | . |  |
| 1 | rs1316871 | 66,339,048 | C | T | 5.95E-03 | 1.57E-03 | intronic | *PDE4B* | . | . | . |  |
| 1 | rs1937456 | 66,416,747 | T | C | 0.02 | 3.39E-06 | intronic | *PDE4B* | . | . | . |  |
| 1 | rs1937455 | 66,416,939 | G | A | 2.01E-03 | 4.34E-10 | intronic | *PDE4B* | . | . | . |  |
| 1 | rs2228693 | 66,509,014 | C | A | 0.03 | 2.09E-08 | ncRNA_intronic | *PDE4B-AS1* | . | . | . |  |
| 1 | rs1500950 | 66,522,271 | A | G | 0.04 | 9.68E-04 | intronic | *PDE4B* | . | . | . |  |
| 1 | rs1500963 | 66,529,821 | A | G | 0.04 | 4.50E-03 | intronic | *PDE4B* | . | . | . |  |
| 1 | rs1500958 | 66,539,046 | A | G | 0.01 | 9.82E-04 | intronic | *PDE4B* | . | . | . |  |
| 1 | rs6424546 | 73,776,673 | G | A | 0.02 | 1.03E-06 | ncRNA_exonic | *LINC01360* | . | . | . |  |
| 1 | rs11210205 | 73,778,100 | C | T | 0.02 | 1.29E-06 | ncRNA_intronic | *LINC01360* | . | . | . |  |
| 1 | rs12061680 | 73,778,378 | G | A | 0.02 | 1.18E-06 | ncRNA_intronic | *LINC01360* | . | . | . |  |
| 1 | rs7517355 | 73,784,077 | G | A | 0.02 | 1.15E-06 | ncRNA_intronic | *LINC01360* | . | . | . |  |
| 1 | rs11210209 | 73,793,245 | A | G | 0.03 | 8.89E-07 | ncRNA_intronic | *LINC01360* | . | . | . |  |
| 1 | rs12403583 | 77,095,355 | G | A | 4.38E-03 | 0.02 | UTR3 | *ST6GALNAC3* | NM_001349106:c.*864G>A;NM_152996:c.*864G>A;NM_001349105:c.*864G>A;NM_001349111:c.*864G>A;NM_001349108:c.*864G>A;NM_001349107:c.*864G>A | . | . |  |
| 1 | rs9970671 | 77,095,600 | A | G | 4.37E-03 | 0.02 | UTR3 | *ST6GALNAC3* | NM_001349106:c.*1109A>G;NM_152996:c.*1109A>G;NM_001349105:c.*1109A>G;NM_001349111:c.*1109A>G;NM_001349108:c.*1109A>G;NM_001349107:c.*1109A>G | . | . |  |
| 1 | rs12404937 | 77,099,075 | G | A | 1.82E-03 | 0.02 | UTR3 | *ST6GALNAC3* | NM_001349106:c.*4584G>A;NM_152996:c.*4584G>A;NM_001349105:c.*4584G>A;NM_001349111:c.*4584G>A;NM_001349108:c.*4584G>A;NM_001349107:c.*4584G>A | . | . |  |
| 1 | rs9287139 | 89,237,078 | G | A | 0.04 | 0.04 | intronic | *PKN2* | . | . | . |  |
| 1 | rs12040070 | 100,172,901 | A | G | 0.04 | 0.04 | ncRNA_intronic | *MIR548N* | . | . | . |  |
| 1 | rs12037569 | 109,923,677 | T | G | 0.01 | 0.04 | intronic | *SORT1* | . | . | . |  |
| 1 | rs12049330 | 110,031,188 | G | T | 0.02 | 0.02 | intronic | *ATXN7L2* | . | . | . |  |
| 1 | rs4539125 | 117,482,765 | T | C | 0.03 | 0.01 | intronic | *PTGFRN* | . | . | . |  |
| 1 | rs4641299 | 117,483,361 | A | G | 0.03 | 0.01 | intronic | *PTGFRN* | . | . | . |  |
| 1 | rs1146297 | 117,910,682 | G | A | 0.03 | 6.50E-04 | UTR5 | *MAN1A2* | NM_006699:c.-124G>A | . | . |  |
| 1 | rs6678854 | 119,729,171 | G | T | 0.03 | 7.70E-03 | ncRNA_intronic | *WARS2-AS1* | . | . | . |  |
| 1 | rs1891222 | 119,751,002 | C | T | 2.78E-03 | 0.03 | ncRNA_intronic | *WARS2-AS1* | . | . | . |  |
| 1 | rs2481084 | 160,989,614 | C | T | 0.03 | 0.04 | intronic | *F11R* | . | . | . |  |
| 1 | rs3737787 | 161,009,523 | A | G | 0.02 | 0.03 | UTR3 | *USF1* | NM_007122:c.*187T>C;NM_001276373:c.*187T>C;NM_207005:c.*187T>C | . | . |  |
| 1 | rs2073655 | 161,012,590 | A | G | 0.02 | 0.03 | intronic | *USF1* | . | . | . |  |
| 1 | rs2516839 | 161,013,121 | T | C | 2.88E-03 | 4.33E-03 | UTR5 | *USF1* | NM_007122:c.-56A>G;NM_001276373:c.-56A>G;NM_207005:c.-1117A>G | . | . |  |
| 1 | rs2516837 | 161,014,727 | G | A | 1.24E-03 | 4.41E-03 | UTR5 | *USF1* | NM_001276373:c.-1662C>T | . | . |  |
| 1 | rs2661319 | 163,039,777 | C | T | 0.01 | 0.01 | intronic | *RGS4* | . | . | . |  |
| 1 | rs2789442 | 164,732,838 | G | A | 0.03 | 1.49E-03 | intronic | *PBX1* | . | . | . |  |
| 1 | rs2789444 | 164,734,457 | G | A | 0.02 | 1.19E-03 | intronic | *PBX1* | . | . | . |  |
| 1 | rs203797 | 167,819,529 | G | A | 0.05 | 3.57E-03 | intronic | *ADCY10* | . | . | . |  |
| 1 | rs4652298 | 173,858,524 | T | C | 0.04 | 0.01 | UTR3 | *ZBTB37* | NM_001346115:c.*3747T>C;NM_001369846:c.*3747T>C;NM_001122770:c.*3262T>C;NM_032522:c.*15757T>C | . | . |  |
| 1 | rs9425431 | 173,862,626 | T | C | 0.05 | 0.01 | UTR3 | *ZBTB37* | NM_001346115:c.*7849T>C;NM_001369846:c.*7849T>C;NM_001122770:c.*7364T>C;NM_032522:c.*19859T>C | . | . |  |
| 1 | rs9425433 | 173,864,880 | T | C | 0.04 | 0.01 | UTR3 | *ZBTB37* | NM_001346115:c.*10103T>C;NM_001369846:c.*10103T>C;NM_001122770:c.*9618T>C;NM_032522:c.*22113T>C | . | . |  |
| 1 | rs16847812 | 175,049,379 | A | G | 0.05 | 1.06E-03 | exonic | *TNN* | . | synonymous SNV | TNN:NM_022093:exon4:c.G865G:p.D289D |  |
| 1 | rs12126360 | 179,148,739 | C | T | 0.05 | 8.90E-03 | intronic | *ABL2* | . | . | . |  |
| 1 | rs12120194 | 179,154,457 | G | A | 0.03 | 7.03E-03 | intronic | *ABL2* | . | . | . |  |
| 1 | rs2078087 | 183,358,405 | T | C | 0.01 | 4.33E-04 | intronic | *NMNAT2* | . | . | . |  |
| 1 | rs10494566 | 183,690,467 | A | G | 0.04 | 0.04 | intronic | *RGL1* | . | . | . |  |
| 1 | rs10158410 | 198,150,496 | T | G | 7.29E-03 | 0.04 | intronic | *NEK7* | . | . | . |  |
| 1 | rs16842548 | 198,158,066 | G | A | 0.01 | 0.02 | intronic | *NEK7* | . | . | . |  |
| 1 | rs13375334 | 198,207,105 | G | A | 0.01 | 0.03 | intronic | *NEK7* | . | . | . |  |
| 1 | rs6688481 | 198,225,658 | C | T | 0.01 | 0.04 | intronic | *NEK7* | . | . | . |  |
| 1 | rs16842626 | 198,226,201 | T | C | 0.01 | 0.04 | intronic | *NEK7* | . | . | . |  |
| 1 | rs6668469 | 198,249,195 | C | T | 0.01 | 0.04 | intronic | *NEK7* | . | . | . |  |
| 1 | rs10494768 | 198,252,452 | T | G | 0.01 | 0.04 | intronic | *NEK7* | . | . | . |  |
| 1 | rs716784 | 198,254,389 | T | C | 0.01 | 0.05 | intronic | *NEK7* | . | . | . |  |
| 1 | rs7546420 | 198,259,680 | A | G | 0.02 | 0.03 | intronic | *NEK7* | . | . | . |  |
| 1 | rs10801677 | 198,628,483 | G | A | 0.03 | 0.03 | intronic | *PTPRC* | . | . | . |  |
| 1 | rs4128458 | 201,355,522 | T | C | 7.91E-03 | 0.05 | exonic | *LAD1* | . | nonsynonymous SNV | LAD1:NM_005558:exon3:c.A967G:p.K323E |  |
| 1 | rs6696846 | 205,041,952 | T | C | 0.04 | 0.04 | intronic | *CNTN2* | . | . | . |  |
| 1 | rs12410558 | 208,366,991 | T | C | 0.05 | 0.03 | intronic | *PLXNA2* | . | . | . |  |
| 1 | rs1609841 | 208,367,614 | A | G | 0.04 | 0.02 | intronic | *PLXNA2* | . | . | . |  |
| 1 | rs12132749 | 215,845,310 | C | T | 0.01 | 0.02 | intronic | *USH2A* | . | . | . |  |
| 1 | rs7532704 | 217,971,980 | T | C | 9.31E-03 | 0.01 | intronic | *SPATA17* | . | . | . |  |
| 1 | rs10926656 | 242,382,719 | G | A | 0.04 | 0.01 | intronic | *PLD5* | . | . | . |  |
| 1 | rs10926664 | 242,388,229 | G | A | 0.03 | 0.01 | intronic | *PLD5* | . | . | . |  |
| 1 | rs2653159 | 244,815,816 | G | A | 0.01 | 0.03 | upstream | *DESI2* | dist=612 | . | . |  |
| 1 | rs12035845 | 246,356,750 | T | C | 2.23E-03 | 0.04 | intronic | *SMYD3* | . | . | . |  |
| 2 | rs10929456 | 7,025,175 | C | T | 0.02 | 0.03 | intronic | *RSAD2* | . | . | . |  |
| 2 | rs16865717 | 7,032,618 | C | T | 0.02 | 0.02 | intronic | *RSAD2* | . | . | . |  |
| 2 | rs7081 | 27,422,605 | C | T | 0.03 | 5.40E-03 | UTR3 | *SLC5A6* | NM_021095:c.*699G>A | . | . |  |
| 2 | rs1275525 | 27,428,702 | A | C | 0.05 | 5.32E-03 | intronic | *SLC5A6* | . | . | . |  |
| 2 | rs12472538 | 44,566,872 | A | C | 8.14E-03 | 0.03 | intronic | *PREPL* | . | . | . |  |
| 2 | rs13018198 | 48,824,698 | C | T | 0.03 | 0.02 | UTR3 | *STON1* | NM_001198595:c.*2257C>T;NM_006873:c.*2257C>T | . | . |  |
| 2 | rs2678902 | 58,138,581 | A | G | 0.02 | 1.30E-05 | intronic | *VRK2* | . | . | . |  |
| 2 | rs2717025 | 58,163,146 | T | G | 6.34E-03 | 4.32E-06 | intronic | *VRK2* | . | . | . |  |
| 2 | rs2717026 | 58,163,178 | G | A | 6.32E-03 | 4.48E-06 | intronic | *VRK2* | . | . | . |  |
| 2 | rs2717038 | 58,168,831 | G | A | 7.79E-03 | 3.30E-06 | intronic | *VRK2* | . | . | . |  |
| 2 | rs2717039 | 58,169,166 | T | G | 1.49E-03 | 3.89E-06 | intronic | *VRK2* | . | . | . |  |
| 2 | rs7561688 | 58,233,464 | T | C | 0.04 | 0.05 | intronic | *VRK2* | . | . | . |  |
| 2 | rs1051061 | 58,316,814 | G | A | 0.01 | 2.10E-03 | exonic | *VRK2* | . | synonymous SNV | VRK2:NM_001288839:exon6:c.A145A:p.I49I,VRK2:NM_001130480:exon7:c.A499A:p.I167I,VRK2:NM_001130481:exon7:c.A499A:p.I167I,VRK2:NM_001130482:exon7:c.A430A:p.I144I,VRK2:NM_001130483:exon7:c.A499A:p.I167I,VRK2:NM_006296:exon7:c.A499A:p.I167I,VRK2:NM_001288836:exon8:c.A145A:p.I49I,VRK2:NM_001288838:exon9:c.A499A:p.I167I,VRK2:NM_001288837:exon10:c.A499A:p.I167I |  |
| 2 | rs17049334 | 58,326,448 | G | A | 0.04 | 6.07E-03 | intronic | *VRK2* | . | . | . |  |
| 2 | rs1016771 | 58,335,681 | C | T | 8.20E-03 | 1.80E-03 | intronic | *VRK2* | . | . | . |  |
| 2 | rs13011711 | 58,338,086 | G | A | 2.72E-03 | 3.19E-03 | intronic | *VRK2* | . | . | . |  |
| 2 | rs17049351 | 58,346,966 | G | A | 0.04 | 5.76E-03 | intronic | *VRK2* | . | . | . |  |
| 2 | rs3771211 | 58,354,422 | G | A | 0.03 | 5.98E-03 | intronic | *VRK2* | . | . | . |  |
| 2 | rs10445895 | 58,390,538 | C | T | 0.01 | 3.30E-03 | intronic | *FANCL* | . | . | . |  |
| 2 | rs2592494 | 61,381,528 | C | T | 0.02 | 0.02 | intronic | *C2orf74* | . | . | . |  |
| 2 | rs10169662 | 61,507,936 | C | T | 0.05 | 0.05 | intronic | *USP34* | . | . | . |  |
| 2 | rs2694618 | 61,536,984 | T | C | 7.65E-03 | 0.05 | intronic | *USP34* | . | . | . |  |
| 2 | rs7582882 | 62,727,154 | T | C | 9.57E-03 | 4.92E-04 | downstream | *TMEM17* | dist=200 | . | . |  |
| 2 | rs13396048 | 62,728,105 | G | A | 9.49E-03 | 3.72E-04 | UTR3 | *TMEM17* | NM_198276:c.*239C>T | . | . |  |
| 2 | rs4672527 | 62,729,674 | T | C | 0.02 | 4.08E-04 | exonic | *TMEM17* | . | synonymous SNV | TMEM17:NM_198276:exon3:c.A216G:p.L72L |  |
| 2 | rs11903221 | 62,730,506 | C | T | 0.02 | 4.14E-04 | intronic | *TMEM17* | . | . | . |  |
| 2 | rs13027462 | 63,154,816 | G | A | 0.02 | 0.03 | intronic | *EHBP1* | . | . | . |  |
| 2 | rs12713488 | 63,586,777 | T | C | 0.05 | 1.26E-03 | intronic | *WDPCP* | . | . | . |  |
| 2 | rs1922422 | 63,593,212 | T | C | 0.02 | 1.13E-03 | intronic | *WDPCP* | . | . | . |  |
| 2 | rs1356388 | 63,595,981 | T | C | 0.04 | 1.23E-03 | intronic | *WDPCP* | . | . | . |  |
| 2 | rs11548392 | 65,539,596 | A | G | 0.04 | 0.02 | UTR3 | *SPRED2* | NM_001128210:c.*1039T>C;NM_181784:c.*1039T>C | . | . |  |
| 2 | rs10181915 | 78,316,017 | T | C | 0.03 | 0.05 | ncRNA_intronic | *LOC101927948;LOC101927967* | . | . | . |  |
| 2 | rs1918693 | 84,938,981 | C | T | 0.02 | 0.05 | intronic | *DNAH6* | . | . | . |  |
| 2 | rs10199591 | 84,946,063 | A | G | 0.04 | 0.05 | intronic | *DNAH6* | . | . | . |  |
| 2 | rs17713693 | 85,512,062 | G | A | 0.04 | 0.01 | intronic | *TCF7L1* | . | . | . |  |
| 2 | rs11687241 | 100,824,595 | G | A | 0.03 | 0.03 | upstream | *LINC01104* | dist=121 | . | . |  |
| 2 | rs6725672 | 101,020,624 | A | G | 0.04 | 0.04 | intronic | *CHST10* | . | . | . |  |
| 2 | rs2242290 | 103,585,422 | C | T | 0.05 | 0.02 | ncRNA_exonic | *LINC01935* | . | . | . |  |
| 2 | rs4662776 | 128,870,952 | C | T | 0.05 | 0.05 | intronic | *UGGT1* | . | . | . |  |
| 2 | rs13015001 | 145,440,840 | A | G | 4.75E-04 | 0.02 | ncRNA_intronic | *TEX41* | . | . | . |  |
| 2 | rs10496966 | 145,458,762 | G | A | 1.21E-03 | 0.03 | ncRNA_intronic | *TEX41* | . | . | . |  |
| 2 | rs733659 | 161,119,392 | A | G | 0.04 | 0.03 | ncRNA_intronic | *LINC02478* | . | . | . |  |
| 2 | rs733658 | 161,119,413 | G | A | 0.04 | 0.03 | ncRNA_intronic | *LINC02478* | . | . | . |  |
| 2 | rs16845945 | 162,480,684 | T | C | 7.91E-03 | 0.02 | upstream | *SLC4A10* | dist=161 | . | . |  |
| 2 | rs12473088 | 162,485,073 | G | A | 6.56E-03 | 0.02 | intronic | *SLC4A10* | . | . | . |  |
| 2 | rs16845997 | 162,521,046 | A | C | 0.01 | 0.02 | intronic | *SLC4A10* | . | . | . |  |
| 2 | rs12467279 | 162,544,744 | G | A | 5.84E-03 | 0.02 | intronic | *SLC4A10* | . | . | . |  |
| 2 | rs16846050 | 162,563,553 | A | G | 6.74E-03 | 0.02 | intronic | *SLC4A10* | . | . | . |  |
| 2 | rs16846053 | 162,566,045 | G | T | 5.44E-03 | 0.02 | intronic | *SLC4A10* | . | . | . |  |
| 2 | rs12474713 | 162,569,341 | A | C | 7.41E-03 | 0.02 | intronic | *SLC4A10* | . | . | . |  |
| 2 | rs2892961 | 166,228,358 | A | G | 0.03 | 8.43E-03 | intronic | *SCN2A* | . | . | . |  |
| 2 | rs1448831 | 168,877,794 | C | A | 3.41E-04 | 0.03 | intronic | *STK39* | . | . | . |  |
| 2 | rs11685807 | 168,885,333 | A | C | 0.02 | 0.03 | intronic | *STK39* | . | . | . |  |
| 2 | rs13430810 | 169,090,774 | C | T | 0.03 | 1.37E-03 | intronic | *STK39* | . | . | . |  |
| 2 | rs4667591 | 170,003,432 | G | T | 2.87E-03 | 0.05 | exonic | *LRP2* | . | synonymous SNV | LRP2:NM_004525:exon69:c.A12628A:p.I4210I |  |
| 2 | rs2627037 | 179,606,538 | A | G | 0.04 | 0.02 | exonic | *TTN* | . | synonymous SNV | TTN:NM_003319:exon45:c.C10333C:p.P3445P,TTN:NM_001256850:exon46:c.C10471C:p.P3491P,TTN:NM_133432:exon46:c.C10708C:p.P3570P,TTN:NM_133437:exon46:c.C10909C:p.P3637P,TTN:NM_001267550:exon48:c.C11422C:p.P3808P |  |
| 2 | rs4077924 | 181,991,389 | T | C | 0.02 | 0.02 | ncRNA_intronic | *LINC01934* | . | . | . |  |
| 2 | rs4667082 | 181,999,977 | A | C | 0.04 | 0.02 | ncRNA_intronic | *LINC01934* | . | . | . |  |
| 2 | rs1344706 | 185,778,428 | C | A | 0.04 | 1.30E-04 | intronic | *ZNF804A* | . | . | . |  |
| 2 | rs1366842 | 185,802,243 | A | C | 0.01 | 7.18E-03 | exonic | *ZNF804A* | . | synonymous SNV | ZNF804A:NM_194250:exon4:c.C2120C:p.T707T |  |
| 2 | rs1517352 | 191,931,464 | C | A | 0.04 | 9.12E-03 | intronic | *STAT4* | . | . | . |  |
| 2 | rs2350722 | 202,964,528 | T | C | 0.05 | 5.26E-03 | ncRNA_intronic | *KIAA2012-AS1* | . | . | . |  |
| 2 | rs6755553 | 202,965,360 | C | T | 0.05 | 4.06E-03 | ncRNA_intronic | *KIAA2012-AS1* | . | . | . |  |
| 2 | rs13393577 | 213,296,863 | C | T | 0.04 | 0.04 | intronic | *ERBB4* | . | . | . |  |
| 2 | rs10180608 | 228,987,075 | G | T | 0.04 | 0.02 | intronic | *SPHKAP* | . | . | . |  |
| 2 | rs12615058 | 228,989,843 | A | G | 0.04 | 0.02 | intronic | *SPHKAP* | . | . | . |  |
| 2 | rs12615089 | 228,989,962 | A | G | 0.04 | 0.02 | intronic | *SPHKAP* | . | . | . |  |
| 2 | rs4312487 | 228,990,498 | G | A | 0.04 | 0.02 | intronic | *SPHKAP* | . | . | . |  |
| 2 | rs6436754 | 228,999,542 | C | T | 0.04 | 0.02 | intronic | *SPHKAP* | . | . | . |  |
| 2 | rs6436758 | 229,000,048 | G | A | 0.03 | 0.02 | intronic | *SPHKAP* | . | . | . |  |
| 2 | rs4246656 | 229,006,078 | T | C | 0.03 | 0.03 | intronic | *SPHKAP* | . | . | . |  |
| 2 | rs1973675 | 233,636,450 | A | G | 0.04 | 0.01 | intronic | *GIGYF2;KCNJ13* | . | . | . |  |
| 2 | rs10211596 | 233,699,415 | A | G | 0.03 | 1.28E-03 | intronic | *GIGYF2* | . | . | . |  |
| 2 | rs1967369 | 237,130,690 | T | C | 0.03 | 0.03 | intronic | *ASB18* | . | . | . |  |
| 2 | rs1530952 | 237,131,159 | C | A | 0.03 | 0.03 | intronic | *ASB18* | . | . | . |  |
| 2 | rs6756597 | 237,149,941 | T | C | 9.41E-03 | 0.05 | exonic | *ASB18* | . | synonymous SNV | ASB18:NM_212556:exon2:c.G310G:p.A104A |  |
| 3 | rs17586876 | 2,964,182 | G | A | 0.04 | 4.31E-03 | intronic | *CNTN4* | . | . | . |  |
| 3 | rs6764411 | 7,143,551 | T | G | 6.78E-04 | 0.02 | intronic | *GRM7* | . | . | . |  |
| 3 | rs1876611 | 8,603,483 | A | C | 0.02 | 0.03 | intronic | *LMCD1* | . | . | . |  |
| 3 | rs696513 | 9,783,147 | G | A | 0.05 | 0.03 | intronic | *BRPF1* | . | . | . |  |
| 3 | rs713178 | 12,615,984 | C | T | 0.02 | 0.03 | intronic | *MKRN2* | . | . | . |  |
| 3 | rs9876540 | 45,162,316 | T | C | 0.03 | 9.85E-04 | intronic | *CDCP1* | . | . | . |  |
| 3 | rs7610357 | 45,527,218 | C | T | 0.03 | 0.04 | exonic | *LARS2* | . | synonymous SNV | LARS2:NM_001368263:exon10:c.T1053T:p.L351L,LARS2:NM_015340:exon11:c.T1053T:p.L351L |  |
| 3 | rs7612869 | 45,527,484 | T | C | 0.01 | 0.02 | ncRNA_intronic | *LARS2-AS1* | . | . | . |  |
| 3 | rs4312689 | 45,528,633 | T | G | 0.03 | 0.05 | ncRNA_intronic | *LARS2-AS1* | . | . | . |  |
| 3 | rs2128361 | 45,533,224 | A | G | 0.03 | 0.04 | exonic | *LARS2* | . | synonymous SNV | LARS2:NM_001368263:exon12:c.G1455G:p.A485A,LARS2:NM_015340:exon13:c.G1455G:p.A485A |  |
| 3 | rs1482466 | 45,533,745 | A | G | 0.03 | 0.04 | ncRNA_intronic | *LARS2-AS1* | . | . | . |  |
| 3 | rs3774685 | 45,535,474 | G | A | 0.02 | 0.03 | ncRNA_intronic | *LARS2-AS1* | . | . | . |  |
| 3 | rs9311371 | 45,546,656 | G | A | 0.02 | 0.02 | ncRNA_intronic | *LARS2-AS1* | . | . | . |  |
| 3 | rs9859473 | 48,837,182 | T | C | 0.05 | 8.36E-05 | intronic | *PRKAR2A* | . | . | . |  |
| 3 | rs7653408 | 48,972,351 | A | C | 0.05 | 7.77E-05 | intronic | *ARIH2* | . | . | . |  |
| 3 | rs9864243 | 48,982,732 | G | A | 0.05 | 8.42E-05 | intronic | *ARIH2* | . | . | . |  |
| 3 | rs7434187 | 49,032,967 | C | T | 0.05 | 1.16E-04 | intronic | *P4HTM* | . | . | . |  |
| 3 | rs6794010 | 55,615,260 | G | A | 0.01 | 0.04 | intronic | *ERC2* | . | . | . |  |
| 3 | rs13084153 | 62,055,170 | T | C | 0.03 | 0.05 | intronic | *PTPRG* | . | . | . |  |
| 3 | rs3772902 | 81,723,365 | G | A | 0.05 | 1.10E-03 | intronic | *GBE1* | . | . | . |  |
| 3 | rs9859600 | 99,387,903 | G | A | 0.03 | 3.75E-03 | intronic | *COL8A1* | . | . | . |  |
| 3 | rs9883702 | 99,397,141 | C | T | 0.03 | 3.43E-03 | intronic | *COL8A1* | . | . | . |  |
| 3 | rs17313599 | 99,641,479 | C | A | 0.04 | 0.02 | intronic | *CMSS1;FILIP1L* | . | . | . |  |
| 3 | rs2303473 | 101,404,813 | G | A | 7.43E-03 | 0.01 | intronic | *RPL24* | . | . | . |  |
| 3 | rs6808592 | 108,055,847 | G | T | 0.02 | 0.03 | intronic | *HHLA2* | . | . | . |  |
| 3 | rs3957558 | 108,179,063 | C | T | 0.03 | 0.02 | intronic | *MYH15* | . | . | . |  |
| 3 | rs11718605 | 108,184,804 | T | C | 0.01 | 0.03 | intronic | *MYH15* | . | . | . |  |
| 3 | rs17630430 | 115,766,285 | C | T | 0.04 | 1.23E-03 | intronic | *LSAMP* | . | . | . |  |
| 3 | rs6782605 | 116,047,619 | T | C | 0.01 | 0.03 | intronic | *LSAMP* | . | . | . |  |
| 3 | rs11925640 | 116,049,751 | C | T | 7.36E-03 | 0.03 | intronic | *LSAMP* | . | . | . |  |
| 3 | rs9869689 | 121,326,676 | T | C | 0.05 | 0.05 | intronic | *FBXO40* | . | . | . |  |
| 3 | rs1568478 | 132,760,427 | C | T | 8.37E-04 | 0.03 | intronic | *TMEM108* | . | . | . |  |
| 3 | rs7650345 | 134,655,446 | G | A | 0.03 | 2.25E-04 | intronic | *EPHB1* | . | . | . |  |
| 3 | rs6788931 | 134,656,624 | A | G | 0.04 | 2.51E-04 | intronic | *EPHB1* | . | . | . |  |
| 3 | rs9817066 | 143,537,964 | A | G | 0.01 | 0.05 | intronic | *SLC9A9* | . | . | . |  |
| 3 | rs12637090 | 143,540,594 | C | A | 0.02 | 0.05 | intronic | *SLC9A9* | . | . | . |  |
| 3 | rs12638011 | 149,837,980 | T | C | 0.02 | 1.27E-03 | ncRNA_intronic | *LOC105374313* | . | . | . |  |
| 3 | rs2029206 | 149,841,837 | A | G | 0.01 | 8.59E-04 | ncRNA_intronic | *LOC105374313* | . | . | . |  |
| 3 | rs6791761 | 149,842,361 | G | A | 1.62E-03 | 8.57E-04 | ncRNA_intronic | *LOC105374313* | . | . | . |  |
| 3 | rs9859538 | 151,090,963 | A | G | 0.03 | 0.01 | intronic | *MED12L;P2RY12* | . | . | . |  |
| 3 | rs4680416 | 157,858,930 | T | C | 4.51E-03 | 5.46E-03 | intronic | *RSRC1* | . | . | . |  |
| 3 | rs6779266 | 157,876,323 | A | C | 5.38E-03 | 5.39E-03 | intronic | *RSRC1* | . | . | . |  |
| 3 | rs698992 | 157,950,607 | G | A | 0.02 | 2.95E-05 | intronic | *RSRC1* | . | . | . |  |
| 3 | rs827123 | 157,950,881 | T | C | 0.02 | 9.81E-05 | intronic | *RSRC1* | . | . | . |  |
| 3 | rs6804180 | 157,958,813 | A | G | 9.53E-03 | 8.42E-05 | intronic | *RSRC1* | . | . | . |  |
| 3 | rs1095633 | 157,961,858 | A | G | 0.02 | 7.96E-05 | intronic | *RSRC1* | . | . | . |  |
| 3 | rs827113 | 157,979,704 | C | T | 0.05 | 4.47E-04 | intronic | *RSRC1* | . | . | . |  |
| 3 | rs2693540 | 158,001,555 | A | G | 0.02 | 3.57E-05 | intronic | *RSRC1* | . | . | . |  |
| 3 | rs1730038 | 158,013,808 | A | G | 0.03 | 3.88E-04 | intronic | *RSRC1* | . | . | . |  |
| 3 | rs827168 | 158,018,849 | C | T | 0.04 | 3.76E-05 | intronic | *RSRC1* | . | . | . |  |
| 3 | rs827117 | 158,038,383 | A | G | 0.03 | 3.81E-04 | intronic | *RSRC1* | . | . | . |  |
| 3 | rs1099106 | 162,978,684 | T | C | 0.04 | 1.02E-03 | ncRNA_intronic | *LINC01192* | . | . | . |  |
| 3 | rs1427106 | 163,001,966 | T | C | 0.04 | 3.95E-03 | ncRNA_intronic | *LINC01192* | . | . | . |  |
| 3 | rs710862 | 163,006,016 | T | C | 0.04 | 4.32E-03 | ncRNA_intronic | *LINC01192* | . | . | . |  |
| 3 | rs843065 | 163,021,072 | C | A | 0.05 | 4.00E-03 | ncRNA_exonic | *LINC01192* | . | . | . |  |
| 3 | rs516741 | 169,966,100 | T | G | 0.04 | 0.05 | intronic | *PRKCI* | . | . | . |  |
| 3 | rs9853317 | 171,917,715 | T | C | 0.02 | 0.04 | intronic | *FNDC3B* | . | . | . |  |
| 3 | rs7633114 | 171,917,877 | T | G | 0.04 | 0.04 | intronic | *FNDC3B* | . | . | . |  |
| 3 | rs13075365 | 173,513,522 | G | T | 0.03 | 4.96E-03 | intronic | *NLGN1* | . | . | . |  |
| 3 | rs13059427 | 173,514,581 | A | G | 0.04 | 7.20E-03 | intronic | *NLGN1* | . | . | . |  |
| 3 | rs10804873 | 178,540,353 | G | T | 0.03 | 6.44E-03 | ncRNA_intronic | *KCNMB2-AS1* | . | . | . |  |
| 3 | rs4549252 | 178,563,170 | G | T | 0.03 | 1.35E-04 | ncRNA_intronic | *KCNMB2-AS1* | . | . | . |  |
| 3 | rs4498019 | 178,563,831 | A | G | 0.05 | 3.22E-04 | ncRNA_intronic | *KCNMB2-AS1* | . | . | . |  |
| 3 | rs11546878 | 183,976,103 | T | C | 0.01 | 0.05 | exonic | *EEF1AKMT4* | . | synonymous SNV | EEF1AKMT4:NM_032331:exon3:c.C508C:p.R170R |  |
| 3 | rs4525855 | 188,261,148 | A | G | 0.04 | 0.04 | intronic | *LPP* | . | . | . |  |
| 3 | rs1963378 | 188,265,563 | T | C | 0.04 | 0.04 | intronic | *LPP* | . | . | . |  |
| 3 | rs4479587 | 188,268,547 | T | C | 0.04 | 0.02 | intronic | *LPP* | . | . | . |  |
| 3 | rs4687163 | 190,341,439 | G | A | 0.05 | 0.03 | intronic | *IL1RAP* | . | . | . |  |
| 3 | rs2050806 | 195,050,247 | C | T | 0.03 | 7.67E-03 | intronic | *ACAP2* | . | . | . |  |
| 3 | rs4677828 | 195,060,129 | C | T | 0.02 | 0.01 | intronic | *ACAP2* | . | . | . |  |
| 4 | rs4144800 | 38,006,916 | G | A | 0.02 | 0.04 | intronic | *TBC1D1* | . | . | . |  |
| 4 | rs6835205 | 38,007,612 | G | A | 0.02 | 0.04 | intronic | *TBC1D1* | . | . | . |  |
| 4 | rs11724057 | 62,686,490 | A | G | 0.04 | 1.65E-03 | intronic | *ADGRL3* | . | . | . |  |
| 4 | rs6551657 | 62,692,132 | G | T | 0.02 | 1.94E-03 | intronic | *ADGRL3* | . | . | . |  |
| 4 | rs1397545 | 62,835,102 | A | G | 7.34E-05 | 0.02 | intronic | *ADGRL3* | . | . | . |  |
| 4 | rs7683738 | 73,311,640 | C | T | 0.04 | 0.03 | intronic | *ADAMTS3* | . | . | . |  |
| 4 | rs10516931 | 94,559,065 | G | A | 0.04 | 0.04 | intronic | *GRID2* | . | . | . |  |
| 4 | rs6532740 | 99,443,938 | A | G | 0.03 | 2.67E-03 | intronic | *TSPAN5* | . | . | . |  |
| 4 | rs9332467 | 99,795,324 | G | A | 9.16E-03 | 5.32E-06 | UTR3 | *EIF4E* | NM_001130678:c.*6855C>T;NM_001331017:c.*6855C>T;NM_001130679:c.*6855C>T;NM_001968:c.*6855C>T | . | . |  |
| 4 | rs7664964 | 99,796,439 | T | C | 0.01 | 1.72E-06 | UTR3 | *EIF4E* | NM_001130678:c.*5740A>G;NM_001331017:c.*5740A>G;NM_001130679:c.*5740A>G;NM_001968:c.*5740A>G | . | . |  |
| 4 | rs17570252 | 99,822,905 | C | T | 7.83E-03 | 2.98E-05 | intronic | *EIF4E* | . | . | . |  |
| 4 | rs17583053 | 99,826,357 | T | C | 0.01 | 4.43E-05 | intronic | *EIF4E* | . | . | . |  |
| 4 | rs11727086 | 99,828,368 | G | A | 8.93E-03 | 4.18E-05 | intronic | *EIF4E* | . | . | . |  |
| 4 | rs6834230 | 99,847,876 | T | C | 6.79E-04 | 8.46E-03 | intronic | *EIF4E* | . | . | . |  |
| 4 | rs13107892 | 99,852,395 | C | T | 0.01 | 5.97E-06 | upstream | *EIF4E* | dist=607 | . | . |  |
| 4 | rs1020624 | 99,944,854 | G | A | 0.03 | 2.44E-06 | intronic | *METAP1* | . | . | . |  |
| 4 | rs10489130 | 99,952,472 | T | G | 2.91E-14 | 0.01 | intronic | *METAP1* | . | . | . | y |
| 4 | rs1037476 | 99,952,607 | C | T | 0.04 | 1.23E-06 | intronic | *METAP1* | . | . | . |  |
| 4 | rs7670154 | 99,955,535 | G | T | 5.32E-12 | 0.03 | intronic | *METAP1* | . | . | . | y |
| 4 | rs1230185 | 99,962,758 | C | T | 0.01 | 2.39E-03 | intronic | *METAP1* | . | . | . |  |
| 4 | rs1230210 | 99,967,691 | T | C | 4.87E-04 | 1.70E-03 | intronic | *METAP1* | . | . | . |  |
| 4 | rs7662987 | 99,991,642 | C | T | 4.89E-04 | 3.73E-03 | downstream | *ADH5* | dist=488 | . | . |  |
| 4 | rs7684986 | 99,991,676 | T | C | 2.57E-04 | 2.72E-03 | downstream | *ADH5* | dist=454 | . | . |  |
| 4 | rs7669660 | 99,992,602 | C | T | 9.40E-04 | 6.69E-04 | UTR3 | *ADH5* | NM_000671:c.*966G>A | . | . |  |
| 4 | rs11547772 | 99,992,793 | C | A | 1.98E-04 | 5.73E-03 | UTR3 | *ADH5* | NM_000671:c.*775G>T | . | . |  |
| 4 | rs1803037 | 99,993,151 | T | C | 2.42E-04 | 5.21E-03 | UTR3 | *ADH5* | NM_000671:c.*417A>G | . | . |  |
| 4 | rs12697 | 99,993,376 | G | A | 8.05E-04 | 6.04E-04 | UTR3 | *ADH5* | NM_000671:c.*192C>T | . | . |  |
| 4 | rs7683802 | 99,995,138 | G | T | 8.14E-05 | 5.31E-03 | intronic | *ADH5* | . | . | . |  |
| 4 | rs4699701 | 99,998,447 | A | G | 1.12E-04 | 5.48E-03 | intronic | *ADH5* | . | . | . |  |
| 4 | rs13119035 | 99,999,130 | G | A | 4.50E-04 | 5.57E-03 | intronic | *ADH5* | . | . | . |  |
| 4 | rs13146416 | 99,999,160 | C | T | 9.07E-05 | 4.36E-03 | intronic | *ADH5* | . | . | . |  |
| 4 | rs13125919 | 99,999,848 | T | C | 2.34E-04 | 5.17E-03 | intronic | *ADH5* | . | . | . |  |
| 4 | rs7683704 | 100,004,226 | T | C | 2.06E-04 | 7.65E-03 | intronic | *ADH5* | . | . | . |  |
| 4 | rs3018048 | 100,005,538 | C | T | 3.92E-05 | 1.84E-06 | intronic | *ADH5* | . | . | . |  |
| 4 | rs1154410 | 100,006,645 | A | G | 3.02E-05 | 6.24E-07 | intronic | *ADH5* | . | . | . |  |
| 4 | rs1154405 | 100,008,750 | G | A | 3.71E-05 | 1.54E-06 | intronic | *ADH5* | . | . | . |  |
| 4 | rs1154400 | 100,010,010 | C | T | 1.02E-04 | 1.34E-04 | ncRNA_exonic | *LOC100507053* | . | . | . |  |
| 4 | rs1311615 | 100,012,261 | G | A | 3.60E-04 | 1.93E-06 | ncRNA_intronic | *LOC100507053* | . | . | . |  |
| 4 | rs1311616 | 100,012,303 | T | G | 6.92E-05 | 1.90E-06 | ncRNA_intronic | *LOC100507053* | . | . | . |  |
| 4 | rs1311617 | 100,012,425 | T | G | 6.92E-05 | 1.24E-06 | ncRNA_intronic | *LOC100507053* | . | . | . |  |
| 4 | rs3018047 | 100,014,510 | T | C | 3.19E-05 | 1.59E-06 | ncRNA_intronic | *LOC100507053* | . | . | . |  |
| 4 | rs2851286 | 100,014,976 | G | A | 3.87E-04 | 1.94E-06 | ncRNA_intronic | *LOC100507053* | . | . | . |  |
| 4 | rs1453874 | 100,022,724 | G | A | 7.73E-05 | 2.22E-06 | ncRNA_intronic | *LOC100507053* | . | . | . |  |
| 4 | rs2602865 | 100,034,841 | T | C | 1.12E-04 | 2.36E-06 | ncRNA_intronic | *LOC100507053* | . | . | . |  |
| 4 | rs2602878 | 100,039,953 | T | G | 4.03E-04 | 3.33E-06 | ncRNA_intronic | *LOC100507053* | . | . | . |  |
| 4 | rs6532795 | 100,042,221 | T | C | 8.32E-07 | 1.96E-07 | ncRNA_intronic | *LOC100507053* | . | . | . |  |
| 4 | rs6532796 | 100,042,242 | A | G | 8.03E-07 | 1.97E-07 | ncRNA_intronic | *LOC100507053* | . | . | . |  |
| 4 | rs2602891 | 100,043,284 | C | T | 1.02E-05 | 1.24E-06 | ncRNA_intronic | *LOC100507053* | . | . | . |  |
| 4 | rs2851253 | 100,043,505 | T | G | 1.29E-05 | 1.64E-06 | ncRNA_intronic | *LOC100507053* | . | . | . |  |
| 4 | rs2924583 | 100,044,640 | T | C | 1.29E-05 | 1.61E-06 | ncRNA_intronic | *LOC100507053* | . | . | . |  |
| 4 | rs1042364 | 100,045,574 | T | C | 1.24E-05 | 1.62E-06 | ncRNA_intronic | *LOC100507053* | . | . | . |  |
| 4 | rs1126673 | 100,045,616 | C | T | 6.31E-07 | 9.59E-08 | exonic | *ADH4* | . | nonsynonymous SNV | ADH4:NM_000670:exon9:c.G1120A:p.V374I,ADH4:NM_001306171:exon10:c.G1177A:p.V393I,ADH4:NM_001306172:exon10:c.G1177A:p.V393I |  |
| 4 | rs2602895 | 100,045,840 | C | T | 1.31E-05 | 1.71E-06 | ncRNA_intronic | *LOC100507053* | . | . | . |  |
| 4 | rs2851248 | 100,045,974 | T | C | 1.31E-05 | 1.70E-06 | ncRNA_intronic | *LOC100507053* | . | . | . |  |
| 4 | rs2851246 | 100,046,104 | T | C | 1.31E-05 | 1.63E-06 | ncRNA_intronic | *LOC100507053* | . | . | . |  |
| 4 | rs2602896 | 100,046,207 | A | G | 1.31E-05 | 1.62E-06 | ncRNA_intronic | *LOC100507053* | . | . | . |  |
| 4 | rs1126672 | 100,047,812 | A | G | 1.38E-05 | 1.99E-06 | exonic | *ADH4* | . | synonymous SNV | ADH4:NM_000670:exon8:c.C1051C:p.L351L,ADH4:NM_001306171:exon9:c.C1108C:p.L370L,ADH4:NM_001306172:exon9:c.C1108C:p.L370L |  |
| 4 | rs1126671 | 100,048,414 | T | C | 6.79E-07 | 1.77E-04 | exonic | *ADH4* | . | nonsynonymous SNV | ADH4:NM_000670:exon7:c.A925G:p.I309V,ADH4:NM_001306171:exon8:c.A982G:p.I328V,ADH4:NM_001306172:exon8:c.A982G:p.I328V |  |
| 4 | rs6837685 | 100,049,145 | G | A | 6.90E-07 | 9.31E-08 | ncRNA_intronic | *LOC100507053* | . | . | . |  |
| 4 | rs1126670 | 100,052,733 | C | A | 6.53E-07 | 6.57E-08 | exonic | *ADH4* | . | synonymous SNV | ADH4:NM_000670:exon6:c.G765T:p.P255P,ADH4:NM_001306171:exon7:c.G822T:p.P274P,ADH4:NM_001306172:exon7:c.G822T:p.P274P |  |
| 4 | rs13129488 | 100,053,025 | T | G | 1.61E-05 | 1.74E-06 | ncRNA_intronic | *LOC100507053* | . | . | . |  |
| 4 | rs29001203 | 100,053,091 | C | T | 1.61E-05 | 1.74E-06 | ncRNA_intronic | *LOC100507053* | . | . | . |  |
| 4 | rs17817359 | 100,054,197 | T | G | 4.77E-05 | 1.59E-06 | ncRNA_intronic | *LOC100507053* | . | . | . |  |
| 4 | rs17217949 | 100,054,312 | G | A | 4.78E-05 | 1.65E-06 | ncRNA_intronic | *LOC100507053* | . | . | . |  |
| 4 | rs6532798 | 100,054,827 | C | T | 2.87E-06 | 8.36E-08 | ncRNA_exonic | *LOC100507053* | . | . | . |  |
| 4 | rs13143133 | 100,054,970 | G | A | 4.58E-05 | 1.47E-06 | ncRNA_intronic | *LOC100507053* | . | . | . |  |
| 4 | rs17218073 | 100,055,739 | A | G | 5.25E-05 | 1.57E-06 | ncRNA_intronic | *LOC100507053* | . | . | . |  |
| 4 | rs10017466 | 100,055,800 | C | T | 3.41E-06 | 7.76E-08 | ncRNA_intronic | *LOC100507053* | . | . | . |  |
| 4 | rs13112176 | 100,055,825 | A | G | 4.59E-05 | 1.59E-06 | ncRNA_intronic | *LOC100507053* | . | . | . |  |
| 4 | rs17218141 | 100,055,908 | C | T | 4.71E-05 | 1.57E-06 | ncRNA_intronic | *LOC100507053* | . | . | . |  |
| 4 | rs17218162 | 100,056,060 | A | G | 4.78E-05 | 1.58E-06 | ncRNA_intronic | *LOC100507053* | . | . | . |  |
| 4 | rs17218239 | 100,057,020 | A | G | 4.46E-05 | 1.58E-06 | ncRNA_intronic | *LOC100507053* | . | . | . |  |
| 4 | rs13133647 | 100,058,727 | T | C | 5.01E-05 | 1.59E-06 | ncRNA_intronic | *LOC100507053* | . | . | . |  |
| 4 | rs7669636 | 100,059,312 | A | G | 3.28E-05 | 1.62E-06 | ncRNA_intronic | *LOC100507053* | . | . | . |  |
| 4 | rs7670060 | 100,059,568 | T | G | 1.14E-04 | 1.77E-06 | ncRNA_intronic | *LOC100507053* | . | . | . |  |
| 4 | rs7670241 | 100,059,619 | T | G | 4.60E-05 | 1.54E-06 | ncRNA_intronic | *LOC100507053* | . | . | . |  |
| 4 | rs4699713 | 100,060,513 | T | C | 4.52E-05 | 1.49E-06 | ncRNA_intronic | *LOC100507053* | . | . | . |  |
| 4 | rs4699714 | 100,060,538 | G | A | 4.52E-05 | 1.51E-06 | ncRNA_intronic | *LOC100507053* | . | . | . |  |
| 4 | rs17817868 | 100,060,623 | T | C | 4.53E-05 | 1.51E-06 | ncRNA_intronic | *LOC100507053* | . | . | . |  |
| 4 | rs17218560 | 100,060,794 | G | A | 4.64E-05 | 1.50E-06 | ncRNA_intronic | *LOC100507053* | . | . | . |  |
| 4 | rs17817958 | 100,061,245 | T | G | 4.64E-05 | 1.55E-06 | ncRNA_intronic | *LOC100507053* | . | . | . |  |
| 4 | rs13110176 | 100,062,430 | G | A | 4.77E-05 | 1.42E-06 | ncRNA_intronic | *LOC100507053* | . | . | . |  |
| 4 | rs13110764 | 100,062,466 | A | G | 4.77E-05 | 1.40E-06 | ncRNA_intronic | *LOC100507053* | . | . | . |  |
| 4 | rs13138294 | 100,062,675 | G | T | 4.77E-05 | 1.40E-06 | ncRNA_intronic | *LOC100507053* | . | . | . |  |
| 4 | rs4699716 | 100,066,125 | A | G | 3.44E-04 | 1.77E-07 | ncRNA_intronic | *LOC100507053* | . | . | . |  |
| 4 | rs4699717 | 100,066,137 | T | C | 3.44E-04 | 1.73E-07 | ncRNA_intronic | *LOC100507053* | . | . | . |  |
| 4 | rs4148883 | 100,066,332 | T | C | 1.11E-03 | 0.01 | ncRNA_intronic | *LOC100507053* | . | . | . |  |
| 4 | rs7664780 | 100,076,309 | G | A | 2.65E-03 | 7.81E-06 | ncRNA_exonic | *LOC100507053* | . | . | . |  |
| 4 | rs6814114 | 100,122,819 | T | G | 9.58E-03 | 1.82E-04 | ncRNA_intronic | *LOC100507053* | . | . | . |  |
| 4 | rs2051428 | 100,123,186 | G | A | 3.83E-11 | 0.01 | ncRNA_intronic | *LOC100507053* | . | . | . |  |
| 4 | rs17219626 | 100,126,896 | G | A | 8.67E-03 | 1.77E-04 | ncRNA_intronic | *LOC100507053* | . | . | . |  |
| 4 | rs9995799 | 100,138,538 | A | C | 1.90E-11 | 0.01 | ncRNA_intronic | *LOC100507053* | . | . | . |  |
| 4 | rs4699738 | 100,175,530 | A | G | 1.29E-10 | 8.60E-03 | ncRNA_intronic | *LOC100507053* | . | . | . |  |
| 4 | rs2066702 | 100,229,017 | A | G | 4.69E-24 | 5.12E-04 | exonic | *ADH1B* | . | synonymous SNV | ADH1B:NM_000668:exon9:c.C1108C:p.R370R,ADH1B:NM_001286650:exon10:c.C988C:p.R330R | y |
| 4 | rs2298753 | 100,257,907 | C | T | 8.00E-04 | 1.02E-13 | UTR3 | *ADH1C* | NM_000669:c.*2G>A | . | . |  |
| 4 | rs1662033 | 100,258,381 | G | T | 0.01 | 2.63E-06 | intronic | *ADH1C* | . | . | . |  |
| 4 | rs1693476 | 100,260,274 | C | T | 1.79E-04 | 7.73E-19 | intronic | *ADH1C* | . | . | . |  |
| 4 | rs698 | 100,260,789 | C | T | 1.80E-04 | 1.02E-18 | exonic | *ADH1C* | . | unknown | UNKNOWN |  |
| 4 | rs904095 | 100,263,383 | G | A | 1.67E-04 | 9.85E-19 | intronic | *ADH1C* | . | . | . |  |
| 4 | rs904096 | 100,263,584 | G | T | 1.64E-04 | 1.09E-18 | intronic | *ADH1C* | . | . | . |  |
| 4 | rs1789911 | 100,263,778 | C | T | 1.54E-04 | 1.09E-18 | intronic | *ADH1C* | . | . | . |  |
| 4 | rs283411 | 100,265,957 | A | C | 8.34E-03 | 7.42E-05 | intronic | *ADH1C* | . | . | . |  |
| 4 | rs1662051 | 100,267,239 | A | C | 1.23E-04 | 9.66E-19 | intronic | *ADH1C* | . | . | . |  |
| 4 | rs1789920 | 100,268,856 | C | A | 0.01 | 8.23E-06 | intronic | *ADH1C* | . | . | . |  |
| 4 | rs1789921 | 100,269,202 | A | G | 0.01 | 7.94E-06 | intronic | *ADH1C* | . | . | . |  |
| 4 | rs1789924 | 100,274,286 | T | C | 1.37E-04 | 8.78E-18 | upstream | *ADH1C* | dist=383 | . | . |  |
| 4 | rs17586163 | 100,274,571 | C | T | 7.86E-04 | 1.07E-13 | upstream | *ADH1C* | dist=668 | . | . |  |
| 4 | rs11499823 | 100,274,749 | G | A | 8.00E-04 | 9.25E-14 | upstream | *ADH1C* | dist=846 | . | . |  |
| 4 | rs11097669 | 100,486,295 | G | A | 0.02 | 6.48E-03 | intronic | *MTTP* | . | . | . |  |
| 4 | rs17029142 | 100,486,651 | A | C | 0.02 | 7.66E-03 | intronic | *MTTP* | . | . | . |  |
| 4 | rs17208914 | 102,854,390 | G | A | 0.02 | 3.06E-05 | intronic | *BANK1* | . | . | . |  |
| 4 | rs2298752 | 103,177,071 | A | G | 0.01 | 0.02 | intronic | *SLC39A8* | . | . | . |  |
| 4 | rs7662378 | 103,179,788 | A | G | 6.86E-03 | 0.02 | intronic | *SLC39A8* | . | . | . |  |
| 4 | rs7676998 | 109,009,696 | T | C | 0.03 | 8.40E-03 | intronic | *LEF1* | . | . | . |  |
| 4 | rs898518 | 109,016,824 | C | A | 0.02 | 9.52E-03 | intronic | *LEF1* | . | . | . |  |
| 4 | rs7698367 | 109,030,482 | C | T | 0.04 | 8.19E-03 | intronic | *LEF1* | . | . | . |  |
| 4 | rs6854803 | 109,095,552 | A | G | 0.01 | 0.04 | ncRNA_intronic | *LEF1-AS1* | . | . | . |  |
| 4 | rs881878 | 110,836,048 | A | G | 0.03 | 0.05 | intronic | *EGF* | . | . | . |  |
| 4 | rs2595110 | 111,545,323 | G | A | 0.04 | 0.03 | intronic | *PITX2* | . | . | . |  |
| 4 | rs11723116 | 118,281,370 | A | G | 0.05 | 2.47E-04 | ncRNA_intronic | *LINC02262* | . | . | . |  |
| 4 | rs3805236 | 144,357,737 | A | G | 0.04 | 0.03 | intronic | *GAB1* | . | . | . |  |
| 4 | rs12642131 | 173,520,376 | G | A | 0.02 | 0.05 | intronic | *GALNTL6* | . | . | . |  |
| 4 | rs10007836 | 188,297,817 | T | G | 0.01 | 6.23E-04 | ncRNA_intronic | *LOC339975* | . | . | . |  |
| 5 | rs12513430 | 20,459,487 | G | A | 0.04 | 0.04 | intronic | *CDH18* | . | . | . |  |
| 5 | rs2457026 | 20,514,987 | A | G | 0.01 | 0.03 | intronic | *CDH18* | . | . | . |  |
| 5 | rs17234617 | 20,520,322 | A | G | 0.01 | 0.02 | intronic | *CDH18* | . | . | . |  |
| 5 | rs2940570 | 22,385,221 | C | T | 2.09E-03 | 0.04 | intronic | *CDH12* | . | . | . |  |
| 5 | rs7706830 | 33,228,911 | A | G | 0.03 | 0.03 | upstream | *LINC02160* | dist=930 | . | . |  |
| 5 | rs4498262 | 33,229,174 | G | T | 0.03 | 0.02 | upstream | *LINC02160* | dist=667 | . | . |  |
| 5 | rs12054719 | 38,519,550 | C | T | 0.04 | 0.03 | intronic | *LIFR* | . | . | . |  |
| 5 | rs10072745 | 60,083,275 | C | A | 0.04 | 1.02E-03 | intronic | *ELOVL7* | . | . | . |  |
| 5 | rs4235481 | 60,093,387 | C | T | 0.04 | 5.87E-04 | intronic | *ELOVL7* | . | . | . |  |
| 5 | rs7726671 | 60,190,594 | G | A | 0.03 | 4.79E-03 | intronic | *ERCC8* | . | . | . |  |
| 5 | rs4647113 | 60,196,432 | T | C | 0.01 | 4.43E-03 | intronic | *ERCC8* | . | . | . |  |
| 5 | rs34635 | 60,513,501 | G | A | 0.02 | 0.04 | ncRNA_intronic | *SMIM15-AS1* | . | . | . |  |
| 5 | rs12657371 | 78,728,025 | C | T | 8.41E-03 | 0.02 | intronic | *HOMER1* | . | . | . |  |
| 5 | rs7719054 | 78,728,923 | C | T | 0.02 | 0.02 | intronic | *HOMER1* | . | . | . |  |
| 5 | rs6863905 | 79,511,472 | G | T | 0.02 | 0.02 | intronic | *SERINC5* | . | . | . |  |
| 5 | rs7736480 | 95,318,871 | C | T | 0.04 | 0.05 | ncRNA_intronic | *LOC101929710* | . | . | . |  |
| 5 | rs6893788 | 95,335,086 | T | C | 0.04 | 0.04 | ncRNA_intronic | *LOC101929710* | . | . | . |  |
| 5 | rs2400796 | 101,775,394 | T | C | 0.03 | 0.02 | intronic | *SLCO6A1* | . | . | . |  |
| 5 | rs12519591 | 106,944,011 | A | C | 0.02 | 0.02 | intronic | *EFNA5* | . | . | . |  |
| 5 | rs17164127 | 127,773,702 | T | C | 1.44E-05 | 0.02 | intronic | *FBN2* | . | . | . |  |
| 5 | rs13161129 | 132,830,294 | A | G | 0.04 | 0.04 | intronic | *FSTL4* | . | . | . |  |
| 5 | rs889012 | 135,554,716 | T | G | 0.04 | 0.02 | intronic | *TRPC7* | . | . | . |  |
| 5 | rs3734123 | 135,561,133 | T | C | 0.03 | 0.02 | intronic | *TRPC7* | . | . | . |  |
| 5 | rs13162836 | 153,586,443 | G | A | 0.02 | 0.04 | intronic | *GALNT10* | . | . | . |  |
| 5 | rs10059636 | 153,620,581 | G | T | 0.03 | 1.17E-03 | intronic | *GALNT10* | . | . | . |  |
| 5 | rs1541663 | 153,620,638 | C | T | 0.04 | 3.64E-03 | intronic | *GALNT10* | . | . | . |  |
| 5 | rs7732135 | 167,138,060 | A | G | 0.03 | 0.01 | intronic | *TENM2* | . | . | . |  |
| 5 | rs9313396 | 167,499,832 | G | T | 0.04 | 0.02 | intronic | *TENM2* | . | . | . |  |
| 5 | rs17633196 | 167,834,810 | C | T | 0.03 | 0.01 | intronic | *WWC1* | . | . | . |  |
| 5 | rs736077 | 173,134,162 | C | T | 0.02 | 0.02 | downstream | *LINC01484* | dist=440 | . | . |  |
| 5 | rs17064225 | 174,417,049 | T | C | 0.04 | 0.04 | ncRNA_intronic | *LINC01951* | . | . | . |  |
| 5 | rs7707147 | 177,652,392 | C | T | 0.03 | 2.49E-04 | exonic | *PHYKPL* | . | synonymous SNV | PHYKPL:NM_001278346:exon4:c.A254A:p.H85H,PHYKPL:NM_153373:exon4:c.A377A:p.H126H | y |
| 5 | rs2913756 | 177,725,276 | C | T | 0.05 | 0.03 | intronic | *COL23A1* | . | . | . |  |
| 6 | rs9504350 | 5,136,097 | C | T | 7.55E-03 | 0.04 | ncRNA_intronic | *LYRM4-AS1* | . | . | . |  |
| 6 | rs573082 | 11,569,747 | A | G | 0.04 | 1.63E-04 | intronic | *TMEM170B* | . | . | . |  |
| 6 | rs490466 | 11,577,085 | T | C | 0.04 | 1.66E-04 | UTR3 | *TMEM170B* | NM_001100829:c.*1291T>C | . | . |  |
| 6 | rs16877977 | 16,386,345 | A | G | 0.05 | 0.04 | intronic | *ATXN1* | . | . | . |  |
| 6 | rs9477107 | 16,386,953 | G | A | 0.04 | 0.04 | intronic | *ATXN1* | . | . | . |  |
| 6 | rs4576240 | 24,596,478 | T | G | 0.05 | 7.56E-03 | exonic | *KIAA0319* | . | nonsynonymous SNV | KIAA0319:NM_001168376:exon2:c.A289C:p.T97P,KIAA0319:NM_001350404:exon2:c.A406C:p.T136P,KIAA0319:NM_001350406:exon2:c.A289C:p.T97P,KIAA0319:NM_001168375:exon3:c.A424C:p.T142P,KIAA0319:NM_001168377:exon3:c.A424C:p.T142P,KIAA0319:NM_001350403:exon3:c.A424C:p.T142P,KIAA0319:NM_001350405:exon3:c.A424C:p.T142P,KIAA0319:NM_001350407:exon3:c.A424C:p.T142P,KIAA0319:NM_001350408:exon3:c.A424C:p.T142P,KIAA0319:NM_014809:exon3:c.A424C:p.T142P,KIAA0319:NM_001168374:exon4:c.A397C:p.T133P |  |
| 6 | rs4959053 | 31,099,577 | A | G | 0.03 | 5.46E-03 | intronic | *PSORS1C1* | . | . | . |  |
| 6 | rs1049853 | 31,236,900 | A | G | 0.04 | 0.05 | UTR3 | *HLA-C* | NM_002117:c.*46T>C;NM_001243042:c.*46T>C | . | . |  |
| 6 | rs2395471 | 31,240,692 | G | A | 5.22E-03 | 6.93E-04 | upstream | *HLA-C* | dist=779 | . | . |  |
| 6 | rs9267247 | 31,455,834 | A | C | 0.01 | 6.45E-03 | ncRNA_intronic | *MICB-DT* | . | . | . |  |
| 6 | rs2904783 | 31,459,972 | C | T | 0.03 | 0.02 | ncRNA_intronic | *MICB-DT* | . | . | . |  |
| 6 | rs2239705 | 31,513,402 | A | G | 0.03 | 0.01 | ncRNA_intronic | *ATP6V1G2-DDX39B* | . | . | . |  |
| 6 | rs11965323 | 31,629,638 | T | G | 5.95E-03 | 6.96E-03 | UTR3 | *GPANK1* | NM_033177:c.*405A>C;NM_001199237:c.*405A>C;NM_001199239:c.*405A>C;NM_001199240:c.*405A>C;NM_001199238:c.*405A>C | . | . | y |
| 6 | rs2295667 | 31,630,172 | T | G | 6.10E-03 | 6.97E-03 | exonic | *GPANK1* | . | synonymous SNV | GPANK1:NM_033177:exon3:c.C942C:p.T314T,GPANK1:NM_001199237:exon4:c.C942C:p.T314T,GPANK1:NM_001199238:exon4:c.C942C:p.T314T,GPANK1:NM_001199239:exon4:c.C942C:p.T314T,GPANK1:NM_001199240:exon4:c.C942C:p.T314T | y |
| 6 | rs2254083 | 31,639,741 | T | C | 6.84E-03 | 7.00E-03 | exonic | *LY6G5B* | . | synonymous SNV | LY6G5B:NM_021221:exon3:c.C288C:p.A96A | y |
| 6 | rs11969759 | 32,021,130 | T | C | 0.02 | 0.04 | intronic | *TNXB* | . | . | . |  |
| 6 | rs3131283 | 32,119,898 | T | C | 0.02 | 0.05 | UTR5 | *PRRT1* | NM_001363780:c.-19A>G | . | . |  |
| 6 | rs3134943 | 32,147,761 | T | C | 0.03 | 0.04 | intronic | *RNF5* | . | . | . |  |
| 6 | rs11759011 | 33,650,812 | A | G | 0.04 | 1.23E-04 | intronic | *ITPR3* | . | . | . |  |
| 6 | rs2296742 | 33,659,793 | A | G | 7.19E-04 | 3.35E-03 | intronic | *ITPR3* | . | . | . |  |
| 6 | rs4711338 | 33,660,188 | C | T | 7.15E-04 | 3.36E-03 | intronic | *ITPR3* | . | . | . |  |
| 6 | rs626156 | 33,670,482 | G | A | 0.03 | 0.04 | intronic | *UQCC2* | . | . | . |  |
| 6 | rs542441 | 33,675,851 | C | A | 0.03 | 0.04 | intronic | *UQCC2* | . | . | . |  |
| 6 | rs2966 | 33,689,520 | T | C | 1.12E-03 | 3.24E-03 | UTR3 | *IP6K3* | NM_054111:c.*977A>G;NM_001142883:c.*977A>G | . | . |  |
| 6 | rs4713668 | 33,690,796 | T | C | 5.51E-03 | 3.30E-03 | exonic | *IP6K3* | . | synonymous SNV | IP6K3:NM_054111:exon6:c.G934G:p.V312V,IP6K3:NM_001142883:exon7:c.G934G:p.V312V |  |
| 6 | rs3828783 | 33,767,727 | A | G | 3.71E-03 | 4.95E-04 | intronic | *MLN* | . | . | . |  |
| 6 | rs9471969 | 42,906,384 | T | G | 0.05 | 8.07E-04 | exonic | *CNPY3* | . | synonymous SNV | CNPY3:NM_001318845:exon5:c.G425G:p.S142S,CNPY3:NM_006586:exon6:c.G692G:p.S231S,CNPY3:NM_001318842:exon7:c.G791G:p.S264S |  |
| 6 | rs9462852 | 42,907,327 | T | C | 8.99E-03 | 6.55E-03 | intronic | *CNPY3-GNMT* | . | . | . |  |
| 6 | rs4987173 | 42,931,224 | A | G | 6.39E-03 | 0.04 | intronic | *CNPY3-GNMT;GNMT* | . | . | . |  |
| 6 | rs1129187 | 42,932,200 | T | G | 6.13E-03 | 0.04 | exonic | *PEX6* | . | synonymous SNV | PEX6:NM_000287:exon17:c.C2816C:p.P939P,PEX6:NM_001316313:exon17:c.C2552C:p.P851P |  |
| 6 | rs9462859 | 42,946,943 | A | G | 6.53E-03 | 0.04 | upstream | *PEX6* | dist=24 | . | . |  |
| 6 | rs6459153 | 56,197,525 | T | C | 0.02 | 0.02 | intronic | *COL21A1* | . | . | . |  |
| 6 | rs517392 | 64,623,590 | T | C | 0.02 | 1.42E-03 | intronic | *EYS* | . | . | . |  |
| 6 | rs452975 | 64,625,328 | T | C | 0.03 | 1.70E-03 | intronic | *EYS* | . | . | . |  |
| 6 | rs1336764 | 75,324,453 | A | C | 0.04 | 0.04 | ncRNA_intronic | *LOC101928516* | . | . | . |  |
| 6 | rs12660483 | 88,250,620 | G | A | 0.03 | 5.58E-03 | intronic | *RARS2* | . | . | . |  |
| 6 | rs2787897 | 88,526,850 | T | G | 0.03 | 4.35E-03 | ncRNA_intronic | *LOC101928911* | . | . | . |  |
| 6 | rs9111 | 90,981,653 | C | T | 0.03 | 0.02 | UTR5 | *BACH2* | NM_021813:c.-263090G>A | . | . |  |
| 6 | rs1386274 | 93,961,571 | T | C | 0.01 | 0.05 | intronic | *EPHA7* | . | . | . |  |
| 6 | rs404201 | 93,961,892 | C | T | 0.02 | 0.04 | intronic | *EPHA7* | . | . | . |  |
| 6 | rs419641 | 93,963,135 | C | A | 0.02 | 0.04 | intronic | *EPHA7* | . | . | . |  |
| 6 | rs345737 | 93,964,187 | T | C | 0.01 | 0.05 | intronic | *EPHA7* | . | . | . |  |
| 6 | rs6571202 | 105,182,644 | G | T | 0.02 | 0.04 | intronic | *HACE1* | . | . | . |  |
| 6 | rs2486135 | 105,282,263 | T | C | 0.03 | 0.04 | intronic | *HACE1* | . | . | . |  |
| 6 | rs2486143 | 105,308,593 | G | A | 0.05 | 0.03 | upstream | *HACE1* | dist=799 | . | . |  |
| 6 | rs2253957 | 105,771,520 | T | C | 0.04 | 1.39E-03 | intronic | *PREP* | . | . | . |  |
| 6 | rs594664 | 124,442,286 | T | C | 1.89E-03 | 0.03 | intronic | *NKAIN2* | . | . | . |  |
| 6 | rs7765677 | 143,259,563 | G | A | 0.04 | 1.14E-03 | intronic | *HIVEP2* | . | . | . |  |
| 6 | rs9479126 | 152,146,580 | C | T | 0.03 | 0.04 | intronic | *ESR1* | . | . | . | y |
| 6 | rs9458255 | 161,863,770 | A | G | 0.02 | 0.01 | intronic | *PRKN* | . | . | . |  |
| 6 | rs9458257 | 161,863,950 | T | C | 0.02 | 0.02 | intronic | *PRKN* | . | . | . |  |
| 6 | rs4073926 | 170,099,398 | C | T | 0.05 | 4.15E-04 | intronic | *WDR27* | . | . | . |  |
| 6 | rs4716398 | 170,176,407 | A | G | 0.04 | 6.65E-04 | intronic | *ERMARD* | . | . | . |  |
| 6 | rs4716399 | 170,176,647 | T | C | 0.04 | 6.09E-04 | exonic | *ERMARD* | . | synonymous SNV | ERMARD:NM_001278532:exon15:c.C1239C:p.I413I,ERMARD:NM_018341:exon16:c.C1617C:p.I539I |  |
| 7 | rs7808785 | 7,805,729 | A | G | 0.04 | 0.03 | intronic | *UMAD1* | . | . | . |  |
| 7 | rs7811417 | 21,534,152 | T | C | 0.02 | 0.02 | intronic | *SP4* | . | . | . |  |
| 7 | rs10499538 | 21,698,100 | T | C | 0.04 | 0.04 | intronic | *DNAH11* | . | . | . |  |
| 7 | rs17473365 | 33,192,800 | C | T | 0.05 | 4.94E-03 | intronic | *BBS9* | . | . | . |  |
| 7 | rs2109525 | 33,411,267 | G | T | 0.05 | 8.00E-03 | intronic | *BBS9* | . | . | . |  |
| 7 | rs1732001 | 37,475,424 | T | C | 0.05 | 0.02 | intronic | *ELMO1* | . | . | . |  |
| 7 | rs4457209 | 71,639,338 | G | A | 0.04 | 1.22E-04 | intronic | *CALN1* | . | . | . |  |
| 7 | rs477387 | 71,732,674 | C | T | 0.02 | 2.05E-03 | intronic | *CALN1* | . | . | . |  |
| 7 | rs485865 | 71,734,051 | A | G | 0.02 | 2.69E-03 | intronic | *CALN1* | . | . | . |  |
| 7 | rs1468163 | 71,740,458 | A | G | 0.04 | 1.35E-05 | intronic | *CALN1* | . | . | . |  |
| 7 | rs501383 | 71,744,896 | A | G | 0.03 | 2.62E-03 | intronic | *CALN1* | . | . | . |  |
| 7 | rs2944829 | 71,786,721 | A | G | 0.03 | 6.94E-06 | intronic | *CALN1* | . | . | . |  |
| 7 | rs11982081 | 71,788,267 | A | C | 0.03 | 1.66E-05 | intronic | *CALN1* | . | . | . |  |
| 7 | rs2944825 | 71,793,386 | C | T | 0.04 | 1.16E-05 | intronic | *CALN1* | . | . | . |  |
| 7 | rs2944822 | 71,795,592 | T | C | 0.02 | 1.54E-05 | intronic | *CALN1* | . | . | . |  |
| 7 | rs3807716 | 77,759,391 | A | G | 0.05 | 8.76E-03 | intronic | *MAGI2* | . | . | . |  |
| 7 | rs10487060 | 86,590,353 | A | C | 0.04 | 1.26E-03 | intronic | *ELAPOR2* | . | . | . |  |
| 7 | rs17164370 | 88,418,900 | C | T | 0.02 | 5.52E-03 | intronic | *ZNF804B* | . | . | . |  |
| 7 | rs17303650 | 93,552,275 | A | G | 0.02 | 0.04 | intronic | *GNG11* | . | . | . |  |
| 7 | rs7803157 | 122,004,908 | C | T | 9.19E-03 | 0.04 | intronic | *CADPS2* | . | . | . |  |
| 7 | rs12670788 | 131,195,712 | A | G | 7.70E-03 | 0.01 | exonic | *PODXL* | . | synonymous SNV | PODXL:NM_001018111:exon2:c.C581C:p.S194S,PODXL:NM_005397:exon2:c.C581C:p.S194S |  |
| 7 | rs3735035 | 131,195,959 | T | C | 7.86E-03 | 0.01 | exonic | *PODXL* | . | synonymous SNV | PODXL:NM_001018111:exon2:c.G334G:p.G112G,PODXL:NM_005397:exon2:c.G334G:p.G112G |  |
| 7 | rs1593304 | 131,619,847 | G | A | 7.90E-03 | 1.43E-03 | ncRNA_intronic | *LOC101928782* | . | . | . |  |
| 7 | rs10269360 | 135,147,425 | C | A | 0.02 | 0.05 | intronic | *CNOT4* | . | . | . |  |
| 7 | rs4637730 | 140,923,630 | T | C | 0.01 | 0.03 | intronic | *TMEM178B* | . | . | . |  |
| 7 | rs10952500 | 141,130,890 | A | G | 3.38E-03 | 8.84E-03 | intronic | *TMEM178B* | . | . | . |  |
| 8 | rs2081279 | 3,115,165 | A | G | 0.05 | 0.01 | intronic | *CSMD1* | . | . | . |  |
| 8 | rs2740914 | 3,895,072 | T | C | 0.02 | 0.02 | intronic | *CSMD1* | . | . | . |  |
| 8 | rs1714701 | 4,125,066 | A | G | 0.04 | 2.28E-03 | intronic | *CSMD1* | . | . | . |  |
| 8 | rs4840427 | 9,435,825 | C | T | 0.05 | 0.04 | intronic | *TNKS* | . | . | . |  |
| 8 | rs7814014 | 9,436,921 | G | A | 0.03 | 4.45E-03 | intronic | *TNKS* | . | . | . |  |
| 8 | rs7834823 | 9,437,352 | A | C | 0.02 | 8.33E-03 | intronic | *TNKS* | . | . | . |  |
| 8 | rs4841179 | 9,460,449 | G | A | 0.02 | 1.51E-03 | intronic | *TNKS* | . | . | . |  |
| 8 | rs7832096 | 9,462,583 | C | T | 0.02 | 1.48E-03 | intronic | *TNKS* | . | . | . |  |
| 8 | rs10903314 | 9,467,106 | T | C | 0.02 | 1.40E-03 | intronic | *TNKS* | . | . | . |  |
| 8 | rs4398920 | 9,469,576 | T | C | 0.02 | 1.61E-03 | intronic | *TNKS* | . | . | . |  |
| 8 | rs4240626 | 9,471,060 | G | A | 0.02 | 1.87E-03 | intronic | *TNKS* | . | . | . |  |
| 8 | rs11249930 | 9,472,445 | G | A | 9.11E-03 | 5.46E-04 | intronic | *TNKS* | . | . | . |  |
| 8 | rs4457339 | 9,479,149 | G | A | 0.01 | 8.03E-04 | intronic | *TNKS* | . | . | . |  |
| 8 | rs11784181 | 9,479,926 | C | T | 6.75E-03 | 6.58E-04 | intronic | *TNKS* | . | . | . |  |
| 8 | rs12674762 | 9,484,126 | C | T | 0.02 | 3.03E-03 | intronic | *TNKS* | . | . | . |  |
| 8 | rs13273033 | 9,540,693 | G | A | 0.01 | 1.17E-03 | intronic | *TNKS* | . | . | . |  |
| 8 | rs6990300 | 9,547,861 | G | A | 0.03 | 0.05 | intronic | *TNKS* | . | . | . |  |
| 8 | rs4841196 | 9,555,092 | A | G | 0.03 | 0.04 | intronic | *TNKS* | . | . | . |  |
| 8 | rs7462910 | 9,579,986 | T | C | 7.63E-03 | 3.28E-03 | intronic | *TNKS* | . | . | . |  |
| 8 | rs11779335 | 9,587,854 | C | T | 6.45E-03 | 2.43E-03 | intronic | *TNKS* | . | . | . |  |
| 8 | rs10098474 | 9,911,617 | T | C | 0.03 | 0.01 | upstream | *MSRA* | dist=185 | . | . |  |
| 8 | rs7832431 | 9,947,661 | C | A | 0.04 | 4.57E-04 | intronic | *MSRA* | . | . | . |  |
| 8 | rs13257313 | 10,162,381 | C | A | 6.37E-03 | 0.05 | intronic | *MSRA* | . | . | . |  |
| 8 | rs11775348 | 10,176,177 | C | T | 0.03 | 0.04 | intronic | *MSRA* | . | . | . |  |
| 8 | rs11249990 | 10,182,144 | C | T | 5.08E-03 | 0.02 | intronic | *MSRA* | . | . | . |  |
| 8 | rs4841326 | 10,204,562 | T | C | 9.03E-03 | 1.14E-03 | intronic | *MSRA* | . | . | . |  |
| 8 | rs7842777 | 10,335,032 | G | T | 0.05 | 0.01 | ncRNA_intronic | *LINCR-0001* | . | . | . |  |
| 8 | rs7845103 | 17,084,866 | C | A | 0.05 | 0.03 | UTR3 | *CNOT7* | NM_001322091:c.*3363G>T;NM_001322097:c.*3363G>T;NM_001322090:c.*3363G>T;NM_013354:c.*3363G>T;NM_001322092:c.*3363G>T;NM_001322095:c.*3363G>T;NM_001322093:c.*3363G>T;NM_001322096:c.*3363G>T | . | . |  |
| 8 | rs3793428 | 17,144,020 | C | A | 0.01 | 0.03 | intronic | *VPS37A* | . | . | . |  |
| 8 | rs2942194 | 23,423,669 | G | A | 0.01 | 2.64E-06 | exonic | *SLC25A37* | . | synonymous SNV | SLC25A37:NM_016612:exon2:c.A259A:p.I87I,SLC25A37:NM_001317813:exon3:c.A43A:p.I15I,SLC25A37:NM_001317814:exon3:c.A43A:p.I15I |  |
| 8 | rs6998793 | 37,696,874 | T | C | 0.01 | 0.04 | intronic | *ADGRA2* | . | . | . |  |
| 8 | rs4647907 | 38,287,555 | T | C | 0.04 | 0.04 | intronic | *FGFR1* | . | . | . |  |
| 8 | rs10101096 | 38,292,147 | C | A | 0.01 | 0.04 | intronic | *FGFR1* | . | . | . |  |
| 8 | rs13262219 | 40,561,154 | A | G | 3.50E-03 | 8.29E-03 | intronic | *ZMAT4* | . | . | . |  |
| 8 | rs4873570 | 52,579,316 | A | C | 1.43E-03 | 2.18E-03 | intronic | *PXDNL* | . | . | . |  |
| 8 | rs4382468 | 52,590,541 | A | G | 3.18E-03 | 2.00E-03 | intronic | *PXDNL* | . | . | . |  |
| 8 | rs13261717 | 52,641,007 | C | A | 0.01 | 3.40E-03 | intronic | *PXDNL* | . | . | . |  |
| 8 | rs11780891 | 52,674,627 | T | C | 0.01 | 5.80E-03 | intronic | *PXDNL* | . | . | . |  |
| 8 | rs4534123 | 52,677,857 | C | A | 1.82E-03 | 0.05 | intronic | *PXDNL* | . | . | . |  |
| 8 | rs11778849 | 52,708,254 | T | C | 0.02 | 5.98E-03 | intronic | *PXDNL* | . | . | . |  |
| 8 | rs763550 | 52,715,465 | T | C | 0.02 | 4.49E-04 | intronic | *PXDNL* | . | . | . |  |
| 8 | rs41453644 | 52,733,544 | C | T | 0.02 | 2.29E-04 | intronic | *PCMTD1* | . | . | . |  |
| 8 | rs16916856 | 52,739,150 | C | T | 5.35E-03 | 2.08E-04 | intronic | *PCMTD1* | . | . | . |  |
| 8 | rs10504130 | 52,757,093 | A | G | 0.03 | 4.56E-04 | intronic | *PCMTD1* | . | . | . |  |
| 8 | rs7462267 | 56,150,369 | A | G | 0.03 | 0.01 | intronic | *XKR4* | . | . | . |  |
| 8 | rs13438934 | 56,155,049 | G | A | 0.02 | 0.01 | intronic | *XKR4* | . | . | . |  |
| 8 | rs4368953 | 56,175,875 | G | A | 0.03 | 7.88E-03 | intronic | *XKR4* | . | . | . |  |
| 8 | rs4562295 | 56,176,565 | T | C | 0.03 | 7.47E-03 | intronic | *XKR4* | . | . | . |  |
| 8 | rs4738044 | 56,201,122 | C | T | 0.05 | 6.79E-03 | intronic | *XKR4* | . | . | . |  |
| 8 | rs4737416 | 56,661,064 | C | A | 7.95E-03 | 0.03 | intronic | *TMEM68* | . | . | . |  |
| 8 | rs1440747 | 57,417,924 | T | G | 0.05 | 3.37E-08 | ncRNA_intronic | *LOC101929415* | . | . | . |  |
| 8 | rs16928640 | 63,190,745 | A | G | 0.02 | 0.01 | intronic | *NKAIN3* | . | . | . |  |
| 8 | rs35352031 | 67,344,005 | T | G | 0.01 | 0.05 | upstream | *ADHFE1* | dist=734 | . | . |  |
| 8 | rs3750228 | 70,980,738 | C | T | 0.04 | 8.41E-03 | exonic | *PRDM14* | . | synonymous SNV | PRDM14:NM_024504:exon3:c.A730A:p.K244K |  |
| 8 | rs4147529 | 104,045,884 | A | G | 0.04 | 0.03 | intronic | *ATP6V1C1* | . | . | . |  |
| 8 | rs1375960 | 106,624,958 | A | G | 0.05 | 0.04 | intronic | *ZFPM2* | . | . | . |  |
| 8 | rs2938303 | 107,299,053 | T | C | 0.04 | 7.57E-04 | intronic | *OXR1* | . | . | . |  |
| 8 | rs3019308 | 107,300,650 | G | A | 0.04 | 1.27E-03 | intronic | *OXR1* | . | . | . |  |
| 8 | rs6993552 | 110,393,142 | C | T | 0.04 | 0.04 | intronic | *PKHD1L1* | . | . | . |  |
| 8 | rs921959 | 110,411,338 | C | T | 0.03 | 0.05 | intronic | *PKHD1L1* | . | . | . |  |
| 8 | rs12679728 | 110,418,461 | T | G | 0.02 | 0.04 | intronic | *PKHD1L1* | . | . | . |  |
| 8 | rs13254245 | 110,420,384 | C | T | 0.02 | 0.04 | exonic | *PKHD1L1* | . | synonymous SNV | PKHD1L1:NM_177531:exon18:c.T1920T:p.N640N |  |
| 8 | rs13252234 | 110,420,429 | G | A | 0.02 | 0.04 | exonic | *PKHD1L1* | . | synonymous SNV | PKHD1L1:NM_177531:exon18:c.A1965A:p.E655E |  |
| 8 | rs9297425 | 110,421,113 | T | C | 0.02 | 0.04 | intronic | *PKHD1L1* | . | . | . |  |
| 8 | rs12681579 | 110,421,793 | T | G | 0.02 | 0.04 | intronic | *PKHD1L1* | . | . | . |  |
| 8 | rs2349761 | 110,427,024 | C | A | 0.03 | 0.04 | intronic | *PKHD1L1* | . | . | . |  |
| 8 | rs10092693 | 110,430,604 | A | G | 0.01 | 0.05 | intronic | *PKHD1L1* | . | . | . |  |
| 8 | rs7016609 | 110,431,217 | T | G | 0.01 | 0.05 | intronic | *PKHD1L1* | . | . | . |  |
| 8 | rs10458302 | 110,436,763 | A | G | 0.02 | 0.04 | intronic | *PKHD1L1* | . | . | . |  |
| 8 | rs10093885 | 110,448,635 | G | A | 0.02 | 0.02 | exonic | *PKHD1L1* | . | synonymous SNV | PKHD1L1:NM_177531:exon30:c.A3574A:p.T1192T |  |
| 8 | rs17375750 | 110,499,063 | G | T | 0.04 | 0.01 | intronic | *PKHD1L1* | . | . | . |  |
| 8 | rs2278491 | 117,656,871 | T | C | 0.04 | 0.01 | UTR3 | *EIF3H* | NM_003756:c.*374A>G | . | . |  |
| 8 | rs1436767 | 117,665,191 | A | G | 0.02 | 0.01 | ncRNA_intronic | *LOC105375713* | . | . | . |  |
| 8 | rs7843792 | 117,676,268 | A | G | 0.03 | 6.72E-03 | intronic | *EIF3H* | . | . | . |  |
| 8 | rs4876671 | 117,694,882 | A | G | 0.03 | 3.27E-03 | intronic | *EIF3H* | . | . | . |  |
| 8 | rs12675038 | 117,706,218 | C | T | 0.02 | 7.39E-03 | intronic | *EIF3H* | . | . | . |  |
| 8 | rs4876679 | 117,763,873 | A | G | 0.01 | 0.03 | intronic | *EIF3H* | . | . | . |  |
| 8 | rs2663618 | 130,869,764 | G | A | 0.02 | 9.03E-04 | intronic | *FAM49B* | . | . | . |  |
| 8 | rs881378 | 140,963,979 | T | C | 3.91E-03 | 0.02 | intronic | *TRAPPC9* | . | . | . |  |
| 8 | rs2665919 | 140,964,116 | A | C | 4.78E-03 | 0.02 | intronic | *TRAPPC9* | . | . | . |  |
| 8 | rs2035133 | 140,965,303 | G | A | 3.17E-03 | 0.02 | intronic | *TRAPPC9* | . | . | . |  |
| 8 | rs2665948 | 140,970,109 | A | C | 0.05 | 0.02 | intronic | *TRAPPC9* | . | . | . |  |
| 8 | rs11784862 | 142,203,452 | T | C | 4.92E-03 | 0.02 | UTR3 | *DENND3* | NM_001362798:c.*435T>C | . | . |  |
| 8 | rs6583607 | 143,324,399 | T | C | 0.05 | 1.06E-05 | intronic | *TSNARE1* | . | . | . |  |
| 9 | rs4741906 | 4,184,297 | C | T | 0.04 | 0.03 | intronic | *GLIS3* | . | . | . |  |
| 9 | rs10814871 | 4,193,350 | G | T | 0.03 | 0.03 | intronic | *GLIS3* | . | . | . |  |
| 9 | rs12003838 | 5,521,655 | T | G | 0.02 | 0.04 | intronic | *PDCD1LG2* | . | . | . |  |
| 9 | rs958957 | 14,434,284 | G | A | 0.03 | 0.03 | intronic | *NFIB* | . | . | . |  |
| 9 | rs6475122 | 17,244,601 | T | C | 0.03 | 1.52E-03 | intronic | *CNTLN* | . | . | . |  |
| 9 | rs601960 | 17,296,630 | G | A | 0.04 | 1.34E-03 | intronic | *CNTLN* | . | . | . |  |
| 9 | rs613966 | 17,296,926 | C | T | 0.04 | 1.80E-03 | intronic | *CNTLN* | . | . | . |  |
| 9 | rs4961551 | 17,367,673 | G | A | 0.04 | 2.35E-03 | intronic | *CNTLN* | . | . | . |  |
| 9 | rs7024224 | 23,779,696 | C | T | 0.01 | 2.16E-03 | intronic | *ELAVL2* | . | . | . |  |
| 9 | rs10968907 | 29,042,229 | T | C | 0.03 | 0.04 | intronic | *LINGO2* | . | . | . |  |
| 9 | rs10968915 | 29,061,610 | A | G | 3.15E-03 | 0.03 | intronic | *LINGO2* | . | . | . |  |
| 9 | rs12378091 | 29,088,078 | T | G | 0.03 | 0.02 | intronic | *LINGO2* | . | . | . |  |
| 9 | rs17060568 | 77,443,424 | G | A | 0.04 | 0.03 | intronic | *TRPM6* | . | . | . |  |
| 9 | rs979816 | 93,106,801 | C | T | 0.02 | 0.02 | ncRNA_intronic | *LINC01508* | . | . | . |  |
| 9 | rs1766923 | 96,802,897 | G | A | 9.99E-03 | 0.04 | intronic | *PTPDC1* | . | . | . |  |
| 9 | rs13285648 | 108,082,922 | C | T | 0.04 | 0.03 | intronic | *SLC44A1* | . | . | . |  |
| 9 | rs16926018 | 109,731,844 | C | T | 8.82E-03 | 0.04 | intronic | *ZNF462* | . | . | . |  |
| 9 | rs4838077 | 126,477,127 | C | T | 0.04 | 5.99E-03 | intronic | *DENND1A* | . | . | . |  |
| 9 | rs7034451 | 126,478,568 | C | T | 0.02 | 6.33E-03 | intronic | *DENND1A* | . | . | . |  |
| 9 | rs7022212 | 126,479,471 | T | C | 0.01 | 1.68E-03 | intronic | *DENND1A* | . | . | . |  |
| 9 | rs7034274 | 126,487,605 | T | C | 0.02 | 1.75E-03 | intronic | *DENND1A* | . | . | . |  |
| 9 | rs10454366 | 133,786,941 | A | C | 0.03 | 0.01 | intronic | *FIBCD1* | . | . | . |  |
| 9 | rs10901207 | 135,618,325 | A | G | 0.05 | 0.05 | intronic | *AK8* | . | . | . |  |
| 9 | rs3750518 | 139,982,868 | T | C | 4.35E-03 | 3.06E-03 | intronic | *MAN1B1* | . | . | . |  |
| 10 | rs11255073 | 7,338,702 | A | G | 0.04 | 0.01 | intronic | *SFMBT2* | . | . | . |  |
| 10 | rs10905149 | 7,339,814 | A | G | 0.05 | 9.41E-03 | intronic | *SFMBT2* | . | . | . |  |
| 10 | rs3758438 | 27,400,661 | T | C | 0.03 | 0.02 | UTR3 | *YME1L1* | NM_014263:c.*245A>G;NM_001253866:c.*245A>G;NM_139312:c.*245A>G | . | . |  |
| 10 | rs12782501 | 27,403,635 | C | T | 0.03 | 0.02 | intronic | *YME1L1* | . | . | . |  |
| 10 | rs4749528 | 30,376,771 | C | T | 0.03 | 2.68E-03 | intronic | *JCAD* | . | . | . |  |
| 10 | rs3810950 | 50,824,619 | A | G | 0.04 | 7.13E-03 | exonic | *CHAT* | . | synonymous SNV | CHAT:NM_001142929:exon2:c.G4G:p.A2A,CHAT:NM_020549:exon2:c.G358G:p.A120A,CHAT:NM_020984:exon2:c.G4G:p.A2A,CHAT:NM_020986:exon2:c.G4G:p.A2A,CHAT:NM_001142933:exon3:c.G112G:p.A38A,CHAT:NM_001142934:exon3:c.G4G:p.A2A,CHAT:NM_020985:exon3:c.G4G:p.A2A |  |
| 10 | rs1430882 | 71,252,206 | A | C | 0.01 | 3.86E-03 | intronic | *TSPAN15* | . | . | . |  |
| 10 | rs3911887 | 75,548,026 | A | G | 0.04 | 9.76E-06 | intronic | *ZSWIM8* | . | . | . |  |
| 10 | rs7080350 | 75,577,843 | A | G | 0.05 | 5.83E-03 | intronic | *CAMK2G* | . | . | . |  |
| 10 | rs2675662 | 75,599,127 | A | G | 0.04 | 0.02 | intronic | *CAMK2G* | . | . | . |  |
| 10 | rs2675671 | 75,632,760 | T | C | 0.04 | 0.02 | exonic | *CAMK2G* | . | synonymous SNV | CAMK2G:NM_001204492:exon2:c.G147G:p.K49K,CAMK2G:NM_001222:exon2:c.G147G:p.K49K,CAMK2G:NM_001320898:exon2:c.G147G:p.K49K,CAMK2G:NM_001367514:exon2:c.G123G:p.K41K,CAMK2G:NM_001367516:exon2:c.G147G:p.K49K,CAMK2G:NM_001367517:exon2:c.G147G:p.K49K,CAMK2G:NM_001367518:exon2:c.G147G:p.K49K,CAMK2G:NM_001367519:exon2:c.G147G:p.K49K,CAMK2G:NM_001367520:exon2:c.G147G:p.K49K,CAMK2G:NM_001367521:exon2:c.G147G:p.K49K,CAMK2G:NM_001367522:exon2:c.G147G:p.K49K,CAMK2G:NM_001367523:exon2:c.G147G:p.K49K,CAMK2G:NM_001367525:exon2:c.G123G:p.K41K,CAMK2G:NM_001367526:exon2:c.G147G:p.K49K,CAMK2G:NM_001367527:exon2:c.G147G:p.K49K,CAMK2G:NM_001367528:exon2:c.G147G:p.K49K,CAMK2G:NM_001367529:exon2:c.G147G:p.K49K,CAMK2G:NM_001367530:exon2:c.G147G:p.K49K,CAMK2G:NM_001367531:exon2:c.G147G:p.K49K,CAMK2G:NM_001367532:exon2:c.G123G:p.K41K,CAMK2G:NM_001367533:exon2:c.G147G:p.K49K,CAMK2G:NM_001367534:exon2:c.G147G:p.K49K,CAMK2G:NM_001367535:exon2:c.G147G:p.K49K,CAMK2G:NM_001367536:exon2:c.G147G:p.K49K,CAMK2G:NM_001367541:exon2:c.G147G:p.K49K,CAMK2G:NM_001367543:exon2:c.G147G:p.K49K,CAMK2G:NM_001367544:exon2:c.G147G:p.K49K,CAMK2G:NM_001367545:exon2:c.G147G:p.K49K,CAMK2G:NM_001367546:exon2:c.G147G:p.K49K,CAMK2G:NM_001367547:exon2:c.G147G:p.K49K,CAMK2G:NM_001367548:exon2:c.G147G:p.K49K,CAMK2G:NM_172169:exon2:c.G147G:p.K49K,CAMK2G:NM_172170:exon2:c.G147G:p.K49K,CAMK2G:NM_172171:exon2:c.G147G:p.K49K,CAMK2G:NM_172173:exon2:c.G147G:p.K49K |  |
| 10 | rs2986401 | 97,382,659 | T | C | 3.07E-03 | 5.35E-03 | intronic | *ALDH18A1* | . | . | . |  |
| 10 | rs501819 | 97,383,926 | C | A | 2.60E-03 | 6.18E-03 | intronic | *ALDH18A1* | . | . | . |  |
| 10 | rs10509688 | 97,385,242 | G | A | 0.02 | 0.01 | intronic | *ALDH18A1* | . | . | . |  |
| 10 | rs12773566 | 97,390,566 | C | T | 0.04 | 0.03 | intronic | *ALDH18A1* | . | . | . |  |
| 10 | rs7894648 | 97,431,369 | C | T | 4.53E-03 | 2.27E-03 | intronic | *TCTN3* | . | . | . |  |
| 10 | rs10882651 | 97,438,823 | T | C | 5.17E-03 | 2.57E-03 | intronic | *TCTN3* | . | . | . |  |
| 10 | rs10882652 | 97,441,326 | A | G | 4.12E-03 | 2.64E-03 | intronic | *TCTN3* | . | . | . |  |
| 10 | rs4481960 | 97,524,313 | C | A | 0.02 | 0.01 | ncRNA_intronic | *ENTPD1-AS1* | . | . | . |  |
| 10 | rs10786240 | 97,666,538 | C | T | 1.27E-03 | 0.02 | ncRNA_intronic | *ENTPD1-AS1* | . | . | . |  |
| 10 | rs10882686 | 97,668,427 | A | G | 2.99E-03 | 0.02 | ncRNA_intronic | *ENTPD1-AS1* | . | . | . |  |
| 10 | rs1048445 | 99,116,903 | T | C | 0.02 | 8.13E-03 | exonic | *RRP12* | . | synonymous SNV | RRP12:NM_001284337:exon31:c.G3542G:p.R1181R,RRP12:NM_001145114:exon32:c.G3659G:p.R1220R,RRP12:NM_015179:exon34:c.G3842G:p.R1281R |  |
| 10 | rs4918274 | 108,729,960 | A | G | 0.03 | 6.56E-03 | intronic | *SORCS1* | . | . | . |  |
| 10 | rs17681175 | 121,603,574 | A | G | 0.04 | 0.04 | intronic | *MCMBP* | . | . | . |  |
| 11 | rs2618508 | 14,100,036 | A | G | 0.02 | 0.03 | intronic | *SPON1* | . | . | . |  |
| 11 | rs6486166 | 14,105,497 | T | C | 0.02 | 0.03 | intronic | *SPON1* | . | . | . |  |
| 11 | rs3802967 | 18,344,064 | C | T | 0.04 | 9.41E-03 | UTR5 | *GTF2H1* | NM_001142307:c.-10558C>T | . | . |  |
| 11 | rs2028603 | 19,906,795 | T | C | 0.04 | 0.01 | intronic | *NAV2* | . | . | . |  |
| 11 | rs11030066 | 27,618,490 | T | C | 0.01 | 1.11E-03 | ncRNA_intronic | *BDNF-AS* | . | . | . |  |
| 11 | rs4923461 | 27,656,910 | G | A | 0.01 | 6.92E-04 | ncRNA_intronic | *BDNF-AS* | . | . | . |  |
| 11 | rs17571754 | 28,290,578 | C | T | 0.05 | 5.26E-03 | intronic | *METTL15* | . | . | . |  |
| 11 | rs2076622 | 33,631,423 | G | A | 0.03 | 0.02 | exonic | *KIAA1549L* | . | synonymous SNV | KIAA1549L:NM_012194:exon15:c.G5190A:p.K1730K |  |
| 11 | rs7941404 | 47,712,213 | T | C | 0.05 | 4.83E-04 | exonic | *AGBL2* | . | synonymous SNV | AGBL2:NM_024783:exon10:c.G1046G:p.R349R |  |
| 11 | rs11039369 | 47,775,952 | G | A | 0.02 | 8.19E-04 | intronic | *FNBP4* | . | . | . |  |
| 11 | rs2282492 | 63,918,589 | C | T | 6.18E-03 | 0.03 | intronic | *MACROD1* | . | . | . |  |
| 11 | rs11603042 | 64,003,843 | T | G | 5.16E-03 | 0.05 | intronic | *VEGFB* | . | . | . |  |
| 11 | rs594942 | 64,006,292 | T | C | 3.13E-03 | 0.03 | UTR3 | *VEGFB* | NM_003377:c.*487T>C;NM_001243733:c.*443T>C | . | . |  |
| 11 | rs7932437 | 64,373,504 | C | T | 0.05 | 0.02 | downstream | *NRXN2* | dist=142 | . | . |  |
| 11 | rs1785633 | 66,107,527 | A | C | 0.04 | 0.02 | intronic | *BRMS1* | . | . | . |  |
| 11 | rs947978 | 66,108,660 | T | C | 0.05 | 0.02 | intronic | *BRMS1* | . | . | . |  |
| 11 | rs1892940 | 66,111,251 | T | C | 0.05 | 0.02 | intronic | *BRMS1* | . | . | . |  |
| 11 | rs2066490 | 67,160,933 | A | G | 0.05 | 0.04 | UTR5 | *RAD9A* | NM_001243224:c.-28A>G | . | . |  |
| 11 | rs7944627 | 70,257,019 | C | A | 0.05 | 0.04 | intronic | *CTTN* | . | . | . |  |
| 11 | rs11236240 | 70,267,026 | G | A | 0.02 | 0.05 | intronic | *CTTN* | . | . | . |  |
| 11 | rs12801756 | 70,272,042 | G | A | 0.04 | 0.02 | intronic | *CTTN* | . | . | . |  |
| 11 | rs7106080 | 78,150,592 | A | G | 0.01 | 0.03 | intronic | *NARS2* | . | . | . |  |
| 11 | rs10899538 | 78,237,749 | T | C | 0.02 | 0.05 | intronic | *NARS2* | . | . | . |  |
| 11 | rs4565922 | 78,260,699 | G | A | 0.03 | 0.02 | ncRNA_intronic | *LOC101928896* | . | . | . |  |
| 11 | rs9645693 | 84,343,679 | G | A | 0.04 | 0.04 | intronic | *DLG2* | . | . | . |  |
| 11 | rs1445504 | 85,584,879 | A | G | 0.05 | 0.03 | intronic | *CCDC83* | . | . | . |  |
| 11 | rs11234457 | 85,591,041 | T | C | 4.61E-03 | 0.04 | intronic | *CCDC83* | . | . | . |  |
| 11 | rs17817308 | 85,593,529 | A | G | 0.04 | 0.03 | intronic | *CCDC83* | . | . | . |  |
| 11 | rs17817331 | 85,595,338 | T | C | 0.04 | 0.03 | intronic | *CCDC83* | . | . | . |  |
| 11 | rs17744699 | 85,597,163 | T | C | 0.04 | 0.03 | intronic | *CCDC83* | . | . | . |  |
| 11 | rs10831496 | 88,557,991 | A | G | 0.05 | 0.04 | intronic | *GRM5* | . | . | . |  |
| 11 | rs480015 | 88,564,533 | A | C | 0.05 | 0.03 | intronic | *GRM5* | . | . | . |  |
| 11 | rs316086 | 88,584,160 | A | C | 0.05 | 5.50E-04 | intronic | *GRM5* | . | . | . |  |
| 11 | rs4370966 | 113,231,691 | A | G | 6.31E-03 | 3.33E-03 | intronic | *TTC12* | . | . | . |  |
| 11 | rs17600713 | 113,259,947 | A | G | 0.01 | 1.62E-03 | intronic | *ANKK1* | . | . | . |  |
| 11 | rs2734849 | 113,270,160 | G | A | 0.01 | 7.04E-06 | exonic | *ANKK1* | . | synonymous SNV | ANKK1:NM_178510:exon8:c.A1469A:p.H490H |  |
| 11 | rs12363125 | 113,285,916 | T | C | 0.01 | 1.90E-07 | intronic | *DRD2* | . | . | . |  |
| 11 | rs2734838 | 113,286,501 | G | A | 8.81E-03 | 1.93E-07 | intronic | *DRD2* | . | . | . |  |
| 11 | rs2734831 | 113,293,596 | G | T | 2.34E-03 | 3.80E-07 | intronic | *DRD2* | . | . | . |  |
| 11 | rs1076563 | 113,295,909 | C | A | 1.41E-03 | 4.28E-07 | intronic | *DRD2* | . | . | . |  |
| 11 | rs1116313 | 113,296,107 | G | A | 1.37E-03 | 4.70E-07 | intronic | *DRD2* | . | . | . |  |
| 11 | rs1079597 | 113,296,286 | T | C | 0.04 | 5.30E-07 | intronic | *DRD2* | . | . | . |  |
| 11 | rs7131440 | 113,299,910 | T | C | 4.16E-03 | 4.27E-07 | intronic | *DRD2* | . | . | . |  |
| 11 | rs12364051 | 113,305,314 | G | A | 4.71E-03 | 1.00E-06 | intronic | *DRD2* | . | . | . |  |
| 11 | rs4630328 | 113,334,209 | A | G | 0.03 | 2.80E-07 | intronic | *DRD2* | . | . | . |  |
| 11 | rs11601054 | 113,336,172 | A | G | 0.02 | 2.74E-07 | intronic | *DRD2* | . | . | . |  |
| 11 | rs4936280 | 113,570,405 | T | C | 0.05 | 2.05E-03 | exonic | *TMPRSS5* | . | synonymous SNV | TMPRSS5:NM_001288751:exon3:c.A90G:p.A30A,TMPRSS5:NM_001288752:exon3:c.A117G:p.A39A,TMPRSS5:NM_030770:exon3:c.A117G:p.A39A |  |
| 11 | rs3802856 | 113,570,490 | T | C | 0.05 | 2.02E-03 | intronic | *TMPRSS5* | . | . | . |  |
| 11 | rs7926267 | 113,576,932 | A | G | 0.04 | 2.34E-03 | intronic | *TMPRSS5* | . | . | . |  |
| 11 | rs17613359 | 113,617,882 | T | G | 0.02 | 1.38E-03 | intronic | *ZW10* | . | . | . |  |
| 11 | rs2519190 | 114,007,908 | C | T | 6.43E-03 | 5.12E-04 | intronic | *ZBTB16* | . | . | . |  |
| 11 | rs11215485 | 115,189,893 | T | C | 2.46E-03 | 0.05 | intronic | *CADM1* | . | . | . |  |
| 11 | rs4935791 | 122,210,042 | A | G | 0.04 | 0.04 | ncRNA_intronic | *MIR100HG* | . | . | . |  |
| 11 | rs10502283 | 123,753,265 | T | C | 0.02 | 9.63E-03 | downstream | *TMEM225* | dist=362 | . | . |  |
| 11 | rs654723 | 128,586,155 | A | C | 7.19E-04 | 0.05 | intronic | *FLI1* | . | . | . |  |
| 11 | rs1448363 | 131,320,190 | T | C | 0.02 | 0.04 | intronic | *NTM* | . | . | . |  |
| 11 | rs7937053 | 132,015,077 | C | T | 0.01 | 0.01 | intronic | *NTM* | . | . | . |  |
| 11 | rs7481500 | 133,334,350 | T | G | 0.01 | 9.84E-04 | intronic | *OPCML* | . | . | . |  |
| 12 | rs2907608 | 2,997,397 | G | A | 0.04 | 0.01 | exonic | *RHNO1* | . | synonymous SNV | RHNO1:NM_001252499:exon3:c.G489A:p.S163S,RHNO1:NM_001252500:exon3:c.G447A:p.S149S,RHNO1:NM_001257097:exon3:c.G489A:p.S163S,RHNO1:NM_001257098:exon3:c.G489A:p.S163S |  |
| 12 | rs2070990 | 2,998,892 | A | G | 0.03 | 3.85E-03 | downstream | *RHNO1* | dist=201 | . | . |  |
| 12 | rs11830917 | 12,677,613 | G | A | 5.85E-03 | 0.02 | intronic | *DUSP16* | . | . | . |  |
| 12 | rs4763837 | 12,679,124 | G | A | 7.23E-03 | 0.01 | intronic | *DUSP16* | . | . | . |  |
| 12 | rs16908222 | 12,680,166 | A | G | 3.23E-03 | 0.01 | intronic | *DUSP16* | . | . | . |  |
| 12 | rs9645754 | 14,124,144 | G | A | 0.03 | 0.03 | intronic | *GRIN2B* | . | . | . |  |
| 12 | rs7134291 | 14,128,322 | A | G | 0.02 | 0.03 | intronic | *GRIN2B* | . | . | . |  |
| 12 | rs11174292 | 40,317,017 | G | T | 0.03 | 0.04 | intronic | *SLC2A13* | . | . | . |  |
| 12 | rs11174370 | 40,330,822 | T | C | 0.03 | 0.05 | intronic | *SLC2A13* | . | . | . |  |
| 12 | rs12817211 | 50,579,398 | T | C | 5.01E-03 | 0.03 | intronic | *LIMA1* | . | . | . |  |
| 12 | rs11170287 | 53,241,626 | G | A | 0.02 | 0.02 | intronic | *KRT78* | . | . | . |  |
| 12 | rs12811821 | 62,751,242 | G | A | 0.04 | 4.50E-05 | intronic | *USP15* | . | . | . |  |
| 12 | rs12424732 | 62,778,567 | T | C | 0.01 | 0.04 | intronic | *USP15* | . | . | . |  |
| 12 | rs11174461 | 62,797,683 | A | G | 0.02 | 3.15E-04 | intronic | *USP15* | . | . | . |  |
| 12 | rs1880979 | 90,078,050 | T | G | 0.02 | 3.21E-03 | intronic | *ATP2B1* | . | . | . |  |
| 12 | rs1420391 | 98,123,911 | T | G | 0.01 | 0.03 | ncRNA_exonic | *LOC643711* | . | . | . |  |
| 12 | rs703616 | 104,020,232 | C | T | 0.02 | 0.02 | intronic | *STAB2* | . | . | . |  |
| 12 | rs703618 | 104,021,263 | A | G | 0.01 | 0.03 | intronic | *STAB2* | . | . | . |  |
| 12 | rs703619 | 104,022,088 | A | G | 0.01 | 0.03 | intronic | *STAB2* | . | . | . |  |
| 12 | rs11068590 | 118,072,155 | G | A | 4.96E-03 | 0.02 | intronic | *KSR2* | . | . | . |  |
| 12 | rs10850864 | 118,073,086 | T | C | 5.13E-03 | 0.02 | intronic | *KSR2* | . | . | . |  |
| 12 | rs10850868 | 118,074,320 | G | T | 6.67E-03 | 0.02 | intronic | *KSR2* | . | . | . |  |
| 12 | rs10850872 | 118,077,971 | C | T | 0.02 | 0.02 | intronic | *KSR2* | . | . | . |  |
| 12 | rs2297478 | 125,591,844 | T | C | 0.01 | 0.03 | intronic | *AACS* | . | . | . |  |
| 12 | rs7132026 | 125,678,096 | T | C | 0.04 | 0.03 | intronic | *TMEM132B* | . | . | . |  |
| 12 | rs326387 | 125,805,148 | T | C | 0.04 | 1.17E-03 | intronic | *TMEM132B* | . | . | . |  |
| 12 | rs551601 | 125,810,958 | T | C | 0.03 | 1.47E-03 | intronic | *TMEM132B* | . | . | . |  |
| 12 | rs550677 | 125,811,056 | A | G | 0.02 | 1.47E-03 | intronic | *TMEM132B* | . | . | . |  |
| 12 | rs375222 | 125,811,398 | T | C | 0.02 | 1.34E-03 | intronic | *TMEM132B* | . | . | . |  |
| 13 | rs9506430 | 20,717,743 | T | C | 0.04 | 0.04 | intronic | *GJA3* | . | . | . |  |
| 13 | rs4769086 | 20,720,447 | T | C | 0.04 | 0.02 | intronic | *GJA3* | . | . | . |  |
| 13 | rs4769088 | 20,722,066 | A | G | 0.04 | 0.02 | intronic | *GJA3* | . | . | . |  |
| 13 | rs17350833 | 46,537,757 | A | G | 0.02 | 0.04 | UTR3 | *ZC3H13* | NM_015070:c.*200T>C | . | . |  |
| 13 | rs713342 | 63,769,416 | A | G | 0.03 | 0.01 | ncRNA_intronic | *LINC00376* | . | . | . |  |
| 13 | rs12874868 | 73,390,447 | G | A | 0.03 | 0.01 | intronic | *PIBF1* | . | . | . |  |
| 13 | rs17794787 | 80,489,814 | G | A | 0.04 | 0.04 | ncRNA_intronic | *LINC00382* | . | . | . |  |
| 13 | rs9561932 | 96,347,177 | T | C | 1.60E-03 | 3.08E-03 | intronic | *DNAJC3* | . | . | . |  |
| 13 | rs9561935 | 96,354,443 | A | G | 0.02 | 4.30E-03 | intronic | *DNAJC3* | . | . | . |  |
| 13 | rs7995306 | 96,411,220 | C | T | 0.02 | 6.26E-03 | intronic | *DNAJC3* | . | . | . |  |
| 13 | rs11070154 | 96,506,664 | G | A | 2.94E-03 | 5.46E-03 | exonic | *UGGT2* | . | synonymous SNV | UGGT2:NM_020121:exon35:c.T4074T:p.T1358T |  |
| 13 | rs12876018 | 96,540,204 | G | T | 0.01 | 2.98E-03 | exonic | *UGGT2* | . | synonymous SNV | UGGT2:NM_020121:exon26:c.A2980A:p.M994M |  |
| 13 | rs1537030 | 96,545,289 | G | A | 0.01 | 3.75E-03 | intronic | *UGGT2* | . | . | . |  |
| 13 | rs35836619 | 96,558,852 | A | G | 4.26E-03 | 2.84E-03 | intronic | *UGGT2* | . | . | . |  |
| 13 | rs2262077 | 96,801,507 | A | G | 0.05 | 0.02 | intronic | *HS6ST3* | . | . | . |  |
| 13 | rs943560 | 96,851,824 | G | A | 0.04 | 1.41E-04 | intronic | *HS6ST3* | . | . | . |  |
| 13 | rs9302094 | 96,852,740 | C | A | 0.01 | 2.73E-05 | intronic | *HS6ST3* | . | . | . |  |
| 13 | rs9525149 | 96,855,782 | A | G | 0.02 | 1.19E-04 | intronic | *HS6ST3* | . | . | . |  |
| 13 | rs9590371 | 96,860,076 | A | G | 9.90E-03 | 2.41E-05 | intronic | *HS6ST3* | . | . | . |  |
| 13 | rs1327634 | 96,861,302 | G | A | 0.03 | 7.91E-05 | intronic | *HS6ST3* | . | . | . |  |
| 13 | rs12428610 | 96,865,350 | G | A | 0.03 | 1.13E-04 | intronic | *HS6ST3* | . | . | . |  |
| 13 | rs9516648 | 96,895,842 | T | C | 0.04 | 2.00E-05 | intronic | *HS6ST3* | . | . | . |  |
| 13 | rs1927801 | 96,905,617 | A | G | 0.02 | 8.77E-05 | intronic | *HS6ST3* | . | . | . |  |
| 13 | rs1927796 | 96,918,833 | G | A | 0.01 | 1.39E-04 | intronic | *HS6ST3* | . | . | . |  |
| 13 | rs12871890 | 96,987,910 | A | G | 0.04 | 6.62E-06 | intronic | *HS6ST3* | . | . | . |  |
| 13 | rs7323117 | 100,300,559 | T | C | 0.01 | 0.03 | intronic | *CLYBL* | . | . | . |  |
| 13 | rs9515307 | 111,543,043 | A | G | 4.54E-03 | 0.02 | intronic | *ANKRD10* | . | . | . |  |
| 13 | rs7994149 | 111,543,106 | T | C | 7.86E-03 | 0.02 | intronic | *ANKRD10* | . | . | . |  |
| 14 | rs872072 | 20,859,013 | G | A | 0.02 | 0.03 | intronic | *TEP1* | . | . | . |  |
| 14 | rs8023081 | 21,884,887 | G | A | 0.03 | 0.05 | intronic | *CHD8* | . | . | . |  |
| 14 | rs3958 | 35,070,371 | A | G | 0.05 | 0.01 | intronic | *SNX6* | . | . | . |  |
| 14 | rs7161203 | 35,087,218 | C | A | 0.04 | 0.05 | intronic | *SNX6* | . | . | . |  |
| 14 | rs2275025 | 57,046,829 | A | C | 0.03 | 0.01 | intronic | *TMEM260* | . | . | . |  |
| 14 | rs3737171 | 57,052,511 | T | G | 0.03 | 0.01 | exonic | *TMEM260* | . | synonymous SNV | TMEM260:NM_017799:exon3:c.G225G:p.T75T |  |
| 14 | rs2151774 | 57,056,374 | T | C | 0.02 | 0.01 | intronic | *TMEM260* | . | . | . |  |
| 14 | rs8019974 | 67,986,275 | C | T | 0.03 | 0.02 | intronic | *TMEM229B* | . | . | . |  |
| 14 | rs714975 | 72,416,763 | T | C | 0.02 | 0.05 | intronic | *RGS6* | . | . | . |  |
| 14 | rs1286146 | 91,474,448 | G | A | 0.03 | 0.02 | intronic | *RPS6KA5* | . | . | . |  |
| 14 | rs7147377 | 93,857,868 | G | T | 0.01 | 0.04 | intronic | *UNC79* | . | . | . |  |
| 14 | rs3825559 | 102,198,596 | T | C | 0.04 | 5.46E-03 | ncRNA_exonic | *LINC00239* | . | . | . |  |
| 14 | rs8009458 | 102,199,154 | A | G | 0.05 | 7.81E-03 | downstream | *LINC00239* | dist=292 | . | . |  |
| 15 | rs28649825 | 27,584,009 | C | T | 0.02 | 0.02 | intronic | *GABRG3* | . | . | . |  |
| 15 | rs1821715 | 40,174,920 | T | C | 2.04E-03 | 0.03 | intronic | *GPR176* | . | . | . |  |
| 15 | rs1047552 | 63,597,857 | G | T | 0.04 | 0.01 | exonic | *APH1B* | . | synonymous SNV | APH1B:NM_001145646:exon5:c.T528T:p.F176F,APH1B:NM_031301:exon6:c.T651T:p.F217F |  |
| 15 | rs4887111 | 74,028,285 | G | A | 0.04 | 0.02 | UTR3 | *INSYN1* | NM_001303254:c.*3973C>T;NM_001039614:c.*3973C>T | . | . |  |
| 15 | rs12442096 | 74,038,773 | T | C | 0.01 | 0.01 | intronic | *INSYN1* | . | . | . |  |
| 15 | rs2280214 | 89,442,606 | T | C | 0.04 | 6.73E-04 | UTR3 | *MFGE8* | NM_001114614:c.*20A>G;NM_001310321:c.*20A>G;NM_001310320:c.*20A>G;NM_001310319:c.*20A>G;NM_005928:c.*20A>G | . | . |  |
| 15 | rs3825901 | 101,949,168 | T | C | 0.02 | 0.05 | intronic | *PCSK6* | . | . | . |  |
| 16 | rs3842949 | 360,303 | T | C | 0.04 | 0.02 | intronic | *AXIN1* | . | . | . |  |
| 16 | rs11861673 | 7,362,803 | C | T | 0.02 | 6.12E-03 | intronic | *RBFOX1* | . | . | . |  |
| 16 | rs350281 | 12,231,739 | G | T | 0.01 | 0.02 | intronic | *SNX29* | . | . | . |  |
| 16 | rs209845 | 12,416,319 | G | A | 0.02 | 3.48E-04 | intronic | *SNX29* | . | . | . |  |
| 16 | rs889811 | 12,505,469 | G | T | 7.50E-03 | 5.62E-05 | intronic | *SNX29* | . | . | . |  |
| 16 | rs889809 | 12,506,070 | G | A | 0.02 | 7.08E-05 | intronic | *SNX29* | . | . | . |  |
| 16 | rs7499306 | 20,555,474 | C | T | 0.05 | 0.05 | intronic | *ACSM2B* | . | . | . |  |
| 16 | rs13336754 | 24,801,979 | T | C | 0.02 | 4.72E-04 | exonic | *TNRC6A* | . | synonymous SNV | TNRC6A:NM_001330520:exon6:c.C2016C:p.S672S,TNRC6A:NM_001351850:exon6:c.C2043C:p.S681S,TNRC6A:NM_014494:exon6:c.C2016C:p.S672S |  |
| 16 | rs2356837 | 50,120,893 | C | T | 0.02 | 0.05 | intronic | *HEATR3* | . | . | . |  |
| 16 | rs1064448 | 50,350,883 | G | T | 0.03 | 0.03 | UTR3 | *ADCY7;BRD7* | NM_001114:c.*1467G>T;NM_013263:c.*2239C>A;NM_001173984:c.*2239C>A | . | . |  |
| 16 | rs4785408 | 50,361,739 | C | T | 0.01 | 0.04 | intronic | *BRD7* | . | . | . |  |
| 16 | rs4785409 | 50,362,386 | C | T | 0.01 | 0.03 | intronic | *BRD7* | . | . | . |  |
| 16 | rs7205089 | 50,364,398 | C | A | 0.01 | 0.03 | intronic | *BRD7* | . | . | . |  |
| 16 | rs11645288 | 51,172,677 | A | G | 1.34E-03 | 0.02 | exonic | *SALL1* | . | synonymous SNV | SALL1:NM_001127892:exon2:c.C3165C:p.H1055H,SALL1:NM_002968:exon2:c.C3456C:p.H1152H |  |
| 16 | rs1421085 | 53,800,954 | C | T | 0.02 | 2.26E-10 | intronic | *FTO* | . | . | . |  |
| 16 | rs3751812 | 53,818,460 | T | G | 0.03 | 2.95E-10 | intronic | *FTO* | . | . | . |  |
| 16 | rs9941349 | 53,825,488 | T | C | 0.04 | 6.22E-11 | intronic | *FTO* | . | . | . |  |
| 16 | rs1345320 | 55,461,791 | A | G | 0.05 | 0.03 | ncRNA_intronic | *MMP2-AS1* | . | . | . |  |
| 16 | rs2640799 | 60,028,973 | A | G | 0.01 | 0.02 | ncRNA_intronic | *LINC02141* | . | . | . |  |
| 16 | rs2549692 | 60,031,823 | A | G | 0.01 | 0.02 | ncRNA_intronic | *LINC02141* | . | . | . |  |
| 16 | rs12599391 | 69,605,349 | C | T | 8.35E-03 | 5.07E-03 | intronic | *NFAT5* | . | . | . |  |
| 16 | rs244418 | 69,622,762 | A | G | 6.32E-03 | 5.81E-03 | intronic | *NFAT5* | . | . | . |  |
| 16 | rs2291959 | 69,873,992 | T | G | 1.11E-03 | 3.85E-03 | intronic | *WWP2* | . | . | . |  |
| 16 | rs9940315 | 69,876,164 | A | G | 1.15E-03 | 3.88E-03 | intronic | *WWP2* | . | . | . |  |
| 16 | rs1500337 | 69,901,884 | T | C | 0.05 | 8.61E-03 | intronic | *WWP2* | . | . | . |  |
| 16 | rs4985377 | 69,948,497 | A | C | 3.35E-03 | 0.03 | intronic | *WWP2* | . | . | . |  |
| 16 | rs2270842 | 69,970,000 | T | G | 0.02 | 0.05 | intronic | *WWP2* | . | . | . |  |
| 16 | rs3748388 | 69,974,448 | C | A | 6.61E-03 | 0.05 | UTR3 | *WWP2* | NM_007014:c.*605C>A;NM_001270454:c.*605C>A;NM_001270453:c.*605C>A;NM_199424:c.*605C>A | . | . |  |
| 16 | rs1052429 | 69,975,360 | G | A | 8.50E-03 | 0.05 | UTR3 | *WWP2* | NM_007014:c.*1517G>A;NM_001270454:c.*1517G>A;NM_001270453:c.*1517G>A;NM_199424:c.*1517G>A | . | . |  |
| 16 | rs9922176 | 70,092,742 | C | T | 2.44E-03 | 0.04 | ncRNA_intronic | *PDXDC2P-NPIPB14P* | . | . | . |  |
| 16 | rs17176204 | 88,830,836 | T | C | 0.04 | 0.03 | intronic | *PIEZO1* | . | . | . |  |
| 17 | rs7225087 | 477,471 | C | T | 0.03 | 0.04 | intronic | *VPS53* | . | . | . |  |
| 17 | rs838369 | 578,180 | C | T | 0.02 | 0.02 | intronic | *VPS53* | . | . | . |  |
| 17 | rs2295479 | 636,822 | T | C | 0.03 | 0.01 | intronic | *TLCD3A* | . | . | . |  |
| 17 | rs1045481 | 648,157 | A | G | 0.03 | 0.05 | exonic | *GEMIN4* | . | synonymous SNV | GEMIN4:NM_015721:exon2:c.C3126C:p.I1042I |  |
| 17 | rs16953091 | 3,408,973 | G | A | 0.02 | 0.05 | intronic | *SPATA22* | . | . | . |  |
| 17 | rs3744656 | 9,141,323 | G | T | 8.55E-03 | 0.03 | intronic | *NTN1* | . | . | . |  |
| 17 | rs2280777 | 18,041,507 | T | C | 0.02 | 4.11E-03 | exonic | *MYO15A* | . | synonymous SNV | MYO15A:NM_016239:exon16:c.C4954C:p.L1652L |  |
| 17 | rs860568 | 18,079,075 | T | C | 0.05 | 3.53E-03 | intronic | *MYO15A* | . | . | . |  |
| 17 | rs12936694 | 18,099,962 | G | A | 0.01 | 3.37E-03 | intronic | *ALKBH5* | . | . | . |  |
| 17 | rs11080056 | 25,404,049 | C | T | 0.04 | 0.03 | ncRNA_intronic | *LOC105371703* | . | . | . | y |
| 17 | rs11652017 | 25,410,639 | T | C | 0.04 | 0.03 | ncRNA_intronic | *LOC105371703* | . | . | . | y |
| 17 | rs11650889 | 25,410,734 | A | G | 0.04 | 0.03 | ncRNA_intronic | *LOC105371703* | . | . | . | y |
| 17 | rs12453548 | 31,809,526 | A | G | 0.03 | 0.03 | intronic | *ASIC2* | . | . | . |  |
| 17 | rs12451410 | 31,809,558 | G | A | 0.03 | 0.02 | intronic | *ASIC2* | . | . | . |  |
| 17 | rs2285740 | 36,068,728 | C | T | 4.22E-03 | 8.30E-03 | intronic | *HNF1B* | . | . | . |  |
| 17 | rs1724424 | 43,779,962 | G | T | 1.63E-03 | 0.01 | intronic | *LINC02210-CRHR1* | . | . | . |  |
| 17 | rs1880753 | 43,811,260 | A | G | 0.01 | 0.04 | intronic | *LINC02210-CRHR1* | . | . | . |  |
| 17 | rs12944235 | 43,813,221 | A | G | 4.34E-03 | 0.04 | intronic | *LINC02210-CRHR1* | . | . | . |  |
| 17 | rs8078967 | 44,008,101 | T | C | 0.01 | 2.12E-03 | intronic | *MAPT* | . | . | . |  |
| 17 | rs8070723 | 44,081,064 | G | A | 0.05 | 3.44E-05 | intronic | *MAPT* | . | . | . |  |
| 17 | rs4378657 | 46,023,694 | A | G | 0.04 | 0.03 | exonic | *PNPO* | . | synonymous SNV | PNPO:NM_018129:exon6:c.G552G:p.L184L |  |
| 17 | rs9904287 | 65,168,241 | A | G | 0.05 | 0.05 | intronic | *HELZ* | . | . | . |  |
| 17 | rs7218955 | 65,197,158 | A | G | 0.02 | 0.04 | intronic | *HELZ* | . | . | . |  |
| 18 | rs16950868 | 6,956,238 | A | C | 0.03 | 0.02 | ncRNA_exonic | *LOC101927188* | . | . | . |  |
| 18 | rs17656284 | 28,573,167 | A | G | 0.04 | 0.02 | UTR3 | *DSC3* | NM_024423:c.*1188T>C;NM_001941:c.*974T>C | . | . |  |
| 18 | rs2612321 | 39,091,372 | G | A | 0.04 | 0.02 | ncRNA_intronic | *KC6* | . | . | . |  |
| 18 | rs16956114 | 50,623,189 | A | G | 6.28E-03 | 0.01 | intronic | *DCC* | . | . | . |  |
| 18 | rs8092993 | 50,670,111 | A | G | 8.31E-03 | 5.05E-03 | intronic | *DCC* | . | . | . |  |
| 18 | rs11082992 | 50,924,132 | C | T | 0.05 | 1.07E-03 | intronic | *DCC* | . | . | . |  |
| 18 | rs9961807 | 53,819,904 | T | C | 2.82E-04 | 0.03 | ncRNA_intronic | *LOC642484* | . | . | . |  |
| 18 | rs1789600 | 53,848,399 | T | C | 2.19E-03 | 0.01 | ncRNA_intronic | *LOC642484* | . | . | . |  |
| 18 | rs1789597 | 53,852,255 | G | T | 1.96E-03 | 0.02 | ncRNA_intronic | *LOC642484* | . | . | . |  |
| 18 | rs12454365 | 54,396,215 | A | G | 0.03 | 0.01 | intronic | *WDR7* | . | . | . |  |
| 18 | rs2032217 | 63,426,979 | A | G | 0.04 | 0.02 | intronic | *CDH7* | . | . | . |  |
| 18 | rs12956148 | 63,429,049 | A | C | 0.04 | 0.02 | intronic | *CDH7* | . | . | . |  |
| 18 | rs1562724 | 77,169,571 | T | C | 3.77E-03 | 0.05 | intronic | *NFATC1* | . | . | . |  |
| 19 | rs11669897 | 630,833 | T | G | 0.01 | 0.05 | intronic | *POLRMT* | . | . | . |  |
| 19 | rs418115 | 4,977,478 | C | T | 0.03 | 0.04 | intronic | *KDM4B* | . | . | . |  |
| 19 | rs875569 | 10,668,953 | A | G | 2.52E-03 | 3.85E-03 | intronic | *KRI1* | . | . | . |  |
| 19 | rs3745249 | 10,670,992 | T | C | 3.50E-03 | 4.35E-03 | exonic | *KRI1* | . | synonymous SNV | KRI1:NM_023008:exon9:c.G796G:p.E266E |  |
| 19 | rs12984043 | 10,672,493 | T | C | 1.73E-03 | 0.02 | exonic | *KRI1* | . | synonymous SNV | KRI1:NM_023008:exon5:c.G412G:p.G138G |  |
| 19 | rs2742313 | 10,799,750 | T | C | 0.05 | 0.04 | intronic | *ILF3* | . | . | . |  |
| 19 | rs971694 | 17,422,833 | T | C | 3.73E-03 | 0.02 | intronic | *DDA1* | . | . | . |  |
| 19 | rs8111532 | 17,835,520 | A | G | 0.02 | 0.03 | intronic | *MAP1S* | . | . | . |  |
| 19 | rs2287860 | 17,844,325 | T | C | 5.32E-03 | 0.04 | intronic | *MAP1S* | . | . | . |  |
| 19 | rs7253117 | 17,844,894 | A | G | 4.49E-03 | 0.03 | intronic | *MAP1S* | . | . | . |  |
| 19 | rs11673604 | 18,540,988 | T | C | 0.04 | 0.02 | intronic | *SSBP4* | . | . | . |  |
| 19 | rs4808136 | 18,618,867 | G | A | 0.04 | 0.01 | intronic | *ELL* | . | . | . |  |
| 19 | rs271626 | 18,627,067 | T | C | 0.03 | 0.03 | intronic | *ELL* | . | . | . |  |
| 19 | rs7255674 | 31,788,925 | C | A | 9.60E-03 | 0.02 | intronic | *TSHZ3* | . | . | . |  |
| 19 | rs8100423 | 33,109,003 | G | A | 0.05 | 9.33E-03 | intronic | *ANKRD27* | . | . | . |  |
| 19 | rs12981915 | 46,389,880 | C | T | 0.05 | 0.03 | upstream | *IRF2BP1* | dist=481 | . | . |  |
| 19 | rs183893 | 51,659,673 | C | T | 0.03 | 0.03 | ncRNA_exonic | *LOC101928517* | . | . | . |  |
| 19 | rs1053817 | 56,089,219 | T | C | 0.02 | 3.49E-03 | UTR3 | *ZNF579* | NM_152600:c.*98A>G | . | . |  |
| 19 | rs310464 | 56,124,049 | G | T | 5.38E-03 | 0.02 | upstream | *ZNF865* | dist=910 | . | . |  |
| 20 | rs1803415 | 1,099,523 | T | G | 0.04 | 0.04 | exonic | *PSMF1* | . | nonsynonymous SNV | PSMF1:NM_001323408:exon1:c.T107G:p.F36C,PSMF1:NM_001323409:exon1:c.T107G:p.F36C,PSMF1:NM_001323410:exon1:c.T107G:p.F36C,PSMF1:NM_006814:exon1:c.T107G:p.F36C,PSMF1:NM_178578:exon2:c.T107G:p.F36C |  |
| 20 | rs1535067 | 8,607,333 | T | C | 0.02 | 0.02 | intronic | *PLCB1* | . | . | . |  |
| 20 | rs4419296 | 8,607,426 | G | T | 0.01 | 0.02 | intronic | *PLCB1* | . | . | . |  |
| 20 | rs6055957 | 8,611,319 | A | C | 0.04 | 0.02 | intronic | *PLCB1* | . | . | . |  |
| 20 | rs6086595 | 8,764,779 | T | C | 0.05 | 0.02 | intronic | *PLCB1* | . | . | . |  |
| 20 | rs6131640 | 15,102,526 | C | T | 2.62E-05 | 0.01 | intronic | *MACROD2* | . | . | . |  |
| 20 | rs6035051 | 18,445,963 | A | G | 0.05 | 7.04E-04 | exonic | *DZANK1* | . | synonymous SNV | DZANK1:NM_001099407:exon2:c.T40C:p.L14L,DZANK1:NM_001351683:exon2:c.T67C:p.L23L,DZANK1:NM_001351684:exon2:c.T40C:p.L14L,DZANK1:NM_001367611:exon2:c.T40C:p.L14L,DZANK1:NM_001367612:exon2:c.T40C:p.L14L,DZANK1:NM_001367613:exon2:c.T40C:p.L14L,DZANK1:NM_001367614:exon2:c.T40C:p.L14L,DZANK1:NM_001367617:exon2:c.T40C:p.L14L,DZANK1:NM_001367618:exon2:c.T40C:p.L14L |  |
| 20 | rs3762199 | 18,487,792 | G | A | 0.04 | 0.04 | upstream | *SEC23B* | dist=334 | . | . |  |
| 20 | rs6081232 | 18,571,572 | G | A | 0.03 | 2.27E-04 | intronic | *DTD1* | . | . | . |  |
| 20 | rs2180601 | 18,636,022 | T | C | 0.05 | 9.75E-04 | intronic | *DTD1* | . | . | . |  |
| 20 | rs6081286 | 18,652,535 | A | G | 0.03 | 1.54E-04 | intronic | *DTD1* | . | . | . |  |
| 20 | rs6132097 | 18,657,601 | T | C | 0.04 | 1.26E-04 | ncRNA_intronic | *DTD1-AS1* | . | . | . |  |
| 20 | rs6136466 | 18,659,537 | T | C | 0.04 | 1.14E-04 | ncRNA_exonic | *DTD1-AS1* | . | . | . |  |
| 20 | rs6136469 | 18,661,704 | C | T | 0.05 | 1.84E-04 | ncRNA_intronic | *DTD1-AS1* | . | . | . |  |
| 20 | rs6081299 | 18,666,258 | G | A | 0.04 | 1.37E-04 | ncRNA_intronic | *DTD1-AS1* | . | . | . |  |
| 20 | rs6136485 | 18,693,841 | C | T | 0.02 | 1.18E-04 | intronic | *DTD1* | . | . | . |  |
| 20 | rs6136490 | 18,697,455 | G | A | 0.02 | 9.46E-05 | intronic | *DTD1* | . | . | . |  |
| 20 | rs7265126 | 18,697,665 | C | T | 0.02 | 1.00E-04 | intronic | *DTD1* | . | . | . |  |
| 20 | rs221985 | 31,669,544 | A | G | 0.04 | 8.12E-03 | intronic | *BPIFB4* | . | . | . |  |
| 20 | rs3848726 | 44,666,595 | T | G | 0.04 | 0.02 | intronic | *SLC12A5* | . | . | . |  |
| 20 | rs6021437 | 50,407,162 | C | T | 0.03 | 5.63E-03 | exonic | *SALL4* | . | synonymous SNV | SALL4:NM_020436:exon2:c.A1860A:p.T620T |  |
| 20 | rs4810120 | 56,732,801 | A | G | 0.03 | 0.02 | intronic | *C20orf85* | . | . | . |  |
| 20 | rs6070360 | 56,733,844 | C | T | 0.05 | 0.02 | intronic | *C20orf85* | . | . | . |  |
| 20 | rs3195701 | 61,288,355 | T | G | 3.04E-05 | 0.02 | exonic | *SLCO4A1* | . | synonymous SNV | SLCO4A1:NM_016354:exon2:c.G549G:p.G183G |  |
| 21 | rs760348 | 15,940,552 | T | C | 4.40E-03 | 0.02 | intronic | *SAMSN1* | . | . | . |  |
| 21 | rs2832760 | 31,662,152 | T | G | 0.04 | 0.02 | upstream | *KRTAP25-1* | dist=320 | . | . |  |
| 21 | rs2835186 | 37,338,139 | C | T | 0.03 | 0.02 | ncRNA_intronic | *LOC101928269* | . | . | . |  |
| 21 | rs2835223 | 37,369,811 | A | G | 0.02 | 0.04 | ncRNA_intronic | *LOC101928269* | . | . | . |  |
| 21 | rs2835226 | 37,372,684 | T | C | 0.03 | 0.03 | ncRNA_intronic | *LOC101928269* | . | . | . |  |
| 22 | rs5748469 | 19,907,099 | A | C | 0.04 | 0.02 | exonic | *TXNRD2* | . | synonymous SNV | TXNRD2:NM_001282512:exon3:c.G196G:p.A66A,TXNRD2:NM_001352303:exon3:c.G100G:p.A34A |  |
| 22 | rs2267062 | 24,544,482 | T | G | 0.05 | 0.01 | intronic | *CABIN1* | . | . | . |  |
| 22 | rs5760345 | 24,723,644 | T | C | 0.02 | 9.60E-03 | ncRNA_intronic | *SPECC1L-ADORA2A* | . | . | . |  |
| 22 | rs5760364 | 24,755,025 | T | G | 0.02 | 5.57E-03 | ncRNA_intronic | *SPECC1L-ADORA2A* | . | . | . |  |
| 22 | rs7678 | 24,813,165 | T | G | 0.02 | 4.78E-03 | ncRNA_intronic | *SPECC1L-ADORA2A* | . | . | . |  |
| 22 | rs16992986 | 34,157,526 | A | G | 0.05 | 0.02 | UTR5 | *LARGE1* | NM_001362951:c.-63T>C;NM_001362949:c.-63T>C;NM_001362953:c.-63T>C;NM_133642:c.-63T>C;NM_004737:c.-63T>C | . | . |  |
| 22 | rs11704316 | 37,634,852 | A | G | 7.26E-03 | 0.03 | intronic | *RAC2* | . | . | . |  |
| 22 | rs742152 | 37,896,749 | T | C | 6.43E-03 | 4.92E-03 | intronic | *CARD10* | . | . | . |  |
| 22 | rs4822021 | 41,726,053 | A | G | 0.02 | 1.70E-03 | exonic | *ZC3H7B* | . | synonymous SNV | ZC3H7B:NM_017590:exon6:c.G471G:p.Q157Q |  |
| 22 | rs2143695 | 41,728,620 | T | C | 0.05 | 2.76E-03 | intronic | *ZC3H7B* | . | . | . |  |
| 22 | rs8137373 | 41,729,216 | A | G | 0.03 | 1.77E-03 | intronic | *ZC3H7B* | . | . | . |  |
| 22 | rs9611577 | 41,793,090 | G | T | 0.01 | 6.32E-05 | UTR3 | *TEF* | NM_001145398:c.*1126G>T;NM_003216:c.*1126G>T | . | . |  |
| 22 | rs9626518 | 45,078,496 | A | G | 0.02 | 0.01 | intronic | *PRR5* | . | . | . |  |
| 22 | rs9626521 | 45,078,601 | C | T | 0.03 | 7.70E-03 | intronic | *PRR5* | . | . | . |  |
| 22 | rs5770911 | 51,011,241 | T | C | 0.04 | 0.05 | ncRNA_intronic | *CHKB-CPT1B* | . | . | . |  |
| 22 | rs3213445 | 51,015,838 | C | T | 0.01 | 0.03 | exonic | *CPT1B* | . | synonymous SNV | CPT1B:NM_001145137:exon2:c.A196A:p.I66I,CPT1B:NM_001145134:exon3:c.A196A:p.I66I,CPT1B:NM_001145135:exon3:c.A196A:p.I66I,CPT1B:NM_004377:exon3:c.A196A:p.I66I,CPT1B:NM_152245:exon3:c.A196A:p.I66I,CPT1B:NM_152246:exon3:c.A196A:p.I66I |  |

Note: Based on NCBI RefSeq GRCh37. Not in IB: Not included in PRS calculation in Indiana Biobank sample.
