# Supplementary material for: Gene-based polygenic risk scores analysis of alcohol use disorder in African Americans
Source: Transl Psychiatry. 2022 Jul 5;12:266. doi: 10.1038/s41398-022-02029-2 (PMC9256707; doi:10.1038/s41398-022-02029-2)
Supplement: Supplementary file 2 — 410 genes included in calculating PRSgene. [file 41398_2022_2029_MOESM2_ESM.docx]

**Table S2**: 410 genes included in calculating PRS_gene_. “y” in the second column means the gene is reported by previous GWAS of alcohol related phenotypes.

| Gene | reported in Alcohol GWAS |
| --- | --- |
| *AACS* |  |
| *ABL2* |  |
| *ACAP2* |  |
| *ACSM2B* |  |
| *ADAMTS3* |  |
| *ADCY10* |  |
| *ADCY7* |  |
| *ADGRA2* |  |
| *ADGRL3* |  |
| *ADH1B* | y |
| *ADH1C* | y |
| *ADH4* | y |
| *ADH5* | y |
| *ADHFE1* |  |
| *AGBL2* | y |
| *AK8* |  |
| *ALDH18A1* |  |
| *ALKBH5* |  |
| *ALPL* |  |
| *ANKK1* | y |
| *ANKRD10* |  |
| *ANKRD27* |  |
| *APH1B* |  |
| *ARIH2* |  |
| *ASB18* |  |
| *ASIC2* |  |
| *ATP2B1* | y |
| *ATP6V1C1* |  |
| *ATP6V1G2-DDX39B* |  |
| *ATXN1* |  |
| *ATXN7L2* |  |
| *AXIN1* |  |
| *BACH2* |  |
| *BANK1* | y |
| *BBS9* |  |
| *BDNF-AS* |  |
| *BPIFB4* |  |
| *BRD7* |  |
| *BRMS1* |  |
| *BRPF1* |  |
| *C20orf85* |  |
| *C2orf74* |  |
| *CABIN1* |  |
| *CADM1* |  |
| *CADPS2* |  |
| *CALN1* |  |
| *CAMK2G* |  |
| *CARD10* |  |
| *CCDC83* |  |
| *CDCP1* |  |
| *CDH12* |  |
| *CDH18* |  |
| *CDH7* |  |
| *CHAT* |  |
| *CHD8* |  |
| *CHKB-CPT1B* |  |
| *CHST10* |  |
| *CLYBL* |  |
| *CMSS1* |  |
| *CNOT4* |  |
| *CNOT7* |  |
| *CNPY3* |  |
| *CNPY3-GNMT* |  |
| *CNTLN* |  |
| *CNTN2* |  |
| *CNTN4* |  |
| *COL21A1* |  |
| *COL23A1* |  |
| *COL8A1* |  |
| *CPT1B* |  |
| *CSMD1* | y |
| *CTTN* |  |
| *DCC* | y |
| *DDA1* |  |
| *DENND1A* |  |
| *DENND3* |  |
| *DESI2* |  |
| *DLG2* |  |
| *DNAH11* | y |
| *DNAH6* |  |
| *DNAJC3* |  |
| *DRD2* | y |
| *DSC3* |  |
| *DTD1* | y |
| *DTD1-AS1* |  |
| *DUSP16* |  |
| *DZANK1* |  |
| *EEF1AKMT4* |  |
| *EFNA5* |  |
| *EGF* |  |
| *EHBP1* | y |
| *EIF3H* |  |
| *EIF4E* | y |
| *ELAVL2* |  |
| *ELL* |  |
| *ELMO1* |  |
| *ELOVL7* |  |
| *ENTPD1-AS1* |  |
| *EPHA7* |  |
| *EPHB1* |  |
| *ERBB4* |  |
| *ERC2* | y |
| *ERCC8* |  |
| *ERI3* |  |
| *ERMARD* |  |
| *ESR1* | y |
| *EYS* | y |
| *F11R* |  |
| *FAM49B* |  |
| *FANCL* |  |
| *FBN2* |  |
| *FBXO40* | y |
| *FGFR1* |  |
| *FIBCD1* |  |
| *FILIP1L* |  |
| *FLI1* |  |
| *FNBP4* | y |
| *FNDC3B* |  |
| *FSTL4* |  |
| *FTO* | y |
| *GAB1* |  |
| *GABRG3* |  |
| *GALNT10* | y |
| *GALNTL6* |  |
| *GBE1* |  |
| *GEMIN4* |  |
| *GIGYF2* |  |
| *GJA3* |  |
| *GLIS3* |  |
| *GNG11* |  |
| *GNMT* |  |
| *GPANK1* |  |
| *GPR176* |  |
| *GRID2* |  |
| *GRIN2B* |  |
| *GRM5* |  |
| *GRM7* |  |
| *GTF2H1* |  |
| *HACE1* |  |
| *HEATR3* |  |
| *HELZ* |  |
| *HHLA2* |  |
| *HIVEP2* |  |
| *HLA-C* |  |
| *HNF1B* |  |
| *HOMER1* |  |
| *HS6ST3* |  |
| *IL1RAP* |  |
| *ILF3* |  |
| *INSYN1* |  |
| *IP6K3* |  |
| *IRF2BP1* |  |
| *ITPR3* |  |
| *JCAD* | y |
| *KC6* |  |
| *KCNJ13* |  |
| *KCNMB2-AS1* |  |
| *KDM4B* |  |
| *KIAA0319* |  |
| *ELAPOR2* |  |
| *KIAA1549L* |  |
| *KIAA2012-AS1* |  |
| *KLF17* |  |
| *KRI1* |  |
| *KRT78* |  |
| *KRTAP25-1* |  |
| *KSR2* |  |
| *LAD1* |  |
| *LARGE1* |  |
| *LARS2* |  |
| *LARS2-AS1* |  |
| *LEF1* |  |
| *LEF1-AS1* |  |
| *LIFR* |  |
| *LIMA1* |  |
| *LINC00239* |  |
| *LINC00376* |  |
| *LINC00382* |  |
| *LINC01104* |  |
| *LINC01192* |  |
| *LINC01360* |  |
| *LINC01484* |  |
| *LINC01508* |  |
| *LINC01934* |  |
| *LINC01935* |  |
| *LINC01951* |  |
| *LINC02141* |  |
| *LINC02160* |  |
| *LINC02210-CRHR1* |  |
| *LINC02262* |  |
| *LINC02478* |  |
| *LINCR-0001* |  |
| *LINGO2* | y |
| *LMCD1* |  |
| *LOC100507053* | y |
| *LOC101927188* |  |
| *LOC101927948* |  |
| *LOC101927967* |  |
| *LOC101928269* |  |
| *LOC101928516* |  |
| *LOC101928517* |  |
| *LOC101928782* |  |
| *LOC101928896* |  |
| *LOC101928911* |  |
| *LOC101929415* |  |
| *LOC101929710* |  |
| *LOC105371703* |  |
| *LOC105374313* |  |
| *LOC105375713* |  |
| *LOC339975* |  |
| *LOC642484* |  |
| *LOC643711* |  |
| *LPP* |  |
| *LRP2* |  |
| *LSAMP* |  |
| *LY6G5B* |  |
| *LYRM4-AS1* |  |
| *MACROD1* | y |
| *MACROD2* |  |
| *MAGI2* |  |
| *MAN1A2* |  |
| *MAN1B1* | y |
| *MAP1S* |  |
| *MAPT* | y |
| *MCMBP* |  |
| *MED12L* |  |
| *METAP1* | y |
| *METTL15* |  |
| *MFGE8* |  |
| *MICB-DT* |  |
| *MIR100HG* |  |
| *MIR548N* |  |
| *MKRN2* |  |
| *MLN* |  |
| *MMP2-AS1* |  |
| *MSRA* | y |
| *MTHFR* | y |
| *MTTP* |  |
| *MYH15* |  |
| *MYO15A* |  |
| *NARS2* |  |
| *NAV2* |  |
| *NEK7* |  |
| *NFAT5* |  |
| *NFATC1* |  |
| *NFIB* |  |
| *NKAIN2* |  |
| *NKAIN3* |  |
| *NLGN1* |  |
| *NMNAT2* |  |
| *NRXN2* | y |
| *NTM* |  |
| *NTN1* |  |
| *OPCML* | y |
| *OXR1* |  |
| *P2RY12* |  |
| *P4HTM* |  |
| *PBX1* |  |
| *PCMTD1* |  |
| *PCSK6* |  |
| *PDCD1LG2* |  |
| *PDE4B* | y |
| *PDE4B-AS1* |  |
| *PDXDC2P-NPIPB14P* |  |
| *PEX6* |  |
| *PHYKPL* |  |
| *PIBF1* |  |
| *PIEZO1* |  |
| *PITX2* |  |
| *PKHD1L1* |  |
| *PKN2* |  |
| *PLCB1* |  |
| *PLD5* |  |
| *PLXNA2* | y |
| *PNPO* |  |
| *PODXL* | y |
| *POLRMT* |  |
| *PRDM14* |  |
| *PREP* |  |
| *PREPL* |  |
| *PRKAR2A* |  |
| *PRKCI* |  |
| *PRKN* |  |
| *PRR5* |  |
| *PRRT1* |  |
| *PSMF1* |  |
| *PSORS1C1* |  |
| *PTGFRN* |  |
| *PTPDC1* |  |
| *PTPRC* |  |
| *PTPRF* |  |
| *PTPRG* |  |
| *PXDNL* |  |
| *RAC2* |  |
| *RAD9A* |  |
| *RARS2* | y |
| *RBFOX1* | y |
| *RGL1* |  |
| *RGS4* |  |
| *RGS6* |  |
| *RHNO1* |  |
| *RNF5* |  |
| *RPL24* |  |
| *RPS6KA5* | y |
| *RRP12* |  |
| *RSAD2* |  |
| *RSRC1* |  |
| *SALL1* |  |
| *SALL4* |  |
| *SAMSN1* |  |
| *SCN2A* |  |
| *SEC23B* |  |
| *SERINC5* |  |
| *SFMBT2* |  |
| *SLC12A5* |  |
| *SLC25A37* |  |
| *SLC2A13* |  |
| *SLC39A8* | y |
| *SLC44A1* |  |
| *SLC4A10* |  |
| *SLC5A6* |  |
| *SLC6A9* |  |
| *SLC9A9* |  |
| *SLCO4A1* |  |
| *SLCO6A1* |  |
| *SMIM15-AS1* |  |
| *SMYD3* |  |
| *SNX29* |  |
| *SNX6* |  |
| *SORCS1* |  |
| *SORT1* |  |
| *SP4* |  |
| *SPATA17* |  |
| *SPATA22* |  |
| *SPECC1L-ADORA2A* |  |
| *SPHKAP* |  |
| *SPON1* |  |
| *SPRED2* |  |
| *SSBP4* |  |
| *ST6GALNAC3* |  |
| *STAB2* |  |
| *STAT4* |  |
| *STK39* |  |
| *STON1* |  |
| *TBC1D1* |  |
| *TCF7L1* |  |
| *TCTN3* |  |
| *TEF* |  |
| *TENM2* | y |
| *TEP1* |  |
| *TEX41* |  |
| *TLCD3A* |  |
| *TMEM108* |  |
| *TMEM132B* |  |
| *TMEM17* |  |
| *TMEM170B* |  |
| *TMEM178B* |  |
| *TMEM225* |  |
| *TMEM229B* |  |
| *TMEM260* |  |
| *TMEM68* |  |
| *TMPRSS5* |  |
| *TNKS* | y |
| *TNN* |  |
| *TNRC6A* | y |
| *TNXB* |  |
| *TRAPPC9* |  |
| *TRPC7* |  |
| *TRPM6* |  |
| *TSHZ3* |  |
| *TSNARE1* |  |
| *TSPAN15* |  |
| *TSPAN5* | y |
| *TTC12* |  |
| *TTN* |  |
| *TXNRD2* |  |
| *UGGT1* |  |
| *UGGT2* |  |
| *UMAD1* |  |
| *UNC79* |  |
| *UQCC2* |  |
| *USF1* |  |
| *USH2A* |  |
| *USP15* |  |
| *USP34* |  |
| *VEGFB* |  |
| *VPS37A* |  |
| *VPS53* |  |
| *VRK2* | y |
| *WARS2-AS1* |  |
| *WDPCP* |  |
| *WDR27* |  |
| *WDR7* | y |
| *WWC1* |  |
| *WWP2* |  |
| *XKR4* |  |
| *YME1L1* |  |
| *ZBTB16* |  |
| *ZBTB37* |  |
| *ZC3H13* |  |
| *ZC3H7B* |  |
| *ZFPM2* |  |
| *ZMAT4* |  |
| *ZNF462* | y |
| *ZNF579* |  |
| *ZNF804A* |  |
| *ZNF804B* |  |
| *ZNF865* |  |
| *ZSWIM8* |  |
| *ZW10* |  |
