# Supplementary material for: Gene-based polygenic risk scores analysis of alcohol use disorder in African Americans
Source: Transl Psychiatry. 2022 Jul 5;12:266. doi: 10.1038/s41398-022-02029-2 (PMC9256707; doi:10.1038/s41398-022-02029-2)
Supplement: Supplementary file 3 — 675 intergenic concordant variants. [file 41398_2022_2029_MOESM3_ESM.docx]

**Table S3**: 675 intergenic concordant variants.

| chr | SNP | bp | Allele1 | Allele2 | AA.Pvalue | EA.Pvalue | Func.refGene | Nearest genes | Distance to nearest genes | Not in IB |
| --- | --- | --- | --- | --- | --- | --- | --- | --- | --- | --- |
| 1 | rs2811626 | 37,880,752 | C | T | 0.05 | 1.89E-04 | intergenic | MIR4255;LINC01137 | dist=253517;dist=39728 |  |
| 1 | rs12131166 | 48,033,804 | G | A | 0.02 | 6.30E-03 | intergenic | FOXD2;TRABD2B | dist=127441;dist=192396 |  |
| 1 | rs3101338 | 72,750,353 | C | A | 0.04 | 2.48E-03 | intergenic | NEGR1;LINC01360 | dist=2131;dist=1021500 |  |
| 1 | rs11210201 | 73,766,431 | T | G | 0.02 | 9.15E-07 | intergenic | NEGR1;LINC01360 | dist=1018209;dist=5422 |  |
| 1 | rs2208565 | 73,810,229 | A | G | 0.03 | 8.40E-07 | intergenic | LINC01360;LINC02238 | dist=5669;dist=330133 |  |
| 1 | rs10789368 | 73,814,159 | G | A | 0.03 | 2.25E-05 | intergenic | LINC01360;LINC02238 | dist=9599;dist=326203 |  |
| 1 | rs1923212 | 73,814,973 | C | A | 0.03 | 7.45E-07 | intergenic | LINC01360;LINC02238 | dist=10413;dist=325389 |  |
| 1 | rs2340400 | 73,821,281 | C | T | 0.02 | 7.17E-07 | intergenic | LINC01360;LINC02238 | dist=16721;dist=319081 |  |
| 1 | rs10890034 | 73,824,279 | A | G | 0.03 | 6.98E-07 | intergenic | LINC01360;LINC02238 | dist=19719;dist=316083 |  |
| 1 | rs10789369 | 73,824,909 | A | G | 0.04 | 7.37E-07 | intergenic | LINC01360;LINC02238 | dist=20349;dist=315453 |  |
| 1 | rs11210215 | 73,834,346 | T | C | 0.04 | 6.56E-07 | intergenic | LINC01360;LINC02238 | dist=29786;dist=306016 |  |
| 1 | rs1572669 | 73,863,862 | A | G | 0.03 | 3.13E-06 | intergenic | LINC01360;LINC02238 | dist=59302;dist=276500 |  |
| 1 | rs12035848 | 73,870,901 | A | G | 9.75E-03 | 5.61E-08 | intergenic | LINC01360;LINC02238 | dist=66341;dist=269461 |  |
| 1 | rs11210235 | 73,878,194 | C | T | 0.02 | 6.85E-07 | intergenic | LINC01360;LINC02238 | dist=73634;dist=262168 |  |
| 1 | rs10465868 | 73,880,654 | G | A | 0.03 | 5.51E-07 | intergenic | LINC01360;LINC02238 | dist=76094;dist=259708 |  |
| 1 | rs1475065 | 73,882,880 | A | G | 0.03 | 2.32E-06 | intergenic | LINC01360;LINC02238 | dist=78320;dist=257482 |  |
| 1 | rs696667 | 75,149,512 | A | G | 5.12E-03 | 0.03 | intergenic | ERICH3;CRYZ | dist=10036;dist=21660 |  |
| 1 | rs696668 | 75,149,922 | G | T | 4.94E-03 | 0.02 | intergenic | ERICH3;CRYZ | dist=10446;dist=21250 |  |
| 1 | rs696672 | 75,155,271 | A | G | 2.53E-03 | 0.02 | intergenic | ERICH3;CRYZ | dist=15795;dist=15901 |  |
| 1 | rs10801871 | 88,176,320 | G | A | 0.02 | 0.03 | intergenic | LINC01364;PKN2-AS1 | dist=338982;dist=826876 |  |
| 1 | rs12121005 | 88,179,317 | C | T | 2.56E-03 | 9.92E-04 | intergenic | LINC01364;PKN2-AS1 | dist=341979;dist=823879 |  |
| 1 | rs12134683 | 88,180,243 | T | C | 2.24E-03 | 8.87E-04 | intergenic | LINC01364;PKN2-AS1 | dist=342905;dist=822953 |  |
| 1 | rs12129640 | 88,183,213 | A | G | 5.78E-03 | 5.32E-04 | intergenic | LINC01364;PKN2-AS1 | dist=345875;dist=819983 |  |
| 1 | rs10922903 | 91,159,586 | C | T | 0.05 | 1.53E-03 | intergenic | SNORD3G;BARHL2 | dist=36066;dist=17510 |  |
| 1 | rs164992 | 91,517,927 | C | T | 0.02 | 0.02 | intergenic | ZNF644;HFM1 | dist=30115;dist=208396 |  |
| 1 | rs10874924 | 95,966,161 | C | T | 0.01 | 3.22E-03 | intergenic | LINC01761;LINC02607 | dist=21249;dist=9511 |  |
| 1 | rs6593684 | 96,239,862 | G | A | 0.02 | 0.03 | intergenic | LINC02607;LINC02790 | dist=258842;dist=217762 |  |
| 1 | rs7544736 | 97,164,343 | G | A | 8.65E-03 | 0.01 | intergenic | LINC01787;PTBP2 | dist=324662;dist=22818 |  |
| 1 | rs12073730 | 97,172,454 | T | G | 6.73E-03 | 0.01 | intergenic | LINC01787;PTBP2 | dist=332773;dist=14707 |  |
| 1 | rs13374397 | 99,802,789 | T | C | 0.03 | 0.04 | intergenic | PLPPR4;LINC01708 | dist=27651;dist=135187 |  |
| 1 | rs12023333 | 100,267,478 | C | T | 0.02 | 0.02 | intergenic | FRRS1;AGL | dist=35287;dist=48439 |  |
| 1 | rs1389384 | 100,274,761 | T | C | 0.02 | 0.02 | intergenic | FRRS1;AGL | dist=42570;dist=41156 |  |
| 1 | rs10785776 | 106,718,569 | A | C | 0.02 | 1.02E-03 | intergenic | LINC01677;LINC01661 | dist=147694;dist=642292 |  |
| 1 | rs12023928 | 106,720,998 | T | C | 0.01 | 1.17E-03 | intergenic | LINC01677;LINC01661 | dist=150123;dist=639863 |  |
| 1 | rs11184786 | 106,726,037 | T | C | 0.01 | 8.24E-04 | intergenic | LINC01677;LINC01661 | dist=155162;dist=634824 |  |
| 1 | rs2084641 | 106,726,665 | G | T | 7.00E-03 | 9.28E-04 | intergenic | LINC01677;LINC01661 | dist=155790;dist=634196 |  |
| 1 | rs12047929 | 106,742,281 | C | T | 0.04 | 0.02 | intergenic | LINC01677;LINC01661 | dist=171406;dist=618580 |  |
| 1 | rs951439 | 163,033,691 | T | C | 5.96E-03 | 0.03 | intergenic | CCDC190;RGS4 | dist=195086;dist=4705 |  |
| 1 | rs2842028 | 163,035,531 | C | A | 0.02 | 0.02 | intergenic | CCDC190;RGS4 | dist=196926;dist=2865 |  |
| 1 | rs6678136 | 163,037,317 | A | G | 0.01 | 9.76E-03 | intergenic | CCDC190;RGS4 | dist=198712;dist=1079 |  |
| 1 | rs1819768 | 165,169,159 | C | A | 0.04 | 3.59E-03 | intergenic | PBX1;LMX1A | dist=313875;dist=1945 |  |
| 1 | rs2205960 | 173,191,475 | T | G | 9.63E-03 | 0.04 | intergenic | TNFSF4;LOC100506023 | dist=15005;dist=12724 |  |
| 1 | rs2802471 | 187,337,603 | C | A | 0.02 | 3.95E-04 | intergenic | LINC01036;LINC01037 | dist=8227;dist=75157 |  |
| 1 | rs1285527 | 187,342,526 | A | C | 0.05 | 3.85E-04 | intergenic | LINC01036;LINC01037 | dist=13150;dist=70234 |  |
| 1 | rs1730734 | 189,958,874 | C | T | 0.03 | 1.73E-04 | intergenic | NONE;BRINP3 | dist=NONE;dist=107915 |  |
| 1 | rs1711762 | 189,959,106 | T | G | 0.03 | 1.64E-04 | intergenic | NONE;BRINP3 | dist=NONE;dist=107683 |  |
| 1 | rs10920441 | 190,042,706 | C | T | 0.02 | 2.04E-03 | intergenic | NONE;BRINP3 | dist=NONE;dist=24083 |  |
| 1 | rs10921070 | 192,016,893 | G | A | 0.03 | 2.76E-03 | intergenic | LINC01680;RGS18 | dist=819265;dist=110699 |  |
| 1 | rs7512748 | 208,710,349 | A | G | 0.03 | 0.03 | intergenic | PLXNA2;LINC01717 | dist=292620;dist=191684 |  |
| 1 | rs12752047 | 208,711,001 | T | C | 0.03 | 0.04 | intergenic | PLXNA2;LINC01717 | dist=293272;dist=191032 |  |
| 1 | rs7519350 | 208,712,599 | T | G | 0.03 | 0.03 | intergenic | PLXNA2;LINC01717 | dist=294870;dist=189434 |  |
| 1 | rs11120551 | 215,491,496 | T | C | 0.02 | 0.05 | intergenic | KCNK2;KCTD3 | dist=81063;dist=249151 |  |
| 1 | rs6678049 | 215,507,613 | T | G | 0.01 | 0.04 | intergenic | KCNK2;KCTD3 | dist=97180;dist=233034 |  |
| 1 | rs644850 | 229,141,933 | A | G | 8.59E-03 | 0.02 | intergenic | RHOU;MIR4454 | dist=259522;dist=168497 |  |
| 1 | rs1389971 | 233,982,024 | G | A | 0.02 | 5.16E-03 | intergenic | KCNK1;SLC35F3 | dist=173764;dist=58398 |  |
| 2 | rs1429237 | 5,958,774 | A | G | 0.01 | 0.04 | intergenic | LINC01810;SILC1 | dist=6046;dist=114045 |  |
| 2 | rs1366738 | 5,979,944 | T | C | 3.09E-03 | 0.05 | intergenic | LINC01810;SILC1 | dist=27216;dist=92875 |  |
| 2 | rs17387850 | 18,002,691 | G | A | 0.02 | 0.05 | intergenic | MSGN1;KCNS3 | dist=3626;dist=56423 |  |
| 2 | rs2577734 | 23,494,356 | A | C | 3.98E-03 | 0.01 | intergenic | LINC01884;KLHL29 | dist=733197;dist=113694 |  |
| 2 | rs4665827 | 26,389,999 | T | G | 0.03 | 0.04 | intergenic | RAB10;GAREM2 | dist=29676;dist=5958 |  |
| 2 | rs6713874 | 33,850,382 | A | G | 0.04 | 0.04 | intergenic | FAM98A;LINC01317 | dist=25953;dist=81571 |  |
| 2 | rs6732467 | 53,223,652 | C | T | 0.01 | 0.04 | intergenic | MIR4431;ASB3 | dist=293899;dist=673465 |  |
| 2 | rs4672236 | 53,385,229 | A | C | 0.03 | 0.02 | intergenic | MIR4431;ASB3 | dist=455476;dist=511888 |  |
| 2 | rs12479326 | 53,845,673 | A | G | 0.03 | 1.58E-03 | intergenic | MIR4431;ASB3 | dist=915920;dist=51444 |  |
| 2 | rs934278 | 56,070,352 | G | A | 7.37E-04 | 1.84E-03 | intergenic | PNPT1;EFEMP1 | dist=149373;dist=22750 |  |
| 2 | rs1156672 | 56,770,848 | T | C | 0.05 | 6.98E-04 | intergenic | CCDC85A;VRK2 | dist=157539;dist=1363938 |  |
| 2 | rs10168588 | 57,275,031 | T | C | 0.04 | 6.96E-03 | intergenic | CCDC85A;VRK2 | dist=661722;dist=859755 |  |
| 2 | rs2245273 | 57,765,321 | C | T | 6.25E-05 | 9.22E-04 | intergenic | CCDC85A;VRK2 | dist=1152012;dist=369465 |  |
| 2 | rs34652320 | 57,781,893 | G | A | 2.56E-04 | 0.02 | intergenic | CCDC85A;VRK2 | dist=1168584;dist=352893 |  |
| 2 | rs11125721 | 57,826,028 | A | G | 4.57E-05 | 0.03 | intergenic | CCDC85A;VRK2 | dist=1212719;dist=308758 |  |
| 2 | rs2125923 | 57,854,044 | C | T | 3.91E-03 | 0.05 | intergenic | CCDC85A;VRK2 | dist=1240735;dist=280742 |  |
| 2 | rs820780 | 57,902,621 | A | C | 4.23E-05 | 0.03 | intergenic | CCDC85A;VRK2 | dist=1289312;dist=232165 |  |
| 2 | rs2695616 | 57,908,194 | G | A | 2.83E-04 | 6.22E-03 | intergenic | CCDC85A;VRK2 | dist=1294885;dist=226592 |  |
| 2 | rs13012916 | 57,923,006 | G | T | 4.40E-04 | 1.63E-05 | intergenic | CCDC85A;VRK2 | dist=1309697;dist=211780 |  |
| 2 | rs13026414 | 57,934,055 | T | C | 1.99E-04 | 1.23E-03 | intergenic | CCDC85A;VRK2 | dist=1320746;dist=200731 |  |
| 2 | rs11885093 | 57,941,185 | T | C | 3.04E-03 | 0.01 | intergenic | CCDC85A;VRK2 | dist=1327876;dist=193601 |  |
| 2 | rs11682175 | 57,987,593 | C | T | 1.22E-03 | 6.75E-09 | intergenic | CCDC85A;VRK2 | dist=1374284;dist=147193 |  |
| 2 | rs1030321 | 60,135,937 | C | T | 0.02 | 0.02 | intergenic | LINC01793;MIR4432HG | dist=629402;dist=450414 |  |
| 2 | rs2194701 | 60,137,193 | T | C | 0.02 | 9.12E-03 | intergenic | LINC01793;MIR4432HG | dist=630658;dist=449158 |  |
| 2 | rs1559621 | 60,140,608 | C | T | 0.02 | 0.04 | intergenic | LINC01793;MIR4432HG | dist=634073;dist=445743 |  |
| 2 | rs13388903 | 62,726,068 | C | A | 9.08E-03 | 7.63E-04 | intergenic | B3GNT2;TMEM17 | dist=274202;dist=1286 |  |
| 2 | rs12614398 | 66,168,065 | G | A | 0.03 | 0.01 | intergenic | SPRED2;MIR4778 | dist=508332;dist=417316 |  |
| 2 | rs6546206 | 66,293,343 | A | G | 8.47E-04 | 0.01 | intergenic | SPRED2;MIR4778 | dist=633610;dist=292038 |  |
| 2 | rs6546207 | 66,293,382 | T | G | 9.38E-04 | 0.01 | intergenic | SPRED2;MIR4778 | dist=633649;dist=291999 |  |
| 2 | rs2170912 | 66,293,425 | T | C | 9.89E-04 | 0.01 | intergenic | SPRED2;MIR4778 | dist=633692;dist=291956 |  |
| 2 | rs7585877 | 66,296,174 | G | A | 1.44E-03 | 7.28E-03 | intergenic | SPRED2;MIR4778 | dist=636441;dist=289207 |  |
| 2 | rs6757906 | 68,354,727 | C | A | 0.04 | 9.59E-03 | intergenic | C1D;WDR92 | dist=64591;dist=2210 |  |
| 2 | rs4267542 | 69,231,050 | A | C | 4.19E-03 | 0.05 | intergenic | GKN1;ANTXR1 | dist=22938;dist=9226 |  |
| 2 | rs13035731 | 73,090,692 | C | T | 0.04 | 0.01 | intergenic | EXOC6B;SPR | dist=37530;dist=23845 |  |
| 2 | rs7573531 | 78,578,728 | T | G | 0.04 | 0.03 | intergenic | LOC101927967;LOC105374820 | dist=60871;dist=193929 |  |
| 2 | rs10200062 | 78,657,154 | T | C | 0.03 | 0.01 | intergenic | LOC101927967;LOC105374820 | dist=139297;dist=115503 |  |
| 2 | rs12467106 | 79,447,701 | A | G | 0.04 | 0.03 | intergenic | REG3A;CTNNA2-AS1 | dist=60822;dist=273141 |  |
| 2 | rs1434196 | 79,451,571 | T | C | 0.04 | 0.03 | intergenic | REG3A;CTNNA2-AS1 | dist=64692;dist=269271 |  |
| 2 | rs1030901 | 101,007,314 | G | A | 0.05 | 0.05 | intergenic | LONRF2;CHST10 | dist=68351;dist=1008 |  |
| 2 | rs6705957 | 120,176,240 | C | T | 0.02 | 0.03 | intergenic | DBI;TMEM37 | dist=46104;dist=13190 |  |
| 2 | rs2030745 | 121,309,282 | A | G | 0.04 | 7.31E-04 | intergenic | LINC01101;GLI2 | dist=85357;dist=184162 |  |
| 2 | rs2030746 | 121,309,488 | T | C | 0.05 | 4.07E-04 | intergenic | LINC01101;GLI2 | dist=85563;dist=183956 |  |
| 2 | rs1712842 | 122,819,883 | G | A | 0.02 | 0.05 | intergenic | TSN;LINC01826 | dist=294455;dist=1003849 |  |
| 2 | rs13408677 | 129,151,284 | A | G | 0.05 | 0.05 | intergenic | HS6ST1;LOC101927881 | dist=74842;dist=470890 |  |
| 2 | rs12469052 | 162,319,353 | C | T | 0.01 | 0.01 | intergenic | TBR1;AHCTF1P1 | dist=36972;dist=36225 |  |
| 2 | rs12467662 | 162,386,784 | A | G | 0.01 | 7.42E-03 | intergenic | AHCTF1P1;SLC4A10 | dist=22371;dist=94061 |  |
| 2 | rs2389549 | 162,462,209 | C | T | 0.01 | 0.02 | intergenic | AHCTF1P1;SLC4A10 | dist=97796;dist=18636 |  |
| 2 | rs1567420 | 162,475,601 | C | T | 6.38E-03 | 0.02 | intergenic | AHCTF1P1;SLC4A10 | dist=111188;dist=5244 |  |
| 2 | rs7595649 | 164,008,082 | T | C | 0.04 | 0.01 | intergenic | KCNH7;FIGN | dist=312805;dist=451040 |  |
| 2 | rs13395667 | 164,008,246 | T | C | 0.02 | 0.01 | intergenic | KCNH7;FIGN | dist=312969;dist=450876 |  |
| 2 | rs34238342 | 164,936,017 | C | T | 0.05 | 7.85E-03 | intergenic | FIGN;GRB14 | dist=343499;dist=412910 |  |
| 2 | rs6729039 | 169,106,850 | G | A | 0.02 | 4.52E-03 | intergenic | STK39;CERS6 | dist=2745;dist=205932 |  |
| 2 | rs16859405 | 172,345,151 | A | G | 0.04 | 0.03 | intergenic | DCAF17;CYBRD1 | dist=3589;dist=33592 |  |
| 2 | rs12466967 | 174,344,080 | C | A | 0.01 | 0.02 | intergenic | CDCA7;SP3 | dist=110359;dist=421425 |  |
| 2 | rs11684526 | 184,134,742 | G | A | 3.97E-04 | 1.75E-03 | intergenic | NUP35;MIR548AE1 | dist=108330;dist=1108960 |  |
| 2 | rs4274569 | 184,139,076 | C | T | 5.08E-04 | 1.61E-03 | intergenic | NUP35;MIR548AE1 | dist=112664;dist=1104626 |  |
| 2 | rs4143776 | 185,186,670 | T | C | 0.04 | 0.03 | intergenic | NUP35;MIR548AE1 | dist=1160258;dist=57032 |  |
| 2 | rs840616 | 188,196,469 | C | T | 0.05 | 0.02 | intergenic | ZSWIM2;CALCRL | dist=482572;dist=10223 |  |
| 2 | rs17241582 | 189,802,107 | G | A | 0.02 | 0.02 | intergenic | DIRC1;COL3A1 | dist=147257;dist=36992 |  |
| 2 | rs12472343 | 192,525,536 | T | C | 5.10E-03 | 6.39E-03 | intergenic | MYO1B;NABP1 | dist=235424;dist=17262 |  |
| 2 | rs10931654 | 195,657,969 | C | A | 0.05 | 0.05 | intergenic | LINC01790;SLC39A10 | dist=31810;dist=863713 |  |
| 2 | rs13034702 | 200,719,755 | A | G | 9.63E-03 | 4.43E-03 | intergenic | FTCDNL1;C2orf69 | dist=3859;dist=56261 |  |
| 2 | rs4611634 | 221,339,243 | A | G | 0.04 | 9.92E-04 | intergenic | MIR4268;EPHA4 | dist=567957;dist=943504 |  |
| 2 | rs1473618 | 224,170,756 | C | T | 0.04 | 0.03 | intergenic | KCNE4;SCG2 | dist=250399;dist=290902 |  |
| 2 | rs2894500 | 224,179,428 | G | A | 0.04 | 0.05 | intergenic | KCNE4;SCG2 | dist=259071;dist=282230 |  |
| 2 | rs12616293 | 226,741,320 | A | G | 6.14E-03 | 0.05 | intergenic | NYAP2;LOC646736 | dist=172950;dist=266190 |  |
| 2 | rs16866957 | 227,174,994 | C | A | 0.02 | 5.07E-04 | intergenic | LOC646736;MIR5702 | dist=130216;dist=348432 |  |
| 2 | rs6723142 | 229,081,848 | G | A | 0.04 | 0.02 | intergenic | SPHKAP;LINC01807 | dist=35445;dist=266131 | Y |
| 3 | rs1015456 | 107,776 | T | C | 0.02 | 0.05 | intergenic | LINC01986;CHL1-AS2 | dist=41601;dist=129665 |  |
| 3 | rs305522 | 12,015,313 | C | A | 0.02 | 3.23E-03 | intergenic | TAMM41;SYN2 | dist=126954;dist=30521 |  |
| 3 | rs305511 | 12,021,902 | T | C | 0.04 | 6.93E-03 | intergenic | TAMM41;SYN2 | dist=133543;dist=23932 |  |
| 3 | rs17044638 | 18,609,712 | G | A | 0.02 | 3.83E-03 | intergenic | SATB1-AS1;KCNH8 | dist=38106;dist=580290 |  |
| 3 | rs7640479 | 18,940,080 | A | G | 0.02 | 3.54E-03 | intergenic | SATB1-AS1;KCNH8 | dist=368474;dist=249922 |  |
| 3 | rs9813151 | 18,963,323 | A | G | 0.02 | 0.03 | intergenic | SATB1-AS1;KCNH8 | dist=391717;dist=226679 |  |
| 3 | rs2139172 | 34,288,960 | A | G | 0.01 | 0.02 | intergenic | PDCD6IP;LOC101928135 | dist=377761;dist=628329 |  |
| 3 | rs6798288 | 53,985,176 | C | A | 0.04 | 0.04 | intergenic | SELENOK;CACNA2D3 | dist=59290;dist=171403 |  |
| 3 | rs1427430 | 69,755,567 | A | C | 0.03 | 4.80E-03 | intergenic | FRMD4B;MITF | dist=320328;dist=33048 |  |
| 3 | rs17006389 | 69,766,732 | G | A | 0.04 | 5.87E-03 | intergenic | FRMD4B;MITF | dist=331493;dist=21883 |  |
| 3 | rs13062403 | 94,841,342 | A | G | 0.03 | 0.01 | intergenic | LINC00879;MTHFD2P1 | dist=131170;dist=531925 |  |
| 3 | rs6791696 | 101,409,096 | G | A | 6.00E-03 | 0.01 | intergenic | RPL24;PDCL3P4 | dist=3534;dist=22182 |  |
| 3 | rs2712494 | 106,280,822 | T | G | 0.04 | 0.02 | intergenic | CBLB;LINC00882 | dist=692577;dist=547815 |  |
| 3 | rs13089498 | 106,343,354 | A | G | 0.05 | 0.02 | intergenic | CBLB;LINC00882 | dist=755109;dist=485283 |  |
| 3 | rs2895315 | 106,393,843 | T | G | 0.05 | 9.60E-03 | intergenic | CBLB;LINC00882 | dist=805598;dist=434794 |  |
| 3 | rs1920028 | 108,884,070 | C | T | 0.04 | 0.04 | intergenic | C3orf85;LINC00488 | dist=13822;dist=12942 |  |
| 3 | rs1163351 | 108,888,506 | T | C | 0.04 | 0.04 | intergenic | C3orf85;LINC00488 | dist=18258;dist=8506 |  |
| 3 | rs1249892 | 112,495,168 | A | G | 0.04 | 0.01 | intergenic | LINC02042;CD200R1L-AS1 | dist=27002;dist=26157 |  |
| 3 | rs1249894 | 112,496,467 | T | C | 0.03 | 0.01 | intergenic | LINC02042;CD200R1L-AS1 | dist=28301;dist=24858 |  |
| 3 | rs1387019 | 112,500,272 | C | T | 0.02 | 0.03 | intergenic | LINC02042;CD200R1L-AS1 | dist=32106;dist=21053 |  |
| 3 | rs2688602 | 112,500,763 | A | G | 0.03 | 0.02 | intergenic | LINC02042;CD200R1L-AS1 | dist=32597;dist=20562 |  |
| 3 | rs9827082 | 112,501,840 | T | C | 0.02 | 0.02 | intergenic | LINC02042;CD200R1L-AS1 | dist=33674;dist=19485 |  |
| 3 | rs9870601 | 112,509,261 | C | T | 0.04 | 5.41E-03 | intergenic | LINC02042;CD200R1L-AS1 | dist=41095;dist=12064 |  |
| 3 | rs11923997 | 126,009,560 | T | G | 0.01 | 0.01 | intergenic | ALDH1L1-AS2;KLF15 | dist=80549;dist=51918 |  |
| 3 | rs6439381 | 132,736,382 | G | T | 0.02 | 0.05 | intergenic | NPHP3-AS1;TMEM108 | dist=143327;dist=20750 |  |
| 3 | rs1464403 | 132,751,699 | T | C | 0.02 | 0.05 | intergenic | NPHP3-AS1;TMEM108 | dist=158644;dist=5433 |  |
| 3 | rs4681387 | 147,610,496 | G | A | 0.05 | 0.02 | intergenic | LOC440982;LINC02032 | dist=382799;dist=185450 |  |
| 3 | rs11708569 | 149,709,361 | G | A | 0.05 | 3.69E-03 | intergenic | TMEM183B;LOC105374313 | dist=8208;dist=104869 |  |
| 3 | rs16824965 | 155,162,638 | C | T | 1.29E-03 | 1.49E-03 | intergenic | STRIT1;PLCH1 | dist=151166;dist=35032 |  |
| 3 | rs16824986 | 155,165,064 | T | C | 8.28E-04 | 3.20E-03 | intergenic | STRIT1;PLCH1 | dist=153592;dist=32606 |  |
| 3 | rs16824992 | 155,167,027 | G | T | 7.25E-04 | 3.55E-03 | intergenic | STRIT1;PLCH1 | dist=155555;dist=30643 |  |
| 3 | rs4635724 | 161,394,052 | C | T | 0.03 | 8.92E-03 | intergenic | OTOL1;LINC01192 | dist=172322;dist=1500979 |  |
| 3 | rs1399903 | 161,394,104 | C | T | 0.04 | 0.03 | intergenic | OTOL1;LINC01192 | dist=172374;dist=1500927 |  |
| 3 | rs9290133 | 162,482,732 | A | G | 0.02 | 3.08E-03 | intergenic | OTOL1;LINC01192 | dist=1261002;dist=412299 |  |
| 3 | rs1872975 | 162,489,771 | G | A | 0.02 | 1.62E-03 | intergenic | OTOL1;LINC01192 | dist=1268041;dist=405260 |  |
| 3 | rs206303 | 162,494,772 | A | G | 0.03 | 1.81E-03 | intergenic | OTOL1;LINC01192 | dist=1273042;dist=400259 |  |
| 3 | rs1586501 | 162,735,677 | A | C | 1.84E-03 | 0.04 | intergenic | OTOL1;LINC01192 | dist=1513947;dist=159354 |  |
| 3 | rs6784221 | 162,740,414 | T | G | 6.25E-03 | 6.83E-03 | intergenic | OTOL1;LINC01192 | dist=1518684;dist=154617 |  |
| 3 | rs6776806 | 162,773,253 | A | G | 6.17E-03 | 0.04 | intergenic | OTOL1;LINC01192 | dist=1551523;dist=121778 |  |
| 3 | rs7433186 | 162,782,165 | G | A | 0.01 | 3.35E-03 | intergenic | OTOL1;LINC01192 | dist=1560435;dist=112866 |  |
| 3 | rs843067 | 163,028,656 | C | T | 0.03 | 4.49E-03 | intergenic | LINC01192;MIR1263 | dist=7567;dist=860603 |  |
| 3 | rs3115054 | 163,029,836 | C | A | 0.04 | 4.34E-03 | intergenic | LINC01192;MIR1263 | dist=8747;dist=859423 |  |
| 3 | rs7637094 | 163,044,968 | G | A | 0.03 | 2.93E-03 | intergenic | LINC01192;MIR1263 | dist=23879;dist=844291 |  |
| 3 | rs6782192 | 163,056,421 | T | G | 0.04 | 4.11E-03 | intergenic | LINC01192;MIR1263 | dist=35332;dist=832838 |  |
| 3 | rs1593645 | 163,063,391 | C | T | 0.04 | 4.17E-03 | intergenic | LINC01192;MIR1263 | dist=42302;dist=825868 |  |
| 3 | rs1392340 | 181,659,359 | C | T | 3.24E-03 | 6.15E-03 | intergenic | SOX2-OT;LINC01206 | dist=199346;dist=10793 |  |
| 3 | rs9878418 | 190,539,107 | A | C | 0.05 | 0.03 | intergenic | IL1RAP;GMNC | dist=161568;dist=31419 |  |
| 3 | rs6799514 | 190,540,022 | C | T | 0.04 | 0.02 | intergenic | IL1RAP;GMNC | dist=162483;dist=30504 |  |
| 4 | rs6849099 | 12,803,879 | A | G | 0.02 | 3.23E-03 | intergenic | LINC02270;RAB28 | dist=550970;dist=565468 |  |
| 4 | rs4414946 | 18,624,806 | G | T | 0.03 | 0.03 | intergenic | LCORL;SLIT2 | dist=601308;dist=1628722 |  |
| 4 | rs4698236 | 18,629,248 | A | C | 0.04 | 0.03 | intergenic | LCORL;SLIT2 | dist=605750;dist=1624280 |  |
| 4 | rs1874288 | 18,640,524 | A | G | 0.03 | 0.02 | intergenic | LCORL;SLIT2 | dist=617026;dist=1613004 |  |
| 4 | rs7660232 | 28,232,617 | A | G | 0.03 | 1.37E-03 | intergenic | LINC02261;MIR4275 | dist=948770;dist=588587 |  |
| 4 | rs1497691 | 28,233,327 | T | C | 0.03 | 1.44E-03 | intergenic | LINC02261;MIR4275 | dist=949480;dist=587877 |  |
| 4 | rs4452420 | 28,233,491 | C | T | 0.03 | 1.44E-03 | intergenic | LINC02261;MIR4275 | dist=949644;dist=587713 |  |
| 4 | rs973586 | 28,234,938 | C | T | 0.02 | 1.29E-03 | intergenic | LINC02261;MIR4275 | dist=951091;dist=586266 |  |
| 4 | rs1523168 | 31,946,280 | T | C | 0.05 | 0.04 | intergenic | LINC02501;LINC02506 | dist=385837;dist=52721 |  |
| 4 | rs11938869 | 35,478,901 | G | A | 0.05 | 0.02 | intergenic | LINC02484;ARAP2 | dist=1207532;dist=588725 |  |
| 4 | rs13137019 | 35,512,060 | A | G | 0.05 | 0.01 | intergenic | LINC02484;ARAP2 | dist=1240691;dist=555566 |  |
| 4 | rs7694691 | 35,528,798 | T | C | 0.05 | 0.04 | intergenic | LINC02484;ARAP2 | dist=1257429;dist=538828 |  |
| 4 | rs1425470 | 35,535,809 | C | T | 0.04 | 0.05 | intergenic | LINC02484;ARAP2 | dist=1264440;dist=531817 |  |
| 4 | rs6843206 | 35,545,808 | G | A | 0.04 | 0.05 | intergenic | LINC02484;ARAP2 | dist=1274439;dist=521818 |  |
| 4 | rs1369177 | 43,739,772 | T | C | 0.04 | 0.05 | intergenic | LINC02383;LINC02475 | dist=245212;dist=279106 |  |
| 4 | rs10517081 | 43,741,109 | C | T | 0.04 | 0.05 | intergenic | LINC02383;LINC02475 | dist=246549;dist=277769 |  |
| 4 | rs4695120 | 43,743,961 | A | G | 0.05 | 0.04 | intergenic | LINC02383;LINC02475 | dist=249401;dist=274917 |  |
| 4 | rs1512315 | 45,105,210 | T | C | 0.03 | 0.04 | intergenic | GNPDA2;GABRG1 | dist=376559;dist=932576 |  |
| 4 | rs218280 | 55,424,289 | T | G | 0.04 | 0.04 | intergenic | LINC02283;LINC02260 | dist=202018;dist=45089 |  |
| 4 | rs218282 | 55,424,655 | A | G | 0.03 | 0.04 | intergenic | LINC02283;LINC02260 | dist=202384;dist=44723 |  |
| 4 | rs9312704 | 59,611,299 | T | C | 2.55E-03 | 1.25E-03 | intergenic | LINC02494;LINC02429 | dist=208806;dist=238701 |  |
| 4 | rs13435742 | 59,618,003 | C | T | 2.21E-03 | 1.14E-03 | intergenic | LINC02494;LINC02429 | dist=215510;dist=231997 |  |
| 4 | rs2048462 | 60,306,500 | C | T | 0.01 | 0.01 | intergenic | LINC02429;MIR548AG1 | dist=393823;dist=1481837 |  |
| 4 | rs4860567 | 60,314,238 | A | G | 0.02 | 0.02 | intergenic | LINC02429;MIR548AG1 | dist=401561;dist=1474099 |  |
| 4 | rs6826593 | 60,314,905 | C | T | 0.02 | 0.02 | intergenic | LINC02429;MIR548AG1 | dist=402228;dist=1473432 |  |
| 4 | rs17218618 | 60,399,836 | G | A | 0.03 | 0.02 | intergenic | LINC02429;MIR548AG1 | dist=487159;dist=1388501 |  |
| 4 | rs4411951 | 73,445,144 | T | C | 0.04 | 0.01 | intergenic | ADAMTS3;COX18 | dist=10206;dist=472935 |  |
| 4 | rs4694496 | 73,445,895 | A | G | 0.04 | 0.01 | intergenic | ADAMTS3;COX18 | dist=10957;dist=472184 |  |
| 4 | rs10938029 | 73,450,900 | G | T | 0.04 | 0.01 | intergenic | ADAMTS3;COX18 | dist=15962;dist=467179 |  |
| 4 | rs3923442 | 88,189,733 | A | G | 0.03 | 0.04 | intergenic | KLHL8;MIR5705 | dist=47963;dist=31914 |  |
| 4 | rs4516724 | 98,072,050 | T | C | 0.02 | 0.04 | intergenic | LINC02267;STPG2-AS1 | dist=332484;dist=216027 |  |
| 4 | rs4699308 | 98,078,295 | C | T | 0.02 | 0.04 | intergenic | LINC02267;STPG2-AS1 | dist=338729;dist=209782 |  |
| 4 | rs4540053 | 98,094,566 | G | A | 0.01 | 0.05 | intergenic | LINC02267;STPG2-AS1 | dist=355000;dist=193511 |  |
| 4 | rs1013158 | 98,253,309 | C | T | 8.32E-03 | 0.04 | intergenic | LINC02267;STPG2-AS1 | dist=513743;dist=34768 |  |
| 4 | rs4699667 | 99,661,738 | T | C | 0.04 | 4.42E-03 | intergenic | LOC105377342;EIF4E | dist=76036;dist=131097 |  |
| 4 | rs1881157 | 99,712,077 | A | G | 8.09E-03 | 0.03 | intergenic | LOC105377342;EIF4E | dist=126375;dist=80758 |  |
| 4 | rs6843891 | 99,748,968 | G | T | 6.70E-03 | 1.95E-09 | intergenic | LOC105377342;EIF4E | dist=163266;dist=43867 | Y |
| 4 | rs4699680 | 99,759,132 | G | A | 9.24E-03 | 1.23E-09 | intergenic | LOC105377342;EIF4E | dist=173430;dist=33703 | Y |
| 4 | rs17511498 | 99,855,241 | G | A | 0.01 | 2.89E-05 | intergenic | EIF4E;METAP1 | dist=3453;dist=61631 |  |
| 4 | rs1230165 | 99,986,373 | G | A | 0.02 | 2.58E-03 | intergenic | METAP1;ADH5 | dist=2413;dist=5757 |  |
| 4 | rs1789891 | 100,250,419 | A | C | 3.30E-03 | 9.15E-13 | intergenic | ADH1B;ADH1C | dist=7861;dist=7235 |  |
| 4 | rs3846448 | 100,254,935 | A | G | 0.01 | 2.43E-05 | intergenic | ADH1B;ADH1C | dist=12377;dist=2719 |  |
| 4 | rs1229979 | 100,255,953 | T | C | 0.02 | 1.74E-06 | intergenic | ADH1B;ADH1C | dist=13395;dist=1701 |  |
| 4 | rs1229978 | 100,256,199 | C | T | 1.13E-04 | 1.50E-19 | intergenic | ADH1B;ADH1C | dist=13641;dist=1455 |  |
| 4 | rs1662037 | 100,278,819 | A | G | 0.01 | 2.55E-05 | intergenic | ADH1C;ADH7 | dist=4916;dist=54599 |  |
| 4 | rs1614377 | 100,279,332 | A | G | 0.01 | 5.45E-05 | intergenic | ADH1C;ADH7 | dist=5429;dist=54086 |  |
| 4 | rs2851300 | 100,279,824 | T | C | 9.09E-05 | 1.07E-17 | intergenic | ADH1C;ADH7 | dist=5921;dist=53594 |  |
| 4 | rs166892 | 100,282,765 | T | C | 2.53E-03 | 9.18E-05 | intergenic | ADH1C;ADH7 | dist=8862;dist=50653 |  |
| 4 | rs1154434 | 100,285,012 | A | G | 0.01 | 5.98E-05 | intergenic | ADH1C;ADH7 | dist=11109;dist=48406 |  |
| 4 | rs1154435 | 100,285,148 | A | G | 1.04E-04 | 1.04E-16 | intergenic | ADH1C;ADH7 | dist=11245;dist=48270 |  |
| 4 | rs1154436 | 100,285,351 | A | G | 0.01 | 5.63E-05 | intergenic | ADH1C;ADH7 | dist=11448;dist=48067 |  |
| 4 | rs2851293 | 100,307,378 | C | T | 0.02 | 5.97E-11 | intergenic | ADH1C;ADH7 | dist=33475;dist=26040 |  |
| 4 | rs284789 | 100,332,057 | G | A | 8.57E-04 | 3.68E-06 | intergenic | ADH1C;ADH7 | dist=58154;dist=1361 |  |
| 4 | rs10026337 | 101,197,518 | C | A | 0.01 | 1.76E-03 | intergenic | LOC101929353;SNORA101A | dist=60856;dist=118766 |  |
| 4 | rs2014244 | 101,742,394 | G | A | 0.04 | 2.26E-05 | intergenic | LINC01216;PPP3CA | dist=146124;dist=202181 |  |
| 4 | rs17031254 | 102,325,626 | T | G | 0.02 | 0.03 | intergenic | FLJ20021;BANK1 | dist=55586;dist=386138 |  |
| 4 | rs2162339 | 102,352,222 | G | A | 0.03 | 0.03 | intergenic | FLJ20021;BANK1 | dist=82182;dist=359542 |  |
| 4 | rs7679597 | 102,576,776 | T | C | 0.02 | 0.01 | intergenic | FLJ20021;BANK1 | dist=306736;dist=134988 |  |
| 4 | rs7675302 | 102,998,681 | T | G | 0.05 | 6.40E-04 | intergenic | BANK1;SLC39A8 | dist=2712;dist=173517 |  |
| 4 | rs151414 | 103,137,879 | A | G | 0.01 | 9.49E-03 | intergenic | BANK1;SLC39A8 | dist=141910;dist=34319 |  |
| 4 | rs151413 | 103,137,941 | G | T | 0.01 | 0.01 | intergenic | BANK1;SLC39A8 | dist=141972;dist=34257 |  |
| 4 | rs1540052 | 103,137,977 | G | A | 5.94E-03 | 3.42E-03 | intergenic | BANK1;SLC39A8 | dist=142008;dist=34221 |  |
| 4 | rs2723286 | 111,583,334 | T | C | 0.02 | 0.04 | intergenic | PITX2;MIR297 | dist=20055;dist=198404 |  |
| 4 | rs11733579 | 136,919,049 | T | C | 0.04 | 0.02 | intergenic | LINC00613;LINC02511 | dist=84214;dist=798008 |  |
| 4 | rs1526232 | 138,638,127 | A | G | 0.02 | 0.02 | intergenic | LINC02172;LINC00616 | dist=113543;dist=310450 |  |
| 4 | rs6835678 | 138,873,429 | C | T | 0.04 | 2.69E-04 | intergenic | LINC02172;LINC00616 | dist=348845;dist=75148 |  |
| 4 | rs7685800 | 138,874,841 | C | T | 0.03 | 2.69E-04 | intergenic | LINC02172;LINC00616 | dist=350257;dist=73736 |  |
| 4 | rs7667492 | 138,874,889 | G | T | 0.03 | 2.72E-04 | intergenic | LINC02172;LINC00616 | dist=350305;dist=73688 |  |
| 4 | rs7692121 | 138,875,762 | A | C | 0.01 | 1.74E-03 | intergenic | LINC02172;LINC00616 | dist=351178;dist=72815 |  |
| 4 | rs7680462 | 138,882,967 | A | G | 0.04 | 5.15E-04 | intergenic | LINC02172;LINC00616 | dist=358383;dist=65610 |  |
| 4 | rs13148340 | 138,883,185 | C | A | 0.02 | 1.56E-03 | intergenic | LINC02172;LINC00616 | dist=358601;dist=65392 |  |
| 4 | rs7657863 | 149,905,182 | A | C | 0.02 | 0.02 | intergenic | LOC105377480;LINC02355 | dist=41652;dist=170265 |  |
| 4 | rs996834 | 149,924,895 | T | C | 0.02 | 2.91E-03 | intergenic | LOC105377480;LINC02355 | dist=61365;dist=150552 |  |
| 4 | rs2520495 | 149,933,356 | A | G | 0.02 | 3.17E-03 | intergenic | LOC105377480;LINC02355 | dist=69826;dist=142091 |  |
| 4 | rs12511408 | 149,943,746 | T | C | 0.05 | 1.47E-03 | intergenic | LOC105377480;LINC02355 | dist=80216;dist=131701 |  |
| 4 | rs11099827 | 152,714,658 | C | T | 0.04 | 7.75E-03 | intergenic | GATB;LINC02273 | dist=32483;dist=307247 |  |
| 4 | rs7670700 | 168,985,670 | C | T | 4.11E-03 | 0.02 | intergenic | SPOCK3;ANXA10 | dist=829929;dist=28018 |  |
| 4 | rs11132008 | 168,987,754 | G | A | 3.89E-03 | 0.03 | intergenic | SPOCK3;ANXA10 | dist=832013;dist=25934 |  |
| 4 | rs17067290 | 180,186,551 | A | G | 0.03 | 0.04 | intergenic | LINC01098;LINC00290 | dist=1274647;dist=1798692 |  |
| 4 | rs12643739 | 180,365,721 | A | G | 0.02 | 0.04 | intergenic | LINC01098;LINC00290 | dist=1453817;dist=1619522 |  |
| 4 | rs17069252 | 181,271,508 | T | C | 0.02 | 0.01 | intergenic | NONE;LINC00290 | dist=NONE;dist=713735 |  |
| 4 | rs2309470 | 182,222,033 | C | T | 0.01 | 0.04 | intergenic | LINC02500;TEMN3-AS1 | dist=35851;dist=519125 |  |
| 4 | rs2309469 | 182,222,075 | G | A | 0.01 | 0.05 | intergenic | LINC02500;TEMN3-AS1 | dist=35893;dist=519083 |  |
| 4 | rs2309468 | 182,222,133 | T | G | 0.01 | 0.05 | intergenic | LINC02500;TEMN3-AS1 | dist=35951;dist=519025 |  |
| 4 | rs2122062 | 189,806,414 | T | C | 0.02 | 0.05 | intergenic | LINC02508;LINC01262 | dist=99749;dist=774346 |  |
| 5 | rs1401623 | 23,025,189 | T | C | 9.78E-04 | 9.40E-03 | intergenic | CDH12;PRDM9 | dist=171736;dist=482529 |  |
| 5 | rs2680798 | 23,036,721 | T | C | 1.20E-03 | 0.02 | intergenic | CDH12;PRDM9 | dist=183268;dist=470997 |  |
| 5 | rs6884736 | 65,675,641 | G | A | 0.04 | 0.02 | intergenic | LINC02065;LINC02229 | dist=170367;dist=127732 |  |
| 5 | rs4246765 | 66,891,776 | T | C | 0.04 | 0.02 | intergenic | CD180;LINC02242 | dist=399149;dist=36318 |  |
| 5 | rs4277860 | 67,475,952 | G | A | 0.01 | 0.02 | intergenic | LOC101928858;LINC02219 | dist=379066;dist=9752 |  |
| 5 | rs7444646 | 101,507,854 | A | G | 0.02 | 9.48E-06 | intergenic | ST8SIA4;SLCO4C1 | dist=1268868;dist=61838 |  |
| 5 | rs3995228 | 101,513,985 | A | C | 0.02 | 1.03E-05 | intergenic | ST8SIA4;SLCO4C1 | dist=1274999;dist=55707 |  |
| 5 | rs6869235 | 101,519,912 | T | G | 0.04 | 6.88E-05 | intergenic | ST8SIA4;SLCO4C1 | dist=1280926;dist=49780 |  |
| 5 | rs12657697 | 101,559,063 | T | G | 0.02 | 2.80E-04 | intergenic | ST8SIA4;SLCO4C1 | dist=1320077;dist=10629 |  |
| 5 | rs10479269 | 103,093,213 | T | C | 0.04 | 2.07E-03 | intergenic | NUDT12;RAB9BP1 | dist=194723;dist=1341962 |  |
| 5 | rs6881174 | 103,093,272 | G | A | 0.04 | 1.94E-03 | intergenic | NUDT12;RAB9BP1 | dist=194782;dist=1341903 |  |
| 5 | rs9327891 | 103,093,295 | A | G | 0.04 | 2.14E-03 | intergenic | NUDT12;RAB9BP1 | dist=194805;dist=1341880 |  |
| 5 | rs9327892 | 103,093,580 | G | A | 0.04 | 2.11E-03 | intergenic | NUDT12;RAB9BP1 | dist=195090;dist=1341595 |  |
| 5 | rs1946256 | 103,093,763 | G | A | 0.04 | 2.03E-03 | intergenic | NUDT12;RAB9BP1 | dist=195273;dist=1341412 |  |
| 5 | rs1388106 | 118,909,149 | A | G | 0.01 | 2.65E-03 | intergenic | HSD17B4;FAM170A | dist=31122;dist=56104 |  |
| 5 | rs2407211 | 121,884,439 | G | A | 0.03 | 0.01 | intergenic | MGC32805;LOC101927357 | dist=69657;dist=32755 |  |
| 5 | rs2045902 | 121,887,289 | A | G | 0.04 | 0.01 | intergenic | MGC32805;LOC101927357 | dist=72507;dist=29905 |  |
| 5 | rs4836049 | 121,889,627 | A | G | 0.04 | 0.01 | intergenic | MGC32805;LOC101927357 | dist=74845;dist=27567 |  |
| 5 | rs4836050 | 121,889,791 | G | A | 0.03 | 0.01 | intergenic | MGC32805;LOC101927357 | dist=75009;dist=27403 |  |
| 5 | rs2407214 | 121,902,428 | A | G | 0.03 | 5.25E-03 | intergenic | MGC32805;LOC101927357 | dist=87646;dist=14766 |  |
| 5 | rs468579 | 143,947,058 | G | A | 8.69E-03 | 0.02 | intergenic | KCTD16;PRELID2 | dist=81809;dist=1188849 |  |
| 5 | rs165444 | 143,966,170 | G | A | 0.01 | 0.03 | intergenic | KCTD16;PRELID2 | dist=100921;dist=1169737 |  |
| 5 | rs165445 | 143,966,681 | A | G | 0.04 | 8.94E-03 | intergenic | KCTD16;PRELID2 | dist=101432;dist=1169226 |  |
| 5 | rs165487 | 143,976,648 | C | T | 6.32E-03 | 0.04 | intergenic | KCTD16;PRELID2 | dist=111399;dist=1159259 |  |
| 5 | rs7704625 | 144,064,654 | A | G | 0.01 | 0.03 | intergenic | KCTD16;PRELID2 | dist=199405;dist=1071253 |  |
| 5 | rs724382 | 144,065,162 | G | A | 0.01 | 0.03 | intergenic | KCTD16;PRELID2 | dist=199913;dist=1070745 |  |
| 5 | rs10039932 | 144,069,311 | T | C | 0.02 | 0.03 | intergenic | KCTD16;PRELID2 | dist=204062;dist=1066596 |  |
| 5 | rs917373 | 144,088,062 | T | C | 0.02 | 0.03 | intergenic | KCTD16;PRELID2 | dist=222813;dist=1047845 |  |
| 5 | rs13179675 | 144,088,792 | C | T | 0.02 | 0.03 | intergenic | KCTD16;PRELID2 | dist=223543;dist=1047115 |  |
| 5 | rs10477246 | 144,089,998 | C | T | 0.02 | 0.03 | intergenic | KCTD16;PRELID2 | dist=224749;dist=1045909 |  |
| 5 | rs1366220 | 153,497,780 | A | G | 0.02 | 0.04 | intergenic | MFAP3;GALNT10 | dist=60771;dist=72513 |  |
| 5 | rs815606 | 153,515,166 | G | A | 0.04 | 0.04 | intergenic | MFAP3;GALNT10 | dist=78157;dist=55127 |  |
| 5 | rs1863986 | 153,536,835 | T | C | 2.72E-03 | 0.01 | intergenic | MFAP3;GALNT10 | dist=99826;dist=33458 |  |
| 5 | rs7719067 | 153,538,241 | A | G | 2.87E-04 | 0.02 | intergenic | MFAP3;GALNT10 | dist=101232;dist=32052 |  |
| 5 | rs6580054 | 153,548,134 | T | C | 2.18E-03 | 0.03 | intergenic | MFAP3;GALNT10 | dist=111125;dist=22159 |  |
| 5 | rs13180419 | 160,495,163 | G | A | 0.04 | 0.04 | intergenic | LINC02159;GABRB2 | dist=129530;dist=220273 |  |
| 5 | rs10061064 | 164,177,109 | T | G | 0.03 | 0.03 | intergenic | LOC102546299;NONE | dist=207120;dist=NONE |  |
| 5 | rs830230 | 165,402,097 | C | T | 0.04 | 0.04 | intergenic | LOC102546299;LINC01947 | dist=1432108;dist=930130 |  |
| 6 | rs9394119 | 11,500,617 | G | T | 0.05 | 2.83E-05 | intergenic | NEDD9;TMEM170B | dist=118036;dist=37365 |  |
| 6 | rs620729 | 11,508,671 | C | A | 0.01 | 1.21E-04 | intergenic | NEDD9;TMEM170B | dist=126090;dist=29311 |  |
| 6 | rs513870 | 11,508,854 | T | C | 0.01 | 1.16E-04 | intergenic | NEDD9;TMEM170B | dist=126273;dist=29128 |  |
| 6 | rs538827 | 11,509,323 | T | C | 0.01 | 1.20E-04 | intergenic | NEDD9;TMEM170B | dist=126742;dist=28659 |  |
| 6 | rs490280 | 11,622,104 | A | G | 2.47E-03 | 0.02 | intergenic | TMEM170B;ADTRP | dist=38347;dist=91784 |  |
| 6 | rs807847 | 21,298,254 | A | C | 3.44E-03 | 0.03 | intergenic | CDKAL1;LINC00581 | dist=65620;dist=188038 |  |
| 6 | rs807848 | 21,298,295 | T | C | 7.90E-03 | 0.04 | intergenic | CDKAL1;LINC00581 | dist=65661;dist=187997 |  |
| 6 | rs9461403 | 27,713,528 | T | G | 0.01 | 0.03 | intergenic | LINC01012;LOC100131289 | dist=35527;dist=15995 |  |
| 6 | rs9468220 | 27,732,976 | T | C | 0.04 | 0.01 | intergenic | LOC100131289;H2BC13 | dist=2010;dist=42281 |  |
| 6 | rs742047 | 27,739,380 | G | A | 0.04 | 0.02 | intergenic | LOC100131289;H2BC13 | dist=8414;dist=35877 |  |
| 6 | rs9461412 | 27,771,654 | C | T | 0.02 | 0.03 | intergenic | LOC100131289;H2BC13 | dist=40688;dist=3603 |  |
| 6 | rs2853930 | 31,255,424 | C | A | 0.05 | 0.04 | intergenic | HLA-C;LINC02571 | dist=15511;dist=6261 |  |
| 6 | rs2524051 | 31,255,500 | G | A | 0.05 | 0.04 | intergenic | HLA-C;LINC02571 | dist=15587;dist=6185 |  |
| 6 | rs2524050 | 31,255,541 | C | T | 0.05 | 0.03 | intergenic | HLA-C;LINC02571 | dist=15628;dist=6144 |  |
| 6 | rs2524043 | 31,257,012 | G | A | 0.05 | 0.03 | intergenic | HLA-C;LINC02571 | dist=17099;dist=4673 |  |
| 6 | rs1819788 | 31,259,137 | C | T | 0.05 | 0.03 | intergenic | HLA-C;LINC02571 | dist=19224;dist=2548 |  |
| 6 | rs2524160 | 31,259,854 | A | G | 0.05 | 0.03 | intergenic | HLA-C;LINC02571 | dist=19941;dist=1831 |  |
| 6 | rs9264916 | 31,272,774 | A | G | 0.02 | 0.03 | intergenic | LINC02571;HLA-B | dist=3360;dist=48878 |  |
| 6 | rs9265668 | 31,300,796 | A | G | 0.02 | 0.01 | intergenic | LINC02571;HLA-B | dist=31382;dist=20856 |  |
| 6 | rs9394163 | 33,682,414 | C | T | 4.71E-04 | 2.60E-03 | intergenic | UQCC2;IP6K3 | dist=2937;dist=7025 |  |
| 6 | rs2894342 | 33,774,394 | A | C | 0.05 | 1.30E-03 | intergenic | MLN;LINC01016 | dist=2614;dist=82894 |  |
| 6 | rs1547669 | 33,775,641 | G | A | 7.79E-03 | 0.01 | intergenic | MLN;LINC01016 | dist=3861;dist=81647 |  |
| 6 | rs13202307 | 42,845,795 | A | G | 0.04 | 0.04 | intergenic | BICRAL;RPL7L1 | dist=9497;dist=1559 |  |
| 6 | rs1327275 | 51,278,035 | G | A | 4.55E-03 | 0.02 | intergenic | TFAP2B;PKHD1 | dist=462703;dist=202062 |  |
| 6 | rs2153883 | 51,278,193 | T | C | 5.40E-03 | 0.02 | intergenic | TFAP2B;PKHD1 | dist=462861;dist=201904 |  |
| 6 | rs3799936 | 62,342,243 | C | T | 0.04 | 0.01 | intergenic | MTRNR2L9;KHDRBS2 | dist=57708;dist=47622 |  |
| 6 | rs995863 | 71,815,318 | A | G | 0.05 | 7.93E-04 | intergenic | B3GAT2;OGFRL1 | dist=148555;dist=183196 |  |
| 6 | rs1418673 | 71,890,237 | T | G | 0.04 | 0.01 | intergenic | B3GAT2;OGFRL1 | dist=223474;dist=108277 |  |
| 6 | rs2502525 | 75,413,397 | C | T | 0.01 | 0.03 | intergenic | LOC101928516;COL12A1 | dist=12954;dist=380645 |  |
| 6 | rs2502530 | 75,415,378 | A | G | 0.04 | 0.04 | intergenic | LOC101928516;COL12A1 | dist=14935;dist=378664 |  |
| 6 | rs7739166 | 85,737,235 | T | C | 0.02 | 0.05 | intergenic | TBX18;LINC02535 | dist=262919;dist=359702 |  |
| 6 | rs6902772 | 99,550,464 | C | A | 0.02 | 0.05 | intergenic | FBXL4;MIR548AI | dist=154582;dist=22021 |  |
| 6 | rs6940049 | 99,551,169 | C | T | 0.02 | 5.38E-03 | intergenic | FBXL4;MIR548AI | dist=155287;dist=21316 |  |
| 6 | rs6569850 | 99,551,277 | A | C | 0.02 | 5.57E-03 | intergenic | FBXL4;MIR548AI | dist=155395;dist=21208 |  |
| 6 | rs6929928 | 99,552,248 | C | T | 0.02 | 0.05 | intergenic | FBXL4;MIR548AI | dist=156366;dist=20237 |  |
| 6 | rs1361417 | 102,738,413 | C | T | 5.95E-03 | 0.03 | intergenic | GRIK2;NONE | dist=220455;dist=NONE |  |
| 6 | rs954551 | 102,779,335 | G | A | 0.03 | 0.02 | intergenic | GRIK2;NONE | dist=261377;dist=NONE |  |
| 6 | rs9499927 | 105,153,614 | C | T | 0.02 | 5.20E-03 | intergenic | NONE;HACE1 | dist=NONE;dist=22355 |  |
| 6 | rs13204440 | 105,155,869 | T | C | 0.02 | 6.13E-03 | intergenic | NONE;HACE1 | dist=NONE;dist=20100 |  |
| 6 | rs9499934 | 105,164,613 | T | C | 0.03 | 5.20E-03 | intergenic | NONE;HACE1 | dist=NONE;dist=11356 |  |
| 6 | rs9499937 | 105,167,260 | T | C | 0.04 | 6.31E-03 | intergenic | NONE;HACE1 | dist=NONE;dist=8709 |  |
| 6 | rs6938458 | 138,033,789 | G | A | 6.80E-03 | 0.02 | intergenic | LOC102723649;LINC02539 | dist=38098;dist=17518 |  |
| 6 | rs9376923 | 145,708,978 | A | G | 0.03 | 0.03 | intergenic | UTRN;EPM2A | dist=534808;dist=237463 |  |
| 6 | rs12664698 | 145,709,371 | G | A | 0.03 | 0.02 | intergenic | UTRN;EPM2A | dist=535201;dist=237070 |  |
| 6 | rs9376924 | 145,715,513 | C | A | 5.70E-03 | 0.03 | intergenic | UTRN;EPM2A | dist=541343;dist=230928 |  |
| 6 | rs592374 | 147,735,166 | G | T | 0.03 | 8.51E-03 | intergenic | STXBP5;SAMD5 | dist=23554;dist=94660 |  |
| 6 | rs2675743 | 165,505,146 | G | A | 0.04 | 0.01 | intergenic | MEAT6;C6orf118 | dist=269594;dist=188007 |  |
| 7 | rs2396963 | 3,101,370 | C | T | 0.04 | 0.03 | intergenic | CARD11;LOC100129603 | dist=17869;dist=79195 |  |
| 7 | rs11983202 | 20,282,736 | T | C | 6.42E-03 | 0.01 | intergenic | LOC100506098;LOC101927769 | dist=21414;dist=53595 |  |
| 7 | rs17464305 | 21,398,126 | A | C | 0.01 | 0.02 | intergenic | LINC01162;SP4 | dist=336355;dist=69575 |  |
| 7 | rs10253780 | 21,399,377 | A | G | 0.01 | 0.02 | intergenic | LINC01162;SP4 | dist=337606;dist=68324 |  |
| 7 | rs2528907 | 22,095,150 | G | A | 3.81E-03 | 4.86E-05 | intergenic | CDCA7L;RAPGEF5 | dist=109633;dist=62704 |  |
| 7 | rs809297 | 46,068,074 | G | A | 0.03 | 0.04 | intergenic | IGFBP3;LOC730338 | dist=107203;dist=659403 |  |
| 7 | rs4718719 | 67,676,271 | T | C | 0.03 | 8.18E-03 | intergenic | LOC102723427;CT66 | dist=178594;dist=1383519 |  |
| 7 | rs4718720 | 67,676,780 | A | G | 0.04 | 7.37E-03 | intergenic | LOC102723427;CT66 | dist=179103;dist=1383010 |  |
| 7 | rs17164033 | 88,371,952 | T | C | 0.01 | 0.02 | intergenic | STEAP4;ZNF804B | dist=435743;dist=17062 |  |
| 7 | rs6944579 | 100,971,604 | G | A | 0.02 | 6.10E-03 | intergenic | IFT22;COL26A1 | dist=6500;dist=34497 |  |
| 7 | rs6946907 | 100,983,708 | T | G | 0.03 | 3.53E-03 | intergenic | IFT22;COL26A1 | dist=18604;dist=22393 |  |
| 7 | rs11534043 | 108,239,402 | A | G | 0.04 | 0.03 | intergenic | DNAJB9;C7orf66 | dist=24108;dist=284630 |  |
| 7 | rs11536567 | 108,239,477 | C | T | 0.03 | 0.03 | intergenic | DNAJB9;C7orf66 | dist=24183;dist=284555 |  |
| 7 | rs4728090 | 127,830,114 | T | C | 0.04 | 0.04 | intergenic | SND1;MIR129-1 | dist=97469;dist=17811 |  |
| 7 | rs368043 | 138,002,082 | A | G | 0.02 | 0.04 | intergenic | MIR4468;TRIM24 | dist=193515;dist=142922 |  |
| 7 | rs7790696 | 145,772,295 | G | A | 0.05 | 0.05 | intergenic | TPK1;CNTNAP2 | dist=1239149;dist=699068 |  |
| 8 | rs2816512 | 5,773,934 | T | C | 0.04 | 0.02 | intergenic | CSMD1;LOC100287015 | dist=921498;dist=487143 |  |
| 8 | rs1381350 | 9,217,195 | T | C | 8.44E-03 | 0.01 | intergenic | LOC157273;TNKS | dist=24605;dist=196227 |  |
| 8 | rs7837054 | 9,652,735 | A | G | 9.63E-03 | 0.01 | intergenic | TNKS;LINC00599 | dist=12879;dist=104839 |  |
| 8 | rs17411601 | 19,977,043 | T | G | 0.04 | 0.01 | intergenic | LPL;SLC18A1 | dist=152273;dist=25323 |  |
| 8 | rs4922205 | 20,306,252 | C | T | 0.02 | 0.01 | intergenic | LZTS1;SNORD3F | dist=144778;dist=166088 |  |
| 8 | rs10106340 | 20,337,794 | T | C | 0.02 | 0.04 | intergenic | LZTS1;SNORD3F | dist=176320;dist=134546 |  |
| 8 | rs17089579 | 23,600,918 | T | C | 0.03 | 0.02 | intergenic | NKX2-6;STC1 | dist=36807;dist=98524 |  |
| 8 | rs17089580 | 23,601,025 | T | G | 0.02 | 0.02 | intergenic | NKX2-6;STC1 | dist=36914;dist=98417 |  |
| 8 | rs12546407 | 34,322,406 | G | A | 0.05 | 0.05 | intergenic | DUSP26;LINC01288 | dist=864782;dist=319033 |  |
| 8 | rs7004498 | 34,524,109 | A | G | 0.04 | 0.02 | intergenic | DUSP26;LINC01288 | dist=1066485;dist=117330 |  |
| 8 | rs6987943 | 47,799,943 | T | G | 0.02 | 0.03 | intergenic | LINC00293;LOC100287846 | dist=32536;dist=300987 |  |
| 8 | rs11775587 | 52,820,165 | C | T | 0.03 | 0.05 | intergenic | PCMTD1;ST18 | dist=8419;dist=203227 |  |
| 8 | rs11775611 | 52,820,274 | C | T | 0.04 | 0.05 | intergenic | PCMTD1;ST18 | dist=8528;dist=203118 |  |
| 8 | rs17422653 | 55,878,305 | A | G | 0.03 | 0.02 | intergenic | RP1;XKR4 | dist=334911;dist=136283 |  |
| 8 | rs6998050 | 61,052,151 | A | G | 0.03 | 0.01 | intergenic | LOC100505501;CA8 | dist=1016114;dist=45820 |  |
| 8 | rs6998241 | 61,052,304 | A | G | 0.03 | 0.01 | intergenic | LOC100505501;CA8 | dist=1016267;dist=45667 |  |
| 8 | rs6984134 | 61,052,454 | C | T | 0.03 | 9.34E-03 | intergenic | LOC100505501;CA8 | dist=1016417;dist=45517 |  |
| 8 | rs7011237 | 61,243,357 | A | G | 0.03 | 0.01 | intergenic | CA8;LINC01301 | dist=49398;dist=71373 |  |
| 8 | rs13260204 | 64,207,363 | A | G | 7.59E-04 | 0.04 | intergenic | YTHDF3;LOC102724612 | dist=82017;dist=171044 |  |
| 8 | rs1367566 | 77,102,891 | T | C | 0.05 | 2.09E-03 | intergenic | HNF4G;LINC01111 | dist=623830;dist=215998 |  |
| 8 | rs13251222 | 79,045,302 | G | A | 0.03 | 0.05 | intergenic | LOC102724874;PKIA | dist=656735;dist=383273 |  |
| 8 | rs202277 | 82,201,519 | A | G | 0.02 | 0.01 | intergenic | FABP5;PMP2 | dist=4509;dist=151044 |  |
| 8 | rs202279 | 82,202,118 | T | C | 0.03 | 0.01 | intergenic | FABP5;PMP2 | dist=5108;dist=150445 |  |
| 8 | rs17667972 | 83,452,981 | G | T | 0.04 | 0.04 | intergenic | SNX16;LOC101927141 | dist=698460;dist=371358 |  |
| 8 | rs13263353 | 87,244,582 | A | G | 8.45E-03 | 8.57E-03 | intergenic | SLC7A13;WWP1 | dist=1972;dist=110194 |  |
| 8 | rs4310184 | 87,245,253 | C | T | 6.01E-03 | 5.95E-03 | intergenic | SLC7A13;WWP1 | dist=2643;dist=109523 |  |
| 8 | rs10103229 | 87,248,954 | C | T | 0.04 | 7.16E-03 | intergenic | SLC7A13;WWP1 | dist=6344;dist=105822 |  |
| 8 | rs10085920 | 87,252,666 | T | C | 7.81E-03 | 6.80E-03 | intergenic | SLC7A13;WWP1 | dist=10056;dist=102110 |  |
| 8 | rs9283958 | 87,253,048 | C | T | 0.02 | 7.42E-03 | intergenic | SLC7A13;WWP1 | dist=10438;dist=101728 |  |
| 8 | rs10435563 | 87,266,060 | T | C | 6.16E-03 | 0.01 | intergenic | SLC7A13;WWP1 | dist=23450;dist=88716 |  |
| 8 | rs17743495 | 117,652,242 | T | C | 4.28E-03 | 0.02 | intergenic | LINC00536;EIF3H | dist=314945;dist=2128 |  |
| 8 | rs2954025 | 126,484,463 | C | T | 0.04 | 5.55E-03 | intergenic | TRIB1;LINC00861 | dist=33816;dist=450304 |  |
| 8 | rs12114450 | 128,563,712 | T | C | 0.04 | 0.03 | intergenic | CASC8;CASC11 | dist=69328;dist=149141 |  |
| 8 | rs10956671 | 133,564,247 | A | C | 0.05 | 0.03 | intergenic | KCNQ3;HPYR1 | dist=70905;dist=8498 |  |
| 9 | rs10116586 | 14,058,548 | C | A | 2.68E-03 | 3.89E-03 | intergenic | LINC00583;NFIB | dist=112942;dist=23294 |  |
| 9 | rs1452653 | 21,573,016 | T | C | 0.01 | 0.02 | intergenic | MIR31HG;MTAP | dist=13219;dist=229619 |  |
| 9 | rs1934113 | 23,079,926 | A | G | 0.02 | 0.04 | intergenic | LINC01239;LOC101929563 | dist=255714;dist=420763 |  |
| 9 | rs2026589 | 24,841,259 | C | T | 0.02 | 5.59E-03 | intergenic | IZUMO3;TUSC1 | dist=295364;dist=835128 |  |
| 9 | rs1125204 | 30,525,534 | G | A | 0.04 | 0.05 | intergenic | LINC01242;LINC01243 | dist=117082;dist=846075 |  |
| 9 | rs1857553 | 30,546,359 | T | C | 0.04 | 0.04 | intergenic | LINC01242;LINC01243 | dist=137907;dist=825250 |  |
| 9 | rs10969739 | 30,551,595 | A | G | 0.04 | 0.05 | intergenic | LINC01242;LINC01243 | dist=143143;dist=820014 |  |
| 9 | rs4878434 | 30,551,669 | C | T | 0.04 | 0.04 | intergenic | LINC01242;LINC01243 | dist=143217;dist=819940 |  |
| 9 | rs17178103 | 30,552,008 | G | A | 0.04 | 0.04 | intergenic | LINC01242;LINC01243 | dist=143556;dist=819601 |  |
| 9 | rs2361134 | 30,553,329 | G | T | 0.04 | 0.04 | intergenic | LINC01242;LINC01243 | dist=144877;dist=818280 |  |
| 9 | rs2361135 | 30,553,483 | T | C | 0.04 | 0.04 | intergenic | LINC01242;LINC01243 | dist=145031;dist=818126 |  |
| 9 | rs1556512 | 30,555,044 | C | T | 0.02 | 0.04 | intergenic | LINC01242;LINC01243 | dist=146592;dist=816565 |  |
| 9 | rs4084038 | 30,558,753 | A | G | 0.04 | 0.05 | intergenic | LINC01242;LINC01243 | dist=150301;dist=812856 |  |
| 9 | rs12553991 | 30,564,985 | A | C | 0.02 | 0.04 | intergenic | LINC01242;LINC01243 | dist=156533;dist=806624 |  |
| 9 | rs1361917 | 30,580,708 | G | A | 0.04 | 0.03 | intergenic | LINC01242;LINC01243 | dist=172256;dist=790901 |  |
| 9 | rs1104788 | 30,591,801 | A | G | 0.02 | 0.03 | intergenic | LINC01242;LINC01243 | dist=183349;dist=779808 |  |
| 9 | rs10969777 | 30,604,262 | G | A | 0.04 | 0.03 | intergenic | LINC01242;LINC01243 | dist=195810;dist=767347 |  |
| 9 | rs10813616 | 31,550,664 | T | C | 0.02 | 0.02 | intergenic | LINC01243;ACO1 | dist=169176;dist=833937 |  |
| 9 | rs7034481 | 31,553,846 | A | G | 0.02 | 0.03 | intergenic | LINC01243;ACO1 | dist=172358;dist=830755 |  |
| 9 | rs7030774 | 79,611,749 | T | C | 0.03 | 0.04 | intergenic | PRUNE2;FOXB2 | dist=90719;dist=22822 |  |
| 9 | rs7864598 | 79,643,960 | C | T | 5.83E-03 | 5.42E-03 | intergenic | FOXB2;VPS13A-AS1 | dist=8091;dist=147712 |  |
| 9 | rs2788106 | 84,955,250 | G | T | 0.05 | 1.28E-03 | intergenic | SPATA31D1;RASEF | dist=345082;dist=639255 |  |
| 9 | rs16905942 | 92,716,596 | A | G | 0.05 | 0.02 | intergenic | UNQ6494;LOC101927847 | dist=381922;dist=15102 |  |
| 9 | rs280661 | 98,991,519 | G | A | 9.56E-03 | 0.05 | intergenic | LOC158434;HSD17B3 | dist=112826;dist=6069 |  |
| 9 | rs692905 | 102,049,363 | C | T | 0.03 | 4.10E-03 | intergenic | SEC61B;NAMA | dist=56466;dist=68259 |  |
| 9 | rs1648628 | 102,050,025 | T | C | 0.03 | 3.11E-03 | intergenic | SEC61B;NAMA | dist=57128;dist=67597 |  |
| 9 | rs1360260 | 102,222,771 | C | A | 0.04 | 7.95E-03 | intergenic | NAMA;LOC101928438 | dist=85232;dist=125297 |  |
| 9 | rs10760688 | 102,247,368 | G | A | 0.05 | 5.25E-03 | intergenic | NAMA;LOC101928438 | dist=109829;dist=100700 |  |
| 9 | rs1888162 | 102,250,037 | T | C | 0.05 | 5.54E-03 | intergenic | NAMA;LOC101928438 | dist=112498;dist=98031 |  |
| 9 | rs10978329 | 108,642,814 | A | G | 0.03 | 0.02 | intergenic | TMEM38B;MIR8081 | dist=103904;dist=720395 |  |
| 9 | rs4742971 | 108,655,096 | T | G | 9.69E-03 | 0.02 | intergenic | TMEM38B;MIR8081 | dist=116186;dist=708113 |  |
| 9 | rs7866652 | 108,732,469 | C | T | 0.02 | 0.04 | intergenic | TMEM38B;MIR8081 | dist=193559;dist=630740 |  |
| 9 | rs10978534 | 109,317,378 | T | G | 0.01 | 0.01 | intergenic | TMEM38B;MIR8081 | dist=778468;dist=45831 |  |
| 9 | rs10978638 | 109,545,918 | G | T | 0.04 | 5.60E-03 | intergenic | LINC01505;ZNF462 | dist=103837;dist=79529 |  |
| 9 | rs10759402 | 113,000,213 | T | C | 0.03 | 0.03 | intergenic | C9orf152;TXN | dist=29800;dist=5877 |  |
| 9 | rs10984647 | 122,457,146 | T | C | 1.38E-03 | 0.02 | intergenic | BRINP1;LINC01613 | dist=325433;dist=240192 |  |
| 9 | rs10984655 | 122,474,092 | T | C | 0.02 | 4.48E-03 | intergenic | BRINP1;LINC01613 | dist=342379;dist=223246 |  |
| 9 | rs17386987 | 122,484,432 | A | G | 0.03 | 2.82E-03 | intergenic | BRINP1;LINC01613 | dist=352719;dist=212906 |  |
| 10 | rs7092925 | 13,306,173 | C | T | 0.02 | 0.03 | intergenic | UCMA;PHYH | dist=29799;dist=13623 |  |
| 10 | rs11592119 | 16,021,427 | T | G | 0.01 | 1.83E-03 | intergenic | MINDY3;PTER | dist=118895;dist=457515 |  |
| 10 | rs4747256 | 16,045,536 | T | C | 0.04 | 0.02 | intergenic | MINDY3;PTER | dist=143004;dist=433406 |  |
| 10 | rs3936639 | 57,458,863 | G | A | 4.46E-03 | 8.85E-05 | intergenic | PCDH15;ZWINT | dist=71161;dist=658126 |  |
| 10 | rs12413022 | 58,729,512 | A | G | 0.05 | 0.01 | intergenic | ZWINT;MIR3924 | dist=608478;dist=334727 |  |
| 10 | rs7068823 | 58,736,121 | A | G | 0.04 | 9.93E-03 | intergenic | ZWINT;MIR3924 | dist=615087;dist=328118 |  |
| 10 | rs4746324 | 65,450,010 | T | G | 0.04 | 0.02 | intergenic | REEP3;ANXA2P3 | dist=65122;dist=1135275 |  |
| 10 | rs1255476 | 65,452,546 | A | G | 0.02 | 0.03 | intergenic | REEP3;ANXA2P3 | dist=67658;dist=1132739 |  |
| 10 | rs17490500 | 71,206,830 | C | A | 0.03 | 0.02 | intergenic | TACR2;TSPAN15 | dist=30156;dist=4391 |  |
| 10 | rs12414198 | 71,209,768 | C | T | 0.02 | 0.02 | intergenic | TACR2;TSPAN15 | dist=33094;dist=1453 |  |
| 10 | rs947366 | 72,908,675 | T | C | 0.02 | 1.94E-03 | intergenic | LINC02622;UNC5B | dist=193277;dist=63652 |  |
| 10 | rs2688609 | 75,658,181 | A | G | 0.04 | 0.02 | intergenic | CAMK2G;C10orf55 | dist=23832;dist=11546 |  |
| 10 | rs2688608 | 75,658,349 | T | G | 0.04 | 0.03 | intergenic | CAMK2G;C10orf55 | dist=24000;dist=11378 |  |
| 10 | rs2675679 | 75,658,581 | A | G | 0.04 | 0.02 | intergenic | CAMK2G;C10orf55 | dist=24232;dist=11146 |  |
| 10 | rs11591951 | 80,551,916 | T | C | 0.04 | 0.04 | intergenic | LINC00595;ZMIZ1-AS1 | dist=511946;dist=151167 |  |
| 10 | rs11002673 | 80,552,153 | T | C | 0.04 | 0.04 | intergenic | LINC00595;ZMIZ1-AS1 | dist=512183;dist=150930 |  |
| 10 | rs11002674 | 80,552,247 | G | A | 0.04 | 0.05 | intergenic | LINC00595;ZMIZ1-AS1 | dist=512277;dist=150836 |  |
| 10 | rs609749 | 85,461,469 | A | G | 0.02 | 0.03 | intergenic | LINC02650;GHITM | dist=24624;dist=437796 |  |
| 10 | rs608803 | 85,461,728 | A | G | 0.02 | 0.02 | intergenic | LINC02650;GHITM | dist=24883;dist=437537 |  |
| 10 | rs10882654 | 97,468,589 | T | G | 0.04 | 2.08E-03 | intergenic | TCTN3;ENTPD1 | dist=14689;dist=2932 |  |
| 10 | rs1415557 | 110,504,802 | A | G | 0.02 | 3.06E-10 | intergenic | LINC01435;XPNPEP1 | dist=675751;dist=1119722 |  |
| 10 | rs7922390 | 110,505,926 | A | G | 0.01 | 5.43E-10 | intergenic | LINC01435;XPNPEP1 | dist=676875;dist=1118598 |  |
| 10 | rs7085179 | 110,524,102 | C | T | 0.02 | 7.61E-10 | intergenic | LINC01435;XPNPEP1 | dist=695051;dist=1100422 |  |
| 10 | rs4369324 | 110,530,574 | T | G | 4.90E-03 | 9.90E-10 | intergenic | LINC01435;XPNPEP1 | dist=701523;dist=1093950 |  |
| 10 | rs945824 | 110,541,074 | T | C | 0.03 | 7.90E-08 | intergenic | LINC01435;XPNPEP1 | dist=712023;dist=1083450 |  |
| 10 | rs7920198 | 110,547,803 | G | A | 0.02 | 8.70E-08 | intergenic | LINC01435;XPNPEP1 | dist=718752;dist=1076721 |  |
| 10 | rs1949432 | 125,683,978 | T | C | 5.81E-03 | 7.55E-04 | intergenic | CPXM2;CHST15 | dist=32655;dist=83206 |  |
| 10 | rs9794150 | 125,684,849 | C | T | 7.99E-03 | 1.26E-03 | intergenic | CPXM2;CHST15 | dist=33526;dist=82335 |  |
| 11 | rs10839916 | 7,930,640 | C | A | 0.05 | 0.01 | intergenic | LOC283299;OR10A6 | dist=3138;dist=18625 |  |
| 11 | rs1493657 | 25,761,824 | C | T | 0.02 | 0.02 | intergenic | LUZP2;ANO3 | dist=657640;dist=448846 |  |
| 11 | rs10767443 | 25,763,508 | G | A | 0.02 | 0.02 | intergenic | LUZP2;ANO3 | dist=659324;dist=447162 |  |
| 11 | rs12294925 | 25,763,919 | C | T | 0.03 | 0.03 | intergenic | LUZP2;ANO3 | dist=659735;dist=446751 |  |
| 11 | rs11028895 | 25,764,292 | T | G | 0.02 | 0.02 | intergenic | LUZP2;ANO3 | dist=660108;dist=446378 |  |
| 11 | rs7126987 | 25,771,209 | C | A | 0.01 | 0.02 | intergenic | LUZP2;ANO3 | dist=667025;dist=439461 |  |
| 11 | rs7112727 | 25,771,252 | T | G | 9.58E-03 | 0.02 | intergenic | LUZP2;ANO3 | dist=667068;dist=439418 |  |
| 11 | rs11028909 | 25,772,445 | G | A | 0.01 | 0.02 | intergenic | LUZP2;ANO3 | dist=668261;dist=438225 |  |
| 11 | rs2349308 | 25,773,322 | G | A | 0.01 | 0.03 | intergenic | LUZP2;ANO3 | dist=669138;dist=437348 |  |
| 11 | rs16914560 | 25,773,574 | T | G | 0.01 | 0.03 | intergenic | LUZP2;ANO3 | dist=669390;dist=437096 |  |
| 11 | rs12281442 | 25,777,091 | T | C | 0.01 | 0.03 | intergenic | LUZP2;ANO3 | dist=672907;dist=433579 |  |
| 11 | rs1493663 | 25,779,039 | A | G | 0.01 | 0.02 | intergenic | LUZP2;ANO3 | dist=674855;dist=431631 |  |
| 11 | rs1079303 | 27,269,598 | C | T | 0.05 | 0.02 | intergenic | BBOX1-AS1;CCDC34 | dist=27938;dist=90461 |  |
| 11 | rs11030026 | 27,510,830 | A | G | 3.46E-03 | 3.56E-03 | intergenic | LGR4-AS1;LIN7C | dist=6850;dist=5135 |  |
| 11 | rs1491856 | 27,845,989 | T | C | 0.04 | 0.02 | intergenic | BDNF;KIF18A | dist=102412;dist=196177 |  |
| 11 | rs2726825 | 27,878,366 | C | T | 0.03 | 0.01 | intergenic | BDNF;KIF18A | dist=134789;dist=163800 |  |
| 11 | rs11030161 | 27,932,781 | A | G | 0.04 | 2.60E-03 | intergenic | BDNF;KIF18A | dist=189204;dist=109385 |  |
| 11 | rs162014 | 29,711,091 | T | C | 0.05 | 0.01 | intergenic | LINC02546;LINC01616 | dist=58870;dist=290577 |  |
| 11 | rs12271535 | 36,749,085 | A | G | 0.04 | 7.19E-03 | intergenic | C11orf74;LINC02760 | dist=68244;dist=1211066 |  |
| 11 | rs4756338 | 36,750,146 | T | C | 0.05 | 7.69E-03 | intergenic | C11orf74;LINC02760 | dist=69305;dist=1210005 |  |
| 11 | rs4756342 | 36,750,701 | A | G | 0.05 | 8.14E-03 | intergenic | C11orf74;LINC02760 | dist=69860;dist=1209450 |  |
| 11 | rs10838586 | 46,270,441 | G | T | 0.05 | 7.76E-03 | intergenic | LOC101928894;CREB3L1 | dist=71785;dist=28772 |  |
| 11 | rs10838587 | 46,272,585 | T | C | 0.01 | 5.15E-03 | intergenic | LOC101928894;CREB3L1 | dist=73929;dist=26628 |  |
| 11 | rs7945270 | 47,891,334 | A | G | 0.02 | 0.04 | intergenic | NUP160;PTPRJ | dist=21238;dist=110777 |  |
| 11 | rs1483121 | 48,333,360 | A | G | 0.03 | 1.06E-04 | intergenic | OR4S1;OR4C3 | dist=4656;dist=13112 |  |
| 11 | rs2360872 | 64,353,405 | C | T | 0.01 | 3.57E-03 | intergenic | SLC22A11;SLC22A12 | dist=13058;dist=4877 |  |
| 11 | rs12801811 | 81,359,141 | A | C | 9.61E-03 | 3.25E-04 | intergenic | LINC02720;MIR4300HG | dist=885295;dist=231752 |  |
| 11 | rs17817254 | 85,564,826 | C | A | 0.03 | 0.03 | intergenic | SYTL2;CCDC83 | dist=42624;dist=1318 |  |
| 11 | rs17833579 | 87,526,150 | C | T | 0.03 | 5.12E-03 | intergenic | LINC02711;RAB38 | dist=95847;dist=320269 |  |
| 11 | rs10501651 | 87,527,561 | A | G | 0.04 | 4.65E-03 | intergenic | LINC02711;RAB38 | dist=97258;dist=318858 |  |
| 11 | rs7114816 | 87,682,771 | C | T | 0.04 | 6.16E-03 | intergenic | LINC02711;RAB38 | dist=252468;dist=163648 |  |
| 11 | rs308757 | 88,158,865 | A | G | 0.01 | 8.77E-03 | intergenic | CTSC;GRM5 | dist=87961;dist=78879 |  |
| 11 | rs17130267 | 97,689,515 | C | A | 0.01 | 0.04 | intergenic | LINC02737;CNTN5 | dist=1441601;dist=1202164 | Y |
| 11 | rs10890666 | 107,114,199 | A | G | 0.03 | 0.03 | intergenic | GUCY1A2;CWF19L2 | dist=224997;dist=82872 |  |
| 11 | rs2216998 | 107,117,691 | C | T | 0.03 | 0.04 | intergenic | GUCY1A2;CWF19L2 | dist=228489;dist=79380 |  |
| 11 | rs7106065 | 107,122,143 | T | C | 0.04 | 0.03 | intergenic | GUCY1A2;CWF19L2 | dist=232941;dist=74928 |  |
| 11 | rs655917 | 107,439,257 | A | G | 0.02 | 2.06E-03 | intergenic | ALKBH8;ELMOD1 | dist=2796;dist=22560 |  |
| 11 | rs7127817 | 112,626,686 | T | C | 0.04 | 0.04 | intergenic | LINC02764;LOC101928847 | dist=200161;dist=203317 |  |
| 11 | rs10891549 | 113,278,447 | C | T | 0.02 | 2.32E-06 | intergenic | ANKK1;DRD2 | dist=7309;dist=1870 |  |
| 11 | rs1554929 | 113,278,764 | T | C | 0.01 | 2.90E-06 | intergenic | ANKK1;DRD2 | dist=7626;dist=1553 |  |
| 11 | rs10891564 | 113,374,013 | A | G | 1.47E-03 | 1.26E-07 | intergenic | DRD2;TMPRSS5 | dist=27893;dist=184255 |  |
| 11 | rs12222458 | 113,417,603 | T | C | 0.03 | 1.45E-04 | intergenic | DRD2;TMPRSS5 | dist=71483;dist=140665 |  |
| 11 | rs7125588 | 113,436,072 | G | A | 0.03 | 1.05E-13 | intergenic | DRD2;TMPRSS5 | dist=89952;dist=122196 |  |
| 11 | rs7937934 | 121,635,211 | T | C | 5.29E-03 | 7.49E-06 | intergenic | SORL1;MIR100HG | dist=130739;dist=263826 |  |
| 11 | rs1944683 | 121,641,496 | T | C | 8.55E-03 | 5.29E-04 | intergenic | SORL1;MIR100HG | dist=137024;dist=257541 |  |
| 11 | rs17120 | 123,694,039 | G | A | 0.03 | 0.02 | intergenic | OR6M1;TMEM225 | dist=16982;dist=59588 |  |
| 11 | rs2186745 | 123,717,398 | G | A | 0.02 | 0.02 | intergenic | OR6M1;TMEM225 | dist=40341;dist=36229 |  |
| 11 | rs948159 | 123,733,108 | G | A | 0.02 | 0.02 | intergenic | OR6M1;TMEM225 | dist=56051;dist=20519 |  |
| 11 | rs4936856 | 123,742,298 | C | A | 0.01 | 5.98E-03 | intergenic | OR6M1;TMEM225 | dist=65241;dist=11329 |  |
| 11 | rs11219660 | 124,313,035 | C | A | 0.02 | 0.01 | intergenic | OR8B8;OR8B12 | dist=2054;dist=99583 |  |
| 11 | rs1078278 | 124,318,707 | G | A | 0.02 | 0.01 | intergenic | OR8B8;OR8B12 | dist=7726;dist=93911 |  |
| 11 | rs9633957 | 124,326,024 | A | C | 0.02 | 0.01 | intergenic | OR8B8;OR8B12 | dist=15043;dist=86594 |  |
| 11 | rs11606364 | 130,506,001 | T | C | 0.05 | 0.04 | intergenic | ADAMTS15;MIR8052 | dist=159461;dist=30629 |  |
| 11 | rs1038480 | 133,848,212 | A | G | 0.04 | 0.02 | intergenic | IGSF9B;LINC02731 | dist=21521;dist=53955 |  |
| 11 | rs3017965 | 134,506,955 | C | A | 0.02 | 0.03 | intergenic | LOC283177;LINC02714 | dist=131400;dist=98884 |  |
| 12 | rs4640003 | 4,342,710 | G | A | 0.05 | 0.05 | intergenic | PARP11;CCND2-AS1 | dist=360096;dist=15223 |  |
| 12 | rs7309094 | 14,362,570 | C | T | 0.04 | 0.02 | intergenic | GRIN2B;ATF7IP | dist=229280;dist=155996 |  |
| 12 | rs12578518 | 14,408,741 | A | G | 2.43E-04 | 4.06E-03 | intergenic | GRIN2B;ATF7IP | dist=275451;dist=109825 |  |
| 12 | rs10772769 | 14,465,589 | A | G | 0.02 | 6.00E-03 | intergenic | GRIN2B;ATF7IP | dist=332299;dist=52977 |  |
| 12 | rs12310044 | 16,461,783 | G | A | 0.04 | 2.76E-03 | intergenic | SLC15A5;MGST1 | dist=31164;dist=38293 |  |
| 12 | rs10770214 | 17,462,225 | G | A | 0.04 | 0.03 | intergenic | SKP1P2;LINC02378 | dist=318663;dist=272532 |  |
| 12 | rs7970149 | 29,273,302 | C | A | 0.03 | 0.05 | intergenic | CCDC91;FAR2 | dist=570203;dist=28909 |  |
| 12 | rs4081964 | 29,273,524 | A | G | 0.03 | 0.05 | intergenic | CCDC91;FAR2 | dist=570425;dist=28687 |  |
| 12 | rs7310157 | 29,274,701 | T | C | 0.04 | 0.04 | intergenic | CCDC91;FAR2 | dist=571602;dist=27510 |  |
| 12 | rs10771646 | 30,304,193 | C | A | 0.02 | 0.04 | intergenic | TMTC1;IPO8 | dist=366501;dist=477722 |  |
| 12 | rs33162 | 30,989,067 | A | G | 0.05 | 0.03 | intergenic | LINC00941;TSPAN11 | dist=33422;dist=90615 |  |
| 12 | rs2127955 | 37,969,194 | T | C | 0.02 | 0.04 | intergenic | NONE;ALG10B | dist=NONE;dist=741382 | Y |
| 12 | rs7975604 | 47,290,149 | T | C | 0.01 | 1.14E-03 | intergenic | SLC38A4;AMIGO2 | dist=70369;dist=179341 |  |
| 12 | rs1106725 | 51,942,331 | G | A | 0.04 | 0.05 | intergenic | SLC4A8;SCN8A | dist=32784;dist=42686 |  |
| 12 | rs10506321 | 54,224,324 | A | G | 5.06E-03 | 9.62E-03 | intergenic | CISTR;HOXC13-AS | dist=73506;dist=104788 |  |
| 12 | rs12822602 | 54,224,680 | T | C | 5.32E-03 | 9.57E-03 | intergenic | CISTR;HOXC13-AS | dist=73862;dist=104432 |  |
| 12 | rs11170706 | 54,226,257 | C | T | 2.95E-03 | 9.15E-03 | intergenic | CISTR;HOXC13-AS | dist=75439;dist=102855 |  |
| 12 | rs7954829 | 54,227,826 | T | C | 5.30E-04 | 7.45E-03 | intergenic | CISTR;HOXC13-AS | dist=77008;dist=101286 |  |
| 12 | rs7956714 | 62,813,054 | G | A | 0.01 | 0.04 | intergenic | USP15;MON2 | dist=2886;dist=47552 |  |
| 12 | rs10784294 | 62,822,067 | A | G | 0.02 | 0.05 | intergenic | USP15;MON2 | dist=11899;dist=38539 |  |
| 12 | rs710695 | 70,373,848 | G | A | 7.36E-03 | 0.03 | intergenic | MYRFL;CNOT2-DT | dist=20971;dist=242122 |  |
| 12 | rs2102754 | 73,645,425 | G | A | 0.05 | 0.05 | intergenic | LINC02444;LOC100507377 | dist=43328;dist=881531 |  |
| 12 | rs12318428 | 89,775,186 | A | G | 6.81E-03 | 0.02 | intergenic | DUSP6;POC1B | dist=28908;dist=38312 |  |
| 12 | rs2859670 | 114,879,632 | T | C | 0.01 | 0.01 | intergenic | TBX5-AS1;TBX3 | dist=28995;dist=228428 |  |
| 13 | rs17078041 | 23,585,183 | A | G | 5.13E-03 | 0.02 | intergenic | LINC00621;SGCG | dist=94675;dist=169877 |  |
| 13 | rs9550895 | 23,587,273 | C | T | 9.37E-03 | 0.01 | intergenic | LINC00621;SGCG | dist=96765;dist=167787 |  |
| 13 | rs9579135 | 28,533,738 | G | A | 0.03 | 0.01 | intergenic | LINC00543;CDX2 | dist=4238;dist=1317 |  |
| 13 | rs9596054 | 31,607,186 | G | A | 4.25E-03 | 0.03 | intergenic | TEX26;HSPH1 | dist=57549;dist=101924 |  |
| 13 | rs17079663 | 34,307,900 | C | T | 0.03 | 9.96E-03 | intergenic | STARD13;RFC3 | dist=56969;dist=84306 |  |
| 13 | rs1886534 | 34,310,341 | C | T | 5.29E-03 | 7.17E-03 | intergenic | STARD13;RFC3 | dist=59410;dist=81865 |  |
| 13 | rs9598047 | 34,315,059 | T | C | 6.00E-03 | 0.02 | intergenic | STARD13;RFC3 | dist=64128;dist=77147 |  |
| 13 | rs1605644 | 35,300,861 | A | G | 0.05 | 1.40E-03 | intergenic | LINC00457;NBEA | dist=86039;dist=215563 |  |
| 13 | rs12875137 | 35,301,013 | C | T | 0.05 | 1.47E-03 | intergenic | LINC00457;NBEA | dist=86191;dist=215411 |  |
| 13 | rs7334366 | 56,775,051 | T | C | 0.03 | 0.03 | intergenic | MIR5007;PRR20B | dist=1026368;dist=940001 | Y |
| 13 | rs7319161 | 56,777,987 | T | C | 0.03 | 0.03 | intergenic | MIR5007;PRR20B | dist=1029304;dist=937065 | Y |
| 13 | rs338132 | 71,255,053 | T | C | 0.04 | 6.25E-04 | intergenic | ATXN8OS;LINC00348 | dist=541168;dist=334220 |  |
| 13 | rs337940 | 71,257,994 | T | C | 0.03 | 5.62E-04 | intergenic | ATXN8OS;LINC00348 | dist=544109;dist=331279 |  |
| 13 | rs7996101 | 81,086,353 | T | G | 0.05 | 0.03 | intergenic | SPRY2;LINC00377 | dist=171092;dist=506173 |  |
| 13 | rs11069910 | 87,185,216 | G | A | 0.03 | 0.01 | intergenic | SLITRK6;LINC00430 | dist=811662;dist=378959 |  |
| 13 | rs11069911 | 87,185,336 | T | C | 0.03 | 0.01 | intergenic | SLITRK6;LINC00430 | dist=811782;dist=378839 |  |
| 13 | rs11839615 | 87,185,823 | G | A | 0.03 | 0.01 | intergenic | SLITRK6;LINC00430 | dist=812269;dist=378352 |  |
| 13 | rs9301533 | 87,186,028 | T | C | 0.03 | 0.01 | intergenic | SLITRK6;LINC00430 | dist=812474;dist=378147 |  |
| 13 | rs9301535 | 87,186,282 | A | G | 0.03 | 0.01 | intergenic | SLITRK6;LINC00430 | dist=812728;dist=377893 |  |
| 13 | rs1410761 | 87,195,094 | A | C | 0.01 | 0.01 | intergenic | SLITRK6;LINC00430 | dist=821540;dist=369081 |  |
| 13 | rs7989577 | 87,197,723 | A | C | 0.04 | 0.01 | intergenic | SLITRK6;LINC00430 | dist=824169;dist=366452 |  |
| 13 | rs7987256 | 87,204,838 | C | A | 0.01 | 9.87E-03 | intergenic | SLITRK6;LINC00430 | dist=831284;dist=359337 |  |
| 13 | rs9560257 | 87,207,716 | C | T | 0.01 | 9.52E-03 | intergenic | SLITRK6;LINC00430 | dist=834162;dist=356459 |  |
| 13 | rs9555862 | 87,208,016 | C | A | 0.04 | 1.10E-03 | intergenic | SLITRK6;LINC00430 | dist=834462;dist=356159 |  |
| 13 | rs9560258 | 87,208,133 | T | C | 0.04 | 0.01 | intergenic | SLITRK6;LINC00430 | dist=834579;dist=356042 |  |
| 13 | rs2341301 | 87,215,321 | C | T | 0.03 | 0.01 | intergenic | SLITRK6;LINC00430 | dist=841767;dist=348854 |  |
| 13 | rs2996405 | 87,366,852 | C | T | 0.05 | 2.96E-04 | intergenic | SLITRK6;LINC00430 | dist=993298;dist=197323 |  |
| 13 | rs6492544 | 87,430,250 | C | T | 0.03 | 8.33E-05 | intergenic | SLITRK6;LINC00430 | dist=1056696;dist=133925 |  |
| 13 | rs9556162 | 87,507,269 | C | T | 0.04 | 4.51E-05 | intergenic | SLITRK6;LINC00430 | dist=1133715;dist=56906 |  |
| 13 | rs9556443 | 87,778,381 | C | T | 0.03 | 1.11E-03 | intergenic | LINC00430;MIR4500HG | dist=189319;dist=317861 |  |
| 13 | rs9634641 | 87,784,443 | C | T | 0.03 | 9.35E-04 | intergenic | LINC00430;MIR4500HG | dist=195381;dist=311799 |  |
| 13 | rs1967454 | 87,836,852 | G | A | 0.03 | 1.57E-03 | intergenic | LINC00430;MIR4500HG | dist=247790;dist=259390 |  |
| 13 | rs338754 | 88,980,273 | C | T | 0.03 | 0.01 | intergenic | LINC00373;LINC00433 | dist=91936;dist=212811 |  |
| 13 | rs394028 | 89,057,133 | C | A | 3.19E-03 | 1.78E-04 | intergenic | LINC00373;LINC00433 | dist=168796;dist=135951 |  |
| 13 | rs7983923 | 90,397,486 | G | T | 0.05 | 1.33E-03 | intergenic | LINC00353;LINC00559 | dist=180819;dist=315015 |  |
| 13 | rs9556267 | 93,839,833 | G | A | 0.05 | 2.10E-04 | intergenic | LINC00363;GPC6 | dist=129654;dist=39227 |  |
| 13 | rs9519862 | 106,588,926 | A | C | 0.04 | 0.03 | intergenic | LINC00343;LINC00460 | dist=174781;dist=439986 |  |
| 13 | rs7336109 | 106,589,141 | C | T | 0.04 | 0.04 | intergenic | LINC00343;LINC00460 | dist=174996;dist=439771 |  |
| 13 | rs7319510 | 106,598,677 | T | C | 0.05 | 0.02 | intergenic | LINC00343;LINC00460 | dist=184532;dist=430235 |  |
| 13 | rs367312 | 114,930,195 | G | A | 0.03 | 5.71E-05 | intergenic | RASA3;CDC16 | dist=32097;dist=70125 |  |
| 13 | rs6560933 | 114,937,294 | C | T | 0.04 | 5.98E-05 | intergenic | RASA3;CDC16 | dist=39196;dist=63026 |  |
| 13 | rs7995986 | 114,938,802 | A | G | 0.02 | 6.10E-04 | intergenic | RASA3;CDC16 | dist=40704;dist=61518 |  |
| 13 | rs10467349 | 114,939,441 | T | C | 0.02 | 6.00E-04 | intergenic | RASA3;CDC16 | dist=41343;dist=60879 |  |
| 13 | rs7327370 | 114,946,858 | A | G | 0.01 | 5.14E-04 | intergenic | RASA3;CDC16 | dist=48760;dist=53462 |  |
| 14 | rs11161050 | 29,733,102 | A | G | 0.04 | 0.05 | intergenic | LINC01551;PRKD1 | dist=469102;dist=312584 |  |
| 14 | rs10873584 | 29,733,121 | C | T | 0.03 | 0.05 | intergenic | LINC01551;PRKD1 | dist=469121;dist=312565 |  |
| 14 | rs1956239 | 29,734,439 | T | C | 0.04 | 0.05 | intergenic | LINC01551;PRKD1 | dist=470439;dist=311247 |  |
| 14 | rs10142289 | 45,031,726 | C | T | 6.06E-03 | 0.04 | intergenic | LOC105370473;LINC02302 | dist=24013;dist=200634 |  |
| 14 | rs10144890 | 45,032,097 | C | T | 5.60E-03 | 0.04 | intergenic | LOC105370473;LINC02302 | dist=24384;dist=200263 |  |
| 14 | rs1104969 | 45,046,941 | G | T | 0.01 | 0.01 | intergenic | LOC105370473;LINC02302 | dist=39228;dist=185419 |  |
| 14 | rs17117632 | 47,245,611 | C | T | 0.02 | 3.66E-03 | intergenic | RPL10L;MDGA2 | dist=124627;dist=63215 |  |
| 14 | rs2035857 | 47,247,399 | G | A | 0.02 | 6.80E-03 | intergenic | RPL10L;MDGA2 | dist=126415;dist=61427 |  |
| 14 | rs10135930 | 52,609,680 | G | A | 0.04 | 0.04 | intergenic | NID2;PTGDR | dist=73903;dist=124736 |  |
| 14 | rs2348075 | 58,853,140 | T | C | 0.01 | 3.13E-03 | intergenic | ARID4A;TOMM20L | dist=12549;dist=9508 |  |
| 14 | rs7150662 | 63,100,169 | C | T | 0.03 | 0.01 | intergenic | LINC00644;KCNH5 | dist=493478;dist=66013 |  |
| 14 | rs986201 | 66,536,914 | A | G | 0.04 | 0.01 | intergenic | FUT8;CCDC196 | dist=326075;dist=416175 |  |
| 14 | rs1958232 | 82,220,579 | A | G | 0.04 | 2.64E-03 | intergenic | LINC02311;LINC02301 | dist=11097;dist=888397 |  |
| 14 | rs7159249 | 90,193,978 | A | G | 0.01 | 0.03 | intergenic | FOXN3;EFCAB11 | dist=108469;dist=67036 |  |
| 14 | rs7158998 | 90,194,121 | G | A | 7.68E-04 | 0.02 | intergenic | FOXN3;EFCAB11 | dist=108612;dist=66893 |  |
| 14 | rs965950 | 97,034,805 | A | G | 0.02 | 4.26E-03 | intergenic | PAPOLA;LINC02299 | dist=1352;dist=172723 |  |
| 14 | rs11160344 | 97,035,680 | C | T | 9.98E-03 | 3.38E-03 | intergenic | PAPOLA;LINC02299 | dist=2227;dist=171848 |  |
| 14 | rs11848778 | 97,039,486 | C | T | 7.18E-03 | 2.62E-03 | intergenic | PAPOLA;LINC02299 | dist=6033;dist=168042 |  |
| 14 | rs17094877 | 97,494,975 | C | T | 0.04 | 0.04 | intergenic | LINC00618;LINC02304 | dist=83244;dist=126219 |  |
| 14 | rs11621035 | 99,157,487 | C | T | 0.04 | 0.05 | intergenic | LINC01550;C14orf177 | dist=713026;dist=20463 |  |
| 14 | rs11847580 | 102,157,773 | C | A | 0.03 | 6.76E-03 | intergenic | DIO3;LINC00239 | dist=127984;dist=39001 |  |
| 15 | rs12148122 | 53,468,486 | A | C | 8.98E-03 | 0.01 | intergenic | LINC02490;WDR72 | dist=46591;dist=337452 |  |
| 15 | rs578079 | 53,482,048 | A | C | 8.69E-03 | 0.01 | intergenic | LINC02490;WDR72 | dist=60153;dist=323890 |  |
| 15 | rs16965675 | 53,493,843 | T | C | 0.01 | 0.01 | intergenic | LINC02490;WDR72 | dist=71948;dist=312095 |  |
| 15 | rs16965678 | 53,496,496 | A | C | 9.08E-03 | 0.01 | intergenic | LINC02490;WDR72 | dist=74601;dist=309442 |  |
| 15 | rs566369 | 53,508,592 | A | G | 0.03 | 8.69E-03 | intergenic | LINC02490;WDR72 | dist=86697;dist=297346 |  |
| 15 | rs482541 | 53,509,194 | A | G | 0.03 | 8.60E-03 | intergenic | LINC02490;WDR72 | dist=87299;dist=296744 |  |
| 15 | rs2725629 | 54,194,365 | A | G | 2.60E-03 | 0.05 | intergenic | WDR72;UNC13C | dist=142505;dist=76273 |  |
| 15 | rs8041529 | 55,118,892 | C | T | 0.04 | 3.86E-03 | intergenic | UNC13C;LOC105370829 | dist=193255;dist=230053 |  |
| 15 | rs430610 | 68,198,622 | A | G | 0.05 | 2.40E-03 | intergenic | RNU6-9;PIAS1 | dist=66239;dist=147895 |  |
| 15 | rs448720 | 68,198,911 | T | C | 0.05 | 2.56E-03 | intergenic | RNU6-9;PIAS1 | dist=66528;dist=147606 |  |
| 15 | rs17828399 | 73,151,979 | T | C | 0.04 | 0.04 | intergenic | ADPGK-AS1;NEO1 | dist=61439;dist=192825 |  |
| 15 | rs7162423 | 74,052,756 | T | C | 0.01 | 1.47E-03 | intergenic | INSYN1;INSYN1-AS1 | dist=8045;dist=7671 |  |
| 15 | rs11858528 | 95,939,651 | G | T | 0.03 | 0.04 | intergenic | LINC01197;LINC00924 | dist=69322;dist=36671 |  |
| 16 | rs8048890 | 8,470,353 | G | A | 0.02 | 0.04 | intergenic | RBFOX1;TMEM114 | dist=707011;dist=117218 |  |
| 16 | rs12923941 | 8,565,722 | T | C | 0.01 | 0.04 | intergenic | RBFOX1;TMEM114 | dist=802380;dist=21849 |  |
| 16 | rs8047849 | 17,583,593 | C | T | 0.02 | 0.05 | intergenic | XYLT1;NPIPA8 | dist=18776;dist=828183 |  |
| 16 | rs11642116 | 23,797,412 | C | A | 0.05 | 1.54E-05 | intergenic | CHP2;PRKCB | dist=27156;dist=49892 |  |
| 16 | rs17764539 | 49,040,995 | A | G | 7.80E-03 | 0.05 | intergenic | N4BP1;CBLN1 | dist=396904;dist=270834 |  |
| 16 | rs746048 | 49,042,391 | T | C | 0.01 | 0.04 | intergenic | N4BP1;CBLN1 | dist=398300;dist=269438 |  |
| 16 | rs2908881 | 49,075,648 | C | T | 0.03 | 9.75E-05 | intergenic | N4BP1;CBLN1 | dist=431557;dist=236181 |  |
| 16 | rs2160494 | 49,078,680 | A | G | 0.03 | 3.92E-05 | intergenic | N4BP1;CBLN1 | dist=434589;dist=233149 |  |
| 16 | rs7188866 | 54,385,688 | C | T | 0.03 | 0.01 | intergenic | IRX3;LINC02140 | dist=64989;dist=14231 |  |
| 16 | rs13380644 | 59,578,821 | T | C | 7.29E-03 | 0.02 | intergenic | GOT2;APOOP5 | dist=810601;dist=209224 |  |
| 16 | rs11645366 | 62,377,153 | T | C | 0.03 | 0.05 | intergenic | CDH8;NONE | dist=306811;dist=NONE |  |
| 16 | rs1364063 | 69,588,572 | C | T | 2.85E-03 | 4.10E-03 | intergenic | CYB5B;MIR1538 | dist=88405;dist=11139 |  |
| 16 | rs1383361 | 73,674,216 | T | C | 0.02 | 0.03 | intergenic | LINC01568;LOC101928035 | dist=218921;dist=552075 |  |
| 16 | rs4369705 | 76,640,703 | T | C | 0.02 | 0.03 | intergenic | CNTNAP4;LINC02125 | dist=46051;dist=28192 |  |
| 16 | rs11644400 | 79,928,186 | C | T | 0.02 | 2.40E-04 | intergenic | LINC01229;LOC102724084 | dist=92946;dist=261669 |  |
| 16 | rs16956940 | 82,303,212 | C | T | 0.02 | 2.87E-03 | intergenic | MPHOSPH6;CDH13 | dist=99383;dist=357362 |  |
| 16 | rs9938149 | 88,331,640 | C | A | 0.04 | 0.05 | intergenic | LINC02182;ZNF469 | dist=102817;dist=117727 |  |
| 17 | rs1851267 | 25,397,308 | C | T | 0.05 | 0.03 | intergenic | NONE;LOC105371703 | dist=NONE;dist=2107 | Y |
| 17 | rs4795519 | 25,541,278 | A | C | 0.04 | 0.03 | intergenic | LOC105371703;MIR4522 | dist=125011;dist=79658 |  |
| 17 | rs11650043 | 46,027,852 | A | G | 0.03 | 0.04 | intergenic | PNPO;PRR15L | dist=1178;dist=1482 |  |
| 17 | rs9906150 | 69,074,779 | A | G | 0.03 | 1.85E-03 | intergenic | KCNJ2;CASC17 | dist=898596;dist=19136 |  |
| 18 | rs7238906 | 4,545,834 | C | T | 0.02 | 0.02 | intergenic | DLGAP1;LINC01892 | dist=90527;dist=535347 |  |
| 18 | rs11872163 | 4,582,624 | G | A | 0.03 | 4.71E-04 | intergenic | DLGAP1;LINC01892 | dist=127317;dist=498557 |  |
| 18 | rs9950268 | 4,925,256 | G | A | 0.04 | 0.03 | intergenic | DLGAP1;LINC01892 | dist=469949;dist=155925 |  |
| 18 | rs11660230 | 5,632,969 | A | C | 0.02 | 0.04 | intergenic | EPB41L3;MIR3976HG | dist=3979;dist=115849 |  |
| 18 | rs17203754 | 22,261,683 | A | G | 4.90E-03 | 0.03 | intergenic | LINC01915;LOC105372028 | dist=19521;dist=44062 |  |
| 18 | rs1440833 | 27,389,543 | C | T | 9.18E-03 | 1.08E-03 | intergenic | CDH2;MIR302F | dist=1632449;dist=489333 |  |
| 18 | rs9959618 | 35,233,241 | T | C | 0.05 | 0.04 | intergenic | CELF4;MIR4318 | dist=87480;dist=3857 |  |
| 18 | rs1496814 | 38,953,812 | G | A | 0.04 | 4.92E-04 | intergenic | LINC01477;KC6 | dist=1274615;dist=106424 |  |
| 18 | rs16974577 | 38,963,000 | T | C | 0.02 | 5.42E-04 | intergenic | LINC01477;KC6 | dist=1283803;dist=97236 |  |
| 18 | rs9304248 | 39,146,838 | A | G | 0.02 | 0.01 | intergenic | KC6;PIK3C3 | dist=46197;dist=388361 |  |
| 18 | rs17656030 | 39,294,665 | G | A | 0.05 | 2.55E-06 | intergenic | KC6;PIK3C3 | dist=194024;dist=240534 |  |
| 18 | rs930189 | 39,451,865 | T | C | 0.02 | 0.04 | intergenic | KC6;PIK3C3 | dist=351224;dist=83334 |  |
| 18 | rs4514760 | 41,331,128 | A | C | 0.02 | 0.05 | intergenic | SYT4;LINC01478 | dist=473673;dist=572272 |  |
| 18 | rs8087439 | 57,642,596 | T | C | 0.02 | 1.13E-03 | intergenic | PMAIP1;MC4R | dist=71059;dist=395699 |  |
| 18 | rs1788590 | 71,972,680 | G | A | 9.63E-03 | 0.02 | intergenic | CYB5A;C18orf63 | dist=13482;dist=10394 | Y |
| 19 | rs10427083 | 18,550,276 | C | A | 0.04 | 0.02 | intergenic | ISYNA1;ELL | dist=1333;dist=3197 |  |
| 19 | rs4805532 | 30,736,785 | T | C | 0.05 | 0.04 | intergenic | URI1;ZNF536 | dist=229266;dist=126506 |  |
| 19 | rs2546056 | 35,378,725 | T | C | 0.02 | 0.02 | intergenic | LINC01801;LINC00904 | dist=54952;dist=4208 |  |
| 19 | rs11668752 | 56,094,578 | A | G | 0.01 | 1.53E-04 | intergenic | ZNF579;FIZ1 | dist=2364;dist=8164 |  |
| 19 | rs310465 | 56,123,746 | T | C | 8.12E-03 | 0.02 | intergenic | ZNF524;ZNF865 | dist=9242;dist=1213 |  |
| 20 | rs10485749 | 11,426,730 | G | A | 0.01 | 0.01 | intergenic | LOC339593;LINC00687 | dist=172699;dist=363905 |  |
| 20 | rs6040692 | 11,430,363 | A | G | 7.74E-03 | 0.01 | intergenic | LOC339593;LINC00687 | dist=176332;dist=360272 |  |
| 20 | rs6033146 | 11,437,119 | C | T | 4.13E-03 | 0.01 | intergenic | LOC339593;LINC00687 | dist=183088;dist=353516 |  |
| 20 | rs2180099 | 11,439,252 | A | C | 1.12E-03 | 0.01 | intergenic | LOC339593;LINC00687 | dist=185221;dist=351383 |  |
| 20 | rs6104763 | 11,500,108 | G | A | 0.02 | 0.03 | intergenic | LOC339593;LINC00687 | dist=246077;dist=290527 |  |
| 20 | rs6132084 | 18,564,374 | G | T | 0.04 | 1.13E-04 | intergenic | SMIM26;DTD1 | dist=14119;dist=4324 |  |
| 20 | rs11700231 | 24,039,191 | G | A | 0.04 | 0.03 | intergenic | GGTLC1;LINC01721 | dist=69775;dist=141212 |  |
| 21 | rs2825560 | 20,773,423 | C | T | 0.05 | 8.16E-03 | intergenic | MIR548XHG;LINC01683 | dist=641293;dist=492170 |  |
| 21 | rs1551588 | 28,982,407 | A | G | 0.02 | 0.01 | intergenic | ADAMTS5;LINC00113 | dist=643000;dist=112291 |  |
| 21 | rs17203484 | 31,376,662 | A | C | 0.02 | 0.01 | intergenic | GRIK1;CLDN17 | dist=64292;dist=161223 |  |
| 21 | rs9976786 | 31,378,176 | A | G | 0.03 | 0.02 | intergenic | GRIK1;CLDN17 | dist=65806;dist=159709 |  |
| 21 | rs2226295 | 34,382,282 | G | T | 0.05 | 0.04 | intergenic | LINC01690;OLIG2 | dist=49410;dist=15961 |  |
| 21 | rs2835333 | 37,673,206 | C | T | 0.01 | 0.04 | intergenic | DOP1B;MORC3 | dist=6634;dist=19281 |  |
| 21 | rs2839642 | 44,602,306 | C | T | 0.04 | 0.02 | intergenic | CRYAA;LINC00322 | dist=9386;dist=139783 |  |
| 22 | rs5763140 | 29,718,321 | T | C | 0.05 | 0.01 | intergenic | RASL10A;AP1B1 | dist=6635;dist=5348 |  |
| 22 | rs2527344 | 30,111,624 | T | C | 0.02 | 0.04 | intergenic | NF2;CABP7 | dist=17035;dist=4368 |  |
| 22 | rs2527345 | 30,112,226 | T | C | 9.46E-03 | 0.04 | intergenic | NF2;CABP7 | dist=17637;dist=3766 |  |
| 22 | rs132905 | 41,799,106 | C | A | 0.04 | 1.04E-04 | intergenic | TEF;TOB2 | dist=3774;dist=30392 |  |
| 22 | rs132915 | 41,805,782 | T | C | 0.02 | 9.93E-05 | intergenic | TEF;TOB2 | dist=10450;dist=23716 |  |
| 22 | rs202664 | 41,813,886 | C | T | 0.03 | 5.01E-05 | intergenic | TEF;TOB2 | dist=18554;dist=15612 |  |

Note: Based on NCBI RefSeq GRCh37. Not in IB: Not included in PRS calculation in Indiana Biobank sample.
