# Supplementary material for: Gene-based polygenic risk scores analysis of alcohol use disorder in African Americans
Source: Transl Psychiatry. 2022 Jul 5;12:266. doi: 10.1038/s41398-022-02029-2 (PMC9256707; doi:10.1038/s41398-022-02029-2)
Supplement: Supplementary file 4 — PRSgene calculated using different window sizes. [file 41398_2022_2029_MOESM4_ESM.docx]

**Table S4**: PRS_gene_ calculated using different window sizes.

| Population | Window size | cohort | Beta | Stderr | P-Value | -logP | # Variants |
| --- | --- | --- | --- | --- | --- | --- | --- |
| AA | 1kb | All | 0.17 | 0.03 | 3.55E-08 | 7.45 | 858 |
| AA | 1kb | COGA | 0.15 | 0.04 | 9.67E-04 | 3.01 | 858 |
| AA | 1kb | SAGE | 0.18 | 0.07 | 6.27E-03 | 2.20 | 858 |
| AA | 1kb | YalePenn | 0.21 | 0.05 | 7.61E-05 | 4.12 | 858 |
| AA | 10kb | All | 0.17 | 0.03 | 7.81E-08 | 7.11 | 962 |
| AA | 10kb | COGA | 0.14 | 0.04 | 1.63E-03 | 2.79 | 962 |
| AA | 10kb | SAGE | 0.19 | 0.07 | 4.71E-03 | 2.33 | 962 |
| AA | 10kb | YalePenn | 0.21 | 0.05 | 1.37E-04 | 3.86 | 962 |
| AA | 25kb | All | 0.17 | 0.03 | 5.77E-08 | 7.24 | 1,061 |
| AA | 25kb | COGA | 0.14 | 0.04 | 1.68E-03 | 2.77 | 1,061 |
| AA | 25kb | SAGE | 0.20 | 0.07 | 3.12E-03 | 2.51 | 1,061 |
| AA | 25kb | YalePenn | 0.21 | 0.05 | 1.67E-04 | 3.78 | 1,061 |
| AA | 50kb | All | 0.18 | 0.03 | 4.86E-09 | 8.31 | 1,156 |
| AA | 50kb | COGA | 0.16 | 0.04 | 4.16E-04 | 3.38 | 1,156 |
| AA | 50kb | SAGE | 0.21 | 0.07 | 2.22E-03 | 2.65 | 1,156 |
| AA | 50kb | YalePenn | 0.21 | 0.05 | 1.05E-04 | 3.98 | 1,156 |
| AA | 100kb | All | 0.17 | 0.03 | 2.45E-08 | 7.61 | 1,267 |
| AA | 100kb | COGA | 0.16 | 0.04 | 3.87E-04 | 3.41 | 1,267 |
| AA | 100kb | SAGE | 0.20 | 0.07 | 3.83E-03 | 2.42 | 1,267 |
| AA | 100kb | YalePenn | 0.19 | 0.05 | 4.49E-04 | 3.35 | 1,267 |
| AA | 250kb | All | 0.16 | 0.03 | 1.16E-07 | 6.94 | 1,419 |
| AA | 250kb | COGA | 0.14 | 0.04 | 1.25E-03 | 2.90 | 1,419 |
| AA | 250kb | SAGE | 0.20 | 0.07 | 4.63E-03 | 2.33 | 1,419 |
| AA | 250kb | YalePenn | 0.19 | 0.06 | 5.78E-04 | 3.24 | 1,419 |
| AA | 500kb | All | 0.16 | 0.03 | 2.66E-07 | 6.58 | 1,506 |
| AA | 500kb | COGA | 0.14 | 0.04 | 1.58E-03 | 2.80 | 1,506 |
| AA | 500kb | SAGE | 0.21 | 0.07 | 3.15E-03 | 2.50 | 1,506 |
| AA | 500kb | YalePenn | 0.18 | 0.06 | 1.51E-03 | 2.82 | 1,506 |
| AA | 1Mb | All | 0.16 | 0.03 | 1.77E-07 | 6.75 | 1,530 |
| AA | 1Mb | COGA | 0.14 | 0.04 | 1.41E-03 | 2.85 | 1,530 |
| AA | 1Mb | SAGE | 0.21 | 0.07 | 2.61E-03 | 2.58 | 1,530 |
| AA | 1Mb | YalePenn | 0.19 | 0.06 | 1.02E-03 | 2.99 | 1,530 |
| AA | 50Mb | All | 0.16 | 0.03 | 1.85E-07 | 6.73 | 1,533 |
| AA | 50Mb | COGA | 0.14 | 0.04 | 1.54E-03 | 2.81 | 1,533 |
| AA | 50Mb | SAGE | 0.21 | 0.07 | 2.40E-03 | 2.62 | 1,533 |
| AA | 50Mb | YalePenn | 0.19 | 0.06 | 1.02E-03 | 2.99 | 1,533 |
| AA | 100Mb | All | 0.16 | 0.03 | 1.85E-07 | 6.73 | 1,533 |
| AA | 100Mb | COGA | 0.14 | 0.04 | 1.54E-03 | 2.81 | 1,533 |
| AA | 100Mb | SAGE | 0.21 | 0.07 | 2.40E-03 | 2.62 | 1,533 |
| AA | 100Mb | YalePenn | 0.19 | 0.06 | 1.02E-03 | 2.99 | 1,533 |
| EA | 1kb | IB | 0.11 | 0.05 | 0.02 | 1.63 | 847 |
| EA | 10kb | IB | 0.11 | 0.05 | 0.02 | 1.77 | 950 |
| EA | 25kb | IB | 0.11 | 0.05 | 0.02 | 1.76 | 1048 |
| EA | 50kb | IB | 0.12 | 0.05 | 0.01 | 1.92 | 1140 |
| EA | 100kb | IB | 0.11 | 0.05 | 0.01 | 1.84 | 1251 |
| EA | 250kb | IB | 0.09 | 0.05 | 0.05 | 1.29 | 1403 |
| EA | 500kb | IB | 0.08 | 0.05 | 0.09 | 1.03 | 1490 |
| EA | 1Mb | IB | 0.09 | 0.05 | 0.06 | 1.20 | 1511 |
| EA | 50Mb | IB | 0.09 | 0.05 | 0.06 | 1.22 | 1513 |
| EA | 100Mb | IB | 0.09 | 0.05 | 0.06 | 1.22 | 1513 |
