# Supplementary material for: Gene-based polygenic risk scores analysis of alcohol use disorder in African Americans
Source: Transl Psychiatry. 2022 Jul 5;12:266. doi: 10.1038/s41398-022-02029-2 (PMC9256707; doi:10.1038/s41398-022-02029-2)
Supplement: Supplementary file 5 — Gene ontology enrichment analysis results. [file 41398_2022_2029_MOESM5_ESM.docx]

**Table S5**: Gene ontology enrichment analysis results.

| GO biological process | # genes in biological process | # of genes mapped | expected # of mapped genes | over (+) /under (-) representation | fold Enrichment | P-value | FDR | enriched genes |
| --- | --- | --- | --- | --- | --- | --- | --- | --- |
| biological adhesion (GO:0022610) | 958 | 43 | 16.65 | + | 2.58 | 2.74E-08 | 2.16E-04 | *NLGN1,TENM2,SPON1,TNXB,NTM,LEF1,STAB2,NRXN2,IL1RAP,F11R,LPP,NTN1,PTPRF,CDH7,PODXL,TNN,CHST10,RAC2,ABL2,SLC39A8,EPHB1,OPCML,P2RY12,GRID2,CADM1,FIBCD1,LSAMP,USH2A,DLG2,PTPRC,CTTN,CDH12,CNTN2,COL8A1,PKN2,CNTN4,MFGE8,CDH18,JCAD,METAP1,DSC3,ADGRL3* |
| cell adhesion (GO:0007155) | 952 | 43 | 16.55 | + | 2.6 | 2.31E-08 | 3.63E-04 | *NLGN1,TENM2,SPON1,TNXB,NTM,LEF1,STAB2,NRXN2,IL1RAP,F11R,LPP,NTN1,PTPRF,CDH7,PODXL,TNN,CHST10,RAC2,ABL2,SLC39A8,EPHB1,OPCML,P2RY12,GRID2,CADM1,FIBCD1,LSAMP,USH2A,DLG2,PTPRC,CTTN,CDH12,CNTN2,COL8A1,PKN2,CNTN4,MFGE8,CDH18,JCAD,METAP1,DSC3,ADGRL3* |
| cell-cell adhesion (GO:0098609) | 515 | 26 | 8.95 | + | 2.9 | 2.42E-06 | 4.75E-03 | *NLGN1,TENM2,TNXB,LEF1,NRXN2,IL1RAP,F11R,LPP,NTN1,PTPRF,CDH7,RAC2,SLC39A8,P2RY12,GRID2,CADM1,DLG2,PTPRC,CDH12,CNTN2,CNTN4,CDH18,METAP1,DSC3,ADGRL3* |
| regulation of trans-synaptic signaling (GO:0099177) | 432 | 24 | 7.51 | + | 3.2 | 1.24E-06 | 4.89E-03 | *PRRT1,PRKN,GRID2,NLGN1,TSHZ3,HOMER1,DCC,SLC4A10,ITPR3,GRIN2B,RGS4,GRM5,PREPL,GRM7,TEP1,TMEM108,CNTN2,MAPT,CNTN4,PLCB1,DRD2,ERC2,EIF4E,EPHB1* |
| neuron differentiation (GO:0030182) | 1008 | 40 | 17.52 | + | 2.28 | 1.98E-06 | 5.18E-03 | *NLGN1,TENM2,NTM,LRP2,EFNA5,NTN1,GIGYF2,PTPRF,SALL1,RPS6KA5,GRM7,ERBB4,TNN,DZANK1,TMEM108,PLXNA2,RAC2,PITX2,DRD2,EPHB1,OPCML,GRID2,PRKCI,EPHA7,SLC12A5,ANKRD27,MAP1S,DCC,SLC4A10,TRAPPC9,USH2A,PBX1,CTTN,NFIB,TEP1,RPL24,CNTN2,WDPCP,MAPT,CNTN4* |
| cellular protein metabolic process (GO:0044267) | 3219 | 91 | 55.96 | + | 1.63 | 2.39E-06 | 5.36E-03 | *USP34,GALNT10,PTPDC1,PTPRF,PTPRG,DDA1,RPS6KA5,MAN1A2,RSRC1,NARS2,ARIH2,MACROD1,SLC39A8,MACROD2,LARGE1,EPHB1,PCMTD1,RARS2,PRKCI,EPHA7,DESI2,VPS37A,KSR2,VRK2,DNAJC3,LARS2,ILF3,BRMS1,TEP1,RPL24,YME1L1,MAPT,MAN1B1,ANKK1,ST6GALNAC3,PRKN,USP15,TNKS,LEF1,MTTP,STK39,PRDM14,DENND3,RNF5,ASB18,GIGYF2,ADH5,TTN,FBXO40,APH1B,ERBB4,ZSWIM8,ABL2,PSMF1,SMYD3,UGGT2,PIBF1,UGGT1,P4HTM,EIF4E,TRPM6,CAMK2G,DTD1,IP6K3,PRRT1,KDM4B,HACE1,BRPF1,EGF,FANCL,ZBTB16,NEK7,VEGFB,IRF2BP1,GTF2H1,WWP2,GNMT,CNOT4,MSRA,EEF1AKMT4,PTPRC,AGBL2,MKRN2,ERCC8,EIF3H,PKN2,GALNTL6,METAP1,FGFR1* |
| nervous system development (GO:0007399) | 2193 | 69 | 38.12 | + | 1.81 | 1.73E-06 | 5.44E-03 | *TENM2,CHD8,PTPRF,PTPRG,RGS4,SALL1,RPS6KA5,GRM7,TNN,ADGRA2,SALL4,TMEM108,RAC2,PITX2,EPHB1,GRID2,RBFOX1,PRKCI,EPHA7,MAP1S,DCC,MAGI2,NAV2,CTTN,TEP1,RPL24,WDPCP,MAPT,PLCB1,PRKN,NLGN1,NTM,LEF1,NRXN2,LRP2,EFNA5,NTN1,GIGYF2,FSTL4,AK8,ATXN1,ERBB4,DZANK1,PLXNA2,DRD2,ASIC2,CAMK2G,KIAA0319,OPCML,P2RY12,DNAH11,SLC12A5,ANKRD27,ZBTB16,SLC4A10,LSAMP,MTHFR,TRAPPC9,ATP2B1,USH2A,GRIN2B,PBX1,NFIB,CNTN2,CNTN4,SCN2A,SERINC5,FGFR1,ADGRL3* |
| behavior (GO:0007610) | 558 | 27 | 9.7 | + | 2.78 | 3.28E-06 | 5.73E-03 | *PRKN,CHD8,NRXN2,ITPR3,GIGYF2,GPR176,GRM5,ATXN1,APH1B,ABL2,DRD2,EIF4E,CSMD1,PRRT1,DNAH11,SLC12A5,HOMER1,SLC4A10,NAV2,OXR1,GRIN2B,MYO15A,TEP1,CNTN2,MAPT,SCN2A,PLCB1* |
| modulation of chemical synaptic transmission (GO:0050804) | 431 | 24 | 7.49 | + | 3.2 | 1.20E-06 | 6.28E-03 | *PRRT1,PRKN,GRID2,NLGN1,TSHZ3,HOMER1,DCC,SLC4A10,ITPR3,GRIN2B,RGS4,GRM5,PREPL,GRM7,TEP1,TMEM108,CNTN2,MAPT,CNTN4,PLCB1,DRD2,ERC2,EIF4E,EPHB1* |
| protein modification process (GO:0036211) | 2610 | 77 | 45.37 | + | 1.7 | 4.43E-06 | 6.32E-03 | *USP34,GALNT10,PTPDC1,PTPRF,PTPRG,DDA1,RPS6KA5,MAN1A2,RSRC1,ARIH2,MACROD1,SLC39A8,MACROD2,LARGE1,EPHB1,PCMTD1,PRKCI,EPHA7,DESI2,KSR2,VRK2,ILF3,BRMS1,TEP1,MAPT,MAN1B1,ANKK1,ST6GALNAC3,PRKN,USP15,TNKS,LEF1,MTTP,STK39,PRDM14,RNF5,ASB18,ADH5,TTN,FBXO40,ERBB4,ZSWIM8,ABL2,SMYD3,UGGT2,PIBF1,UGGT1,P4HTM,TRPM6,CAMK2G,IP6K3,PRRT1,KDM4B,HACE1,BRPF1,EGF,FANCL,ZBTB16,NEK7,VEGFB,IRF2BP1,GTF2H1,WWP2,GNMT,CNOT4,MSRA,EEF1AKMT4,PTPRC,AGBL2,MKRN2,ERCC8,PKN2,GALNTL6,METAP1,FGFR1* |
| cellular protein modification process (GO:0006464) | 2610 | 77 | 45.37 | + | 1.7 | 4.43E-06 | 6.96E-03 | *USP34,GALNT10,PTPDC1,PTPRF,PTPRG,DDA1,RPS6KA5,MAN1A2,RSRC1,ARIH2,MACROD1,SLC39A8,MACROD2,LARGE1,EPHB1,PCMTD1,PRKCI,EPHA7,DESI2,KSR2,VRK2,ILF3,BRMS1,TEP1,MAPT,MAN1B1,ANKK1,ST6GALNAC3,PRKN,USP15,TNKS,LEF1,MTTP,STK39,PRDM14,RNF5,ASB18,ADH5,TTN,FBXO40,ERBB4,ZSWIM8,ABL2,SMYD3,UGGT2,PIBF1,UGGT1,P4HTM,TRPM6,CAMK2G,IP6K3,PRRT1,KDM4B,HACE1,BRPF1,EGF,FANCL,ZBTB16,NEK7,VEGFB,IRF2BP1,GTF2H1,WWP2,GNMT,CNOT4,MSRA,EEF1AKMT4,PTPRC,AGBL2,MKRN2,ERCC8,PKN2,GALNTL6,METAP1,FGFR1* |
| macromolecule modification (GO:0043412) | 2825 | 81 | 49.11 | + | 1.65 | 6.41E-06 | 7.19E-03 | *USP34,GALNT10,PTPDC1,PTPRF,PTPRG,DDA1,ALKBH5,RPS6KA5,MAN1A2,RSRC1,ARIH2,MACROD1,SLC39A8,MACROD2,LARGE1,METTL15,EPHB1,PCMTD1,PRKCI,EPHA7,DESI2,KSR2,VRK2,ILF3,BRMS1,TEP1,MAPT,MAN1B1,ANKK1,ST6GALNAC3,FTO,PRKN,USP15,TNKS,LEF1,MTTP,STK39,PRDM14,RNF5,ASB18,ADH5,TTN,FBXO40,ERBB4,ZSWIM8,ABL2,SMYD3,ZC3H13,UGGT2,PIBF1,UGGT1,P4HTM,TRPM6,CAMK2G,IP6K3,PRRT1,KDM4B,HACE1,BRPF1,EGF,FANCL,ZBTB16,NEK7,VEGFB,IRF2BP1,GTF2H1,WWP2,GNMT,CNOT4,MSRA,EEF1AKMT4,PTPRC,AGBL2,MKRN2,ERCC8,PKN2,GALNTL6,METAP1,FGFR1* |
| generation of neurons (GO:0048699) | 1237 | 45 | 21.5 | + | 2.09 | 5.57E-06 | 7.30E-03 | *NLGN1,TENM2,NTM,LEF1,LRP2,EFNA5,NTN1,GIGYF2,PTPRF,FSTL4,SALL1,RPS6KA5,GRM7,ERBB4,TNN,DZANK1,TMEM108,PLXNA2,RAC2,PITX2,DRD2,EPHB1,KIAA0319,OPCML,GRID2,PRKCI,EPHA7,SLC12A5,ANKRD27,MAP1S,DCC,SLC4A10,TRAPPC9,USH2A,PBX1,CTTN,NFIB,TEP1,RPL24,CNTN2,WDPCP,MAPT,CNTN4,FGFR1,ADGRL3* |
| synapse organization (GO:0050808) | 289 | 18 | 5.02 | + | 3.58 | 6.29E-06 | 7.61E-03 | *PRRT1,GRID2,NLGN1,NRXN2,KSR2,IL1RAP,PTPRF,GRM5,CTTN,ERBB4,TEP1,TMEM108,CNTN2,MAPT,DRD2,ERC2,EPHB1,ADGRL3* |
| neurogenesis (GO:0022008) | 1361 | 47 | 23.66 | + | 1.99 | 9.74E-06 | 1.02E-02 | *NLGN1,TENM2,NTM,LEF1,LRP2,EFNA5,NTN1,GIGYF2,PTPRF,FSTL4,SALL1,RPS6KA5,GRM7,ERBB4,TNN,DZANK1,TMEM108,PLXNA2,RAC2,PITX2,DRD2,EPHB1,KIAA0319,OPCML,P2RY12,GRID2,PRKCI,EPHA7,SLC12A5,ANKRD27,MAP1S,DCC,SLC4A10,TRAPPC9,NAV2,USH2A,PBX1,CTTN,NFIB,TEP1,RPL24,CNTN2,WDPCP,MAPT,CNTN4,FGFR1,ADGRL3* |
| protein metabolic process (GO:0019538) | 3807 | 101 | 66.18 | + | 1.53 | 1.03E-05 | 1.02E-02 | *PREP,GALNT10,PTPDC1,HS6ST3,RPS6KA5,NARS2,MACROD1,SLC39A8,MACROD2,LARGE1,EPHB1,RARS2,PRKCI,EPHA7,VRK2,LARS2,ILF3,PREPL,BRMS1,TEP1,RPL24,YME1L1,ANKK1,TNKS,MTTP,STK39,PRDM14,GIGYF2,ADH5,FBXO40,APH1B,CHST10,ABL2,TSPAN5,PSMF1,SMYD3,PIBF1,TRPM6,IP6K3,PRRT1,HACE1,BRPF1,FANCL,ZBTB16,NEK7,IRF2BP1,CNOT4,MSRA,PTPRC,MKRN2,GALNTL6,FGFR1,USP34,PCSK6,PTPRF,PTPRG,DDA1,ADAMTS3,MAN1A2,RSRC1,ARIH2,PCMTD1,DESI2,VPS37A,KSR2,DNAJC3,MAPT,MAN1B1,ST6GALNAC3,PRKN,USP15,TMPRSS5,LEF1,DENND3,RNF5,ASB18,TTN,ERBB4,ZSWIM8,UGGT2,UGGT1,P4HTM,EIF4E,CAMK2G,DTD1,KDM4B,EGF,VEGFB,GTF2H1,WWP2,GNMT,TSPAN15,EEF1AKMT4,AGBL2,ERCC8,EIF3H,PKN2,METAP1* |
| regulation of synaptic plasticity (GO:0048167) | 189 | 14 | 3.29 | + | 4.26 | 1.12E-05 | 1.03E-02 | *PRRT1,GRID2,NLGN1,TSHZ3,SLC4A10,ITPR3,GRIN2B,GRM5,TEP1,CNTN2,MAPT,CNTN4,DRD2,ERC2* |
| neuron development (GO:0048666) | 810 | 33 | 14.08 | + | 2.34 | 1.50E-05 | 1.31E-02 | *NLGN1,TENM2,NTM,LRP2,EFNA5,NTN1,PTPRF,RPS6KA5,GRM7,TNN,DZANK1,TMEM108,PLXNA2,RAC2,DRD2,EPHB1,OPCML,PRKCI,EPHA7,SLC12A5,ANKRD27,MAP1S,DCC,SLC4A10,PBX1,CTTN,NFIB,TEP1,RPL24,CNTN2,WDPCP,MAPT,CNTN4* |
| cell junction organization (GO:0034330) | 477 | 23 | 8.29 | + | 2.77 | 1.87E-05 | 1.34E-02 | *PRRT1,GRID2,NLGN1,PRKCI,NRXN2,KSR2,IL1RAP,PTPRF,CDH7,GRM5,CTTN,ERBB4,TEP1,TMEM108,CDH12,CNTN2,PKN2,MAPT,DRD2,ERC2,EPHB1,CDH18,ADGRL3* |
| startle response (GO:0001964) | 27 | 6 | 0.47 | + | 12.78 | 1.79E-05 | 1.34E-02 | *PRKN,GRID2,TEP1,CHD8,DRD2,CSMD1* |
| synaptic signaling (GO:0099536) | 448 | 22 | 7.79 | + | 2.83 | 2.18E-05 | 1.37E-02 | *PRKN,CADPS2,GRID2,NLGN1,TENM2,SLC12A5,HOMER1,CHAT,NRXN2,INSYN1,IL1RAP,GRIN2B,GABRG3,GPR176,GRM5,DLG2,GRM7,TEP1,PLCB1,DRD2,ERC2,ASIC2* |
| trans-synaptic signaling (GO:0099537) | 416 | 21 | 7.23 | + | 2.9 | 2.28E-05 | 1.38E-02 | *PRKN,CADPS2,GRID2,NLGN1,TENM2,SLC12A5,HOMER1,CHAT,NRXN2,INSYN1,IL1RAP,GRIN2B,GABRG3,GPR176,GRM5,DLG2,GRM7,TEP1,DRD2,ERC2,ASIC2* |
| locomotion (GO:0040011) | 1195 | 42 | 20.77 | + | 2.02 | 2.13E-05 | 1.40E-02 | *TENM2,WWC1,LEF1,DNAH6,EFNA5,F11R,NTN1,PTPRF,LIMA1,RPS6KA5,ERBB4,PODXL,ADGRA2,PDE4B,PLXNA2,RAC2,PITX2,DRD2,EPHB1,KIAA0319,IP6K3,P2RY12,TTC12,PRKCI,EPHA7,DNAH11,DCC,VEGFB,GAB1,USH2A,PTPRC,CTTN,NFIB,TEP1,ELMO1,RPL24,CNTN2,WDPCP,PKN2,CNTN4,FGFR1,ADGRL3* |
| postsynaptic modulation of chemical synaptic transmission (GO:0099170) | 15 | 5 | 0.26 | + | 19.18 | 1.78E-05 | 1.40E-02 | *GRM5,DCC,PLCB1,DRD2,EIF4E* |
| cell morphogenesis (GO:0000902) | 675 | 29 | 11.73 | + | 2.47 | 1.70E-05 | 1.41E-02 | *NLGN1,TENM2,LEF1,PRDM14,LRP2,EFNA5,NTN1,CDH7,RPS6KA5,TNN,TMEM108,PLXNA2,RAC2,DRD2,EPHB1,EPHA7,ANKRD27,MAP1S,DCC,USH2A,CTTN,NFIB,TEP1,CDH12,RPL24,CNTN2,WDPCP,CNTN4,CDH18* |
| learning or memory (GO:0007611) | 257 | 16 | 4.47 | + | 3.58 | 2.07E-05 | 1.41E-02 | *PRRT1,PRKN,DNAH11,SLC12A5,NRXN2,ITPR3,GRIN2B,GRM5,ATXN1,TEP1,CNTN2,MAPT,SCN2A,PLCB1,DRD2,CSMD1* |
| neuron projection morphogenesis (GO:0048812) | 455 | 22 | 7.91 | + | 2.78 | 2.73E-05 | 1.59E-02 | *NLGN1,TENM2,EPHA7,ANKRD27,MAP1S,DCC,LRP2,EFNA5,NTN1,RPS6KA5,CTTN,NFIB,TNN,TEP1,TMEM108,PLXNA2,RAC2,RPL24,CNTN2,CNTN4,DRD2,EPHB1* |
| protein demannosylation (GO:0036507) | 17 | 5 | 0.3 | + | 16.92 | 2.94E-05 | 1.59E-02 | *MAN1A2,MAN1B1,UGGT2,RNF5,UGGT1* |
| plasma membrane bounded cell projection morphogenesis (GO:0120039) | 459 | 22 | 7.98 | + | 2.76 | 3.10E-05 | 1.62E-02 | *NLGN1,TENM2,EPHA7,ANKRD27,MAP1S,DCC,LRP2,EFNA5,NTN1,RPS6KA5,CTTN,NFIB,TNN,TEP1,TMEM108,PLXNA2,RAC2,RPL24,CNTN2,CNTN4,DRD2,EPHB1* |
| protein alpha-1,2-demannosylation (GO:0036508) | 17 | 5 | 0.3 | + | 16.92 | 2.94E-05 | 1.65E-02 | *MAN1A2,MAN1B1,UGGT2,RNF5,UGGT1* |
| cell projection morphogenesis (GO:0048858) | 463 | 22 | 8.05 | + | 2.73 | 3.50E-05 | 1.72E-02 | *NLGN1,TENM2,EPHA7,ANKRD27,MAP1S,DCC,LRP2,EFNA5,NTN1,RPS6KA5,CTTN,NFIB,TNN,TEP1,TMEM108,PLXNA2,RAC2,RPL24,CNTN2,CNTN4,DRD2,EPHB1* |
| central nervous system development (GO:0007417) | 1002 | 37 | 17.42 | + | 2.12 | 3.49E-05 | 1.77E-02 | *PRKN,CHD8,LEF1,LRP2,GIGYF2,AK8,PTPRG,RGS4,SALL1,ATXN1,ERBB4,ADGRA2,TMEM108,PLXNA2,PITX2,DRD2,ASIC2,EPHB1,P2RY12,GRID2,EPHA7,MAP1S,DCC,ZBTB16,SLC4A10,TRAPPC9,NAV2,ATP2B1,GRIN2B,PBX1,NFIB,TEP1,CNTN2,MAPT,CNTN4,PLCB1,ADGRL3* |
| cellular macromolecule metabolic process (GO:0044260) | 4442 | 111 | 77.21 | + | 1.44 | 4.25E-05 | 2.02E-02 | *GALNT10,PTPDC1,HS6ST3,RPS6KA5,SPATA22,NARS2,MACROD1,SLC39A8,MACROD2,LARGE1,EPHB1,RARS2,PRKCI,EPHA7,VRK2,POLRMT,LARS2,ILF3,BRMS1,TEP1,RPL24,YME1L1,ANKK1,RHNO1,FTO,TNKS,MTTP,GLIS3,STK39,PRDM14,GIGYF2,ADH5,FBXO40,APH1B,CHST10,ABL2,MCMBP,PSMF1,SMYD3,ZC3H13,DRD2,PIBF1,TRPM6,IP6K3,PRRT1,HACE1,BRPF1,FANCL,ZBTB16,NEK7,IRF2BP1,CNOT4,MSRA,PTPRC,CNOT7,MKRN2,CNTN2,GALNTL6,RAD9A,FGFR1,ELL,GBE1,USP34,PCSK6,PTPRF,PTPRG,DDA1,MAN1A2,RSRC1,ARIH2,METTL15,PCMTD1,DESI2,VPS37A,KSR2,DNAJC3,MAPT,MAN1B1,ST6GALNAC3,PRKN,NFAT5,USP15,LEF1,DENND3,RNF5,ASB18,TTN,ERBB4,ZSWIM8,UGGT2,UGGT1,P4HTM,EIF4E,CAMK2G,DTD1,KDM4B,EGF,VEGFB,GTF2H1,WWP2,GNMT,EEF1AKMT4,NFIB,AGBL2,ERCC8,EIF3H,PKN2,METAP1* |
| axon development (GO:0061564) | 375 | 19 | 6.52 | + | 2.91 | 5.33E-05 | 2.33E-02 | *TENM2,EPHA7,MAP1S,DCC,EFNA5,NTN1,RPS6KA5,GRM7,NFIB,TNN,TEP1,PLXNA2,RAC2,RPL24,CNTN2,MAPT,CNTN4,DRD2,EPHB1* |
| movement of cell or subcellular component (GO:0006928) | 1413 | 46 | 24.56 | + | 1.87 | 5.61E-05 | 2.38E-02 | *TENM2,WWC1,LEF1,DNAH6,EFNA5,F11R,NTN1,PTPRF,LIMA1,RPS6KA5,ERBB4,PODXL,ADGRA2,TMEM108,PDE4B,PLXNA2,RAC2,PITX2,DRD2,EPHB1,KIAA0319,P2RY12,TTC12,PRKCI,EPHA7,DNAH11,MAP1S,DCC,GAB1,USH2A,ADCY10,DLG2,PTPRC,CTTN,NFIB,MYO15A,TEP1,ELMO1,RPL24,CNTN2,WDPCP,PKN2,MAPT,CNTN4,FGFR1,ADGRL3* |
| ethanol oxidation (GO:0006069) | 9 | 4 | 0.16 | + | 25.57 | 5.30E-05 | 2.38E-02 | *ADH4,ADH1C,ADH1B,ADH5* |
| organonitrogen compound metabolic process (GO:1901564) | 4787 | 117 | 83.21 | + | 1.41 | 5.29E-05 | 2.44E-02 | *PREP,GALNT10,PTPDC1,HS6ST3,RPS6KA5,NARS2,MACROD1,SLC39A8,MACROD2,LARGE1,EPHB1,RARS2,PRKCI,EPHA7,VRK2,CPT1B,LARS2,ILF3,PREPL,BRMS1,TEP1,RPL24,YME1L1,ANKK1,TNKS,MTTP,CHAT,STK39,PRDM14,GIGYF2,ADH5,FBXO40,APH1B,CHST10,PNPO,ABL2,TSPAN5,PSMF1,SMYD3,DRD2,PIBF1,TRPM6,IP6K3,PRRT1,HACE1,BRPF1,FANCL,ADHFE1,ZBTB16,NEK7,IRF2BP1,MTHFR,CNOT4,MSRA,ADCY10,DLG2,PTPRC,MKRN2,ALDH18A1,GALNTL6,SERINC5,FGFR1,SLC44A1,USP34,PCSK6,ACSM2B,PTPRF,PTPRG,DDA1,ADAMTS3,MAN1A2,RSRC1,ARIH2,PDE4B,PCMTD1,DESI2,VPS37A,KSR2,ELOVL7,DNAJC3,MAPT,MAN1B1,ST6GALNAC3,PRKN,USP15,TMPRSS5,LEF1,DENND3,RNF5,ASB18,ADCY7,TTN,ERBB4,ZSWIM8,UGGT2,UGGT1,P4HTM,EIF4E,CAMK2G,DTD1,KDM4B,EGF,VEGFB,GTF2H1,WWP2,NMNAT2,GNMT,TSPAN15,EEF1AKMT4,AGBL2,ERCC8,EIF3H,PKN2,METAP1* |
| cell part morphogenesis (GO:0032990) | 483 | 22 | 8.4 | + | 2.62 | 6.38E-05 | 2.64E-02 | *NLGN1,TENM2,EPHA7,ANKRD27,MAP1S,DCC,LRP2,EFNA5,NTN1,RPS6KA5,CTTN,NFIB,TNN,TEP1,TMEM108,PLXNA2,RAC2,RPL24,CNTN2,CNTN4,DRD2,EPHB1* |
| regulation of plasma membrane bounded cell projection organization (GO:0120035) | 626 | 26 | 10.88 | + | 2.39 | 7.23E-05 | 2.92E-02 | *NLGN1,TENM2,NTN1,PTPRF,FSTL4,PTPRG,PODXL,PLXNA2,RAC2,ABL2,CAMK2G,KIAA0319,P2RY12,GRID2,PRKCI,EPHA7,ANKRD27,HOMER1,DCC,MAGI2,ZNF804A,GRIN2B,TEP1,CNTN2,WDPCP,MAPT* |
| anatomical structure morphogenesis (GO:0009653) | 2130 | 62 | 37.03 | + | 1.67 | 7.63E-05 | 3.00E-02 | *TENM2,STAB2,CDH7,SALL1,RPS6KA5,TNN,ADGRA2,SALL4,TMEM108,RAC2,PTGFRN,PITX2,EPHB1,GRID2,PRKCI,EPHA7,MAP1S,DCC,MAGI2,GAB1,HNF1B,CTTN,TEP1,CDH12,RPL24,COL8A1,WDPCP,ZFPM2,MFGE8,CDH18,FBN2,NLGN1,LEF1,PRDM14,LRP2,EFNA5,NTN1,TTN,ERBB4,PODXL,DZANK1,PLXNA2,DRD2,CSMD1,DNAH11,ANKRD27,EGF,ZBTB16,SLC4A10,VEGFB,MTHFR,NFATC1,USH2A,ESR1,PBX1,FLI1,NFIB,MYO15A,ALPL,CNTN2,CNTN4,FGFR1* |
| regulation of cell projection organization (GO:0031344) | 643 | 26 | 11.18 | + | 2.33 | 1.01E-04 | 3.88E-02 | *NLGN1,TENM2,NTN1,PTPRF,FSTL4,PTPRG,PODXL,PLXNA2,RAC2,ABL2,CAMK2G,KIAA0319,P2RY12,GRID2,PRKCI,EPHA7,ANKRD27,HOMER1,DCC,MAGI2,ZNF804A,GRIN2B,TEP1,CNTN2,WDPCP,MAPT* |
| cell development (GO:0048468) | 1623 | 50 | 28.21 | + | 1.77 | 1.06E-04 | 3.97E-02 | *TENM2,F11R,PTPRF,RPS6KA5,GRM7,TNN,ARIH2,TMEM108,RAC2,PITX2,EPHB1,PRKCI,EPHA7,MAP1S,DCC,MAGI2,CTTN,TEP1,RPL24,WDPCP,MAPT,NLGN1,NTM,LEF1,PRDM14,LRP2,EFNA5,NTN1,TTN,ERBB4,PODXL,DZANK1,PLXNA2,SMYD3,DRD2,OPCML,TTC12,SLC12A5,ANKRD27,HOMER1,ZBTB16,SLC4A10,USH2A,ESR1,PBX1,FLI1,PTPRC,NFIB,CNTN2,CNTN4* |
| cognition (GO:0050890) | 299 | 16 | 5.2 | + | 3.08 | 1.14E-04 | 3.97E-02 | *PRRT1,PRKN,DNAH11,SLC12A5,NRXN2,ITPR3,GRIN2B,GRM5,ATXN1,TEP1,CNTN2,MAPT,SCN2A,PLCB1,DRD2,CSMD1* |
| anterograde trans-synaptic signaling (GO:0098916) | 398 | 19 | 6.92 | + | 2.75 | 1.13E-04 | 4.02E-02 | *PRKN,CADPS2,GRID2,NLGN1,SLC12A5,HOMER1,CHAT,NRXN2,INSYN1,GRIN2B,GABRG3,GPR176,GRM5,DLG2,GRM7,TEP1,DRD2,ERC2,ASIC2* |
| neuron projection development (GO:0031175) | 651 | 26 | 11.32 | + | 2.3 | 1.20E-04 | 4.09E-02 | *NLGN1,TENM2,LRP2,EFNA5,NTN1,PTPRF,RPS6KA5,GRM7,TNN,TMEM108,PLXNA2,RAC2,DRD2,EPHB1,EPHA7,SLC12A5,ANKRD27,MAP1S,DCC,CTTN,NFIB,TEP1,RPL24,CNTN2,MAPT,CNTN4* |
| chemical synaptic transmission (GO:0007268) | 398 | 19 | 6.92 | + | 2.75 | 1.13E-04 | 4.11E-02 | *PRKN,CADPS2,GRID2,NLGN1,SLC12A5,HOMER1,CHAT,NRXN2,INSYN1,GRIN2B,GABRG3,GPR176,GRM5,DLG2,GRM7,TEP1,DRD2,ERC2,ASIC2* |
| regulation of synapse organization (GO:0050807) | 210 | 13 | 3.65 | + | 3.56 | 1.29E-04 | 4.22E-02 | *LINGO2,GRID2,NLGN1,EPHA7,HOMER1,ZNF804A,IL1RAP,GRIN2B,NTN1,TEP1,DRD2,ASIC2,EPHB1* |
| cell-cell signaling (GO:0007267) | 1030 | 36 | 17.9 | + | 2.01 | 1.28E-04 | 4.29E-02 | *PRKN,NLGN1,TENM2,TNKS,CHD8,USP34,LEF1,CHAT,NRXN2,IL1RAP,GPR176,GRM5,GRM7,GJA3,ADGRA2,DRD2,ERC2,BRD7,ASIC2,CAMK2G,CADPS2,TCF7L1,GRID2,SLC12A5,HOMER1,MAGI2,AXIN1,INSYN1,HNF1B,TMEM170B,GRIN2B,GABRG3,DLG2,TEP1,MAPT,PLCB1* |
| axonogenesis (GO:0007409) | 337 | 17 | 5.86 | + | 2.9 | 1.38E-04 | 4.42E-02 | *TENM2,EPHA7,MAP1S,DCC,EFNA5,NTN1,RPS6KA5,NFIB,TNN,TEP1,PLXNA2,RAC2,RPL24,CNTN2,CNTN4,DRD2,EPHB1* |
| protein deglycosylation (GO:0006517) | 25 | 5 | 0.43 | + | 11.51 | 1.42E-04 | 4.46E-02 | *MAN1A2,MAN1B1,UGGT2,RNF5,UGGT1* |
| central nervous system neuron differentiation (GO:0021953) | 184 | 12 | 3.2 | + | 3.75 | 1.47E-04 | 4.54E-02 | *GRID2,SALL1,ERBB4,DCC,NFIB,TEP1,SLC4A10,CNTN2,MAPT,DRD2,EPHB1,GIGYF2* |
| excitatory synapse assembly (GO:1904861) | 13 | 4 | 0.23 | + | 17.7 | 1.67E-04 | 4.96E-02 | *GRID2,NLGN1,TEP1,NRXN2* |
| prepulse inhibition (GO:0060134) | 13 | 4 | 0.23 | + | 17.7 | 1.67E-04 | 5.05E-02 | *GRID2,TEP1,CHD8,DRD2* |
| regulation of signaling (GO:0023051) | 3257 | 85 | 56.77 | + | 1.5 | 1.43E-04 | 4.50E-02 | *PRR5,FAM49B,CHD8,DENND1A,WWC1,USP34,F11R,PCSK6,DUSP16,AACS,RGS4,SPRED2,GRM5,SALL1,ADAMTS3,GRM7,TNN,ADGRA2,TMEM108,PDE4B,ERC2,LMCD1,EPHB1,RGS6,GRID2,PRKCI,EPHA7,RSAD2,DCC,MAGI2,AXIN1,KSR2,VRK2,TMEM170B,ELAPOR2,PREPL,CTTN,BANK1,TEP1,MAPT,PLCB1,JCAD,FBN2,PRKN,NFAT5,NLGN1,USP15,TSHZ3,TNKS,LEF1,STK39,PRDM14,DENND3,ITPR3,LRP2,EFNA5,FSTL4,ERBB4,ABL2,TSPAN5,DRD2,PIBF1,EIF4E,KIAA0319,SNX6,PRRT1,P2RY12,TCF7L1,HOMER1,EGF,UQCC2,SLC4A10,VEGFB,NFATC1,WWP2,ATP2B1,GRIN2B,ESR1,TSPAN15,PTPRC,CNOT7,SLC6A9,CNTN2,CNTN4,FGFR1* |
