# Supplementary material for: Gene-based polygenic risk scores analysis of alcohol use disorder in African Americans
Source: Transl Psychiatry. 2022 Jul 5;12:266. doi: 10.1038/s41398-022-02029-2 (PMC9256707; doi:10.1038/s41398-022-02029-2)
Supplement: Supplementary file 6 — Drug target genes. [file 41398_2022_2029_MOESM6_ESM.docx]

**Table S6**: Drug target genes.

| GenerefGene | MSH | Latest Phase |
| --- | --- | --- |
| *ESR1* | Acne Vulgaris | Approved |
| *DRD2* | Acromegaly | Approved |
| *P2RY12* | Acute Coronary Syndrome | Approved |
| *FGFR1* | Adrenal Gland Neoplasms | Phase I Clinical Trial |
| *DRD2* | Alcoholism | Phase III Clinical Trial |
| *GRM5* | Alcoholism | Phase II Clinical Trial |
| *PDE4B* | Alcoholism | Phase II Clinical Trial |
| *SLC6A9* | Alcoholism | Phase II Clinical Trial |
| *CHAT* | Alzheimer Disease | Phase II Clinical Trial |
| *PREP* | Alzheimer Disease | Phase II Clinical Trial |
| *DRD2* | Alzheimer Disease | Approved |
| *PDE4B* | Alzheimer Disease | Phase II Clinical Trial |
| *MAPT* | Alzheimer Disease | Phase III Clinical Trial |
| *SLC6A9* | Alzheimer Disease | Phase II Clinical Trial |
| *DRD2* | Amenorrhea | Approved |
| *PDE4B* | Amphetamine-Related Disorders | Phase II Clinical Trial |
| *DRD2* | Amyotrophic Lateral Sclerosis | Phase II Clinical Trial |
| *PDE4B* | Amyotrophic Lateral Sclerosis | Phase II Clinical Trial |
| *P2RY12* | Anemia, Sickle Cell | Phase III Clinical Trial |
| *P2RY12* | Angina, Unstable | Approved |
| *DRD2* | Anxiety | Phase III Clinical Trial |
| *GRM5* | Anxiety | Phase I Clinical Trial |
| *PDE4B* | Arthritis | Approved |
| *PDE4B* | Arthritis, Psoriatic | Approved |
| *PDE4B* | Arthritis, Rheumatoid | Phase II Clinical Trial |
| *DRD2* | Asthma | Phase II Clinical Trial |
| *PDE4B* | Asthma | Approved |
| *FGFR1* | Atherosclerosis | Phase II Clinical Trial |
| *P2RY12* | Atherosclerosis | Approved |
| *ESR1* | Atrophic Vaginitis | Approved |
| *DRD2* | Attention Deficit Disorder with Hyperactivity | Phase III Clinical Trial |
| *DRD2* | Autistic Disorder | Approved |
| *FGFR1* | Biliary Tract Neoplasms | Phase III Clinical Trial |
| *ERBB4* | Biliary Tract Neoplasms | Phase III Clinical Trial |
| *ESR1* | Bipolar Disorder | Phase II Clinical Trial |
| *DRD2* | Bipolar Disorder | Approved |
| *DRD2* | Brain Injuries, Traumatic | Phase II Clinical Trial |
| *FGFR1* | Brain Ischemia | Phase I Clinical Trial |
| *P2RY12* | Brain Ischemia | Approved |
| *PDE4B* | Brain Ischemia | Approved |
| *FGFR1* | Brain Neoplasms | Phase II Clinical Trial |
| *ESR1* | Brain Neoplasms | Phase II Clinical Trial |
| *DRD2* | Brain Neoplasms | Phase II Clinical Trial |
| *ERBB4* | Brain Neoplasms | Phase II Clinical Trial |
| *FGFR1* | Breast Neoplasms | Phase II Clinical Trial |
| *ESR1* | Breast Neoplasms | Approved |
| *EGF* | Breast Neoplasms | Phase I Clinical Trial |
| *DRD2* | Breast Neoplasms | Phase II Clinical Trial |
| *TNKS* | Breast Neoplasms | Phase II Clinical Trial |
| *ERBB4* | Breast Neoplasms | Approved |
| *IL1RAP* | Breast Neoplasms | Phase II Clinical Trial |
| *FGFR1* | Burns | Approved |
| *FGFR1* | Carcinoid Tumor | Phase I Clinical Trial |
| *FGFR1* | Carcinoma, Non-Small-Cell Lung | Phase III Clinical Trial |
| *EGF* | Carcinoma, Non-Small-Cell Lung | Approved |
| *DRD2* | Carcinoma, Non-Small-Cell Lung | Phase II Clinical Trial |
| *ERBB4* | Carcinoma, Non-Small-Cell Lung | Phase III Clinical Trial |
| *IL1RAP* | Carcinoma, Non-Small-Cell Lung | Phase II Clinical Trial |
| *PREP* | Celiac Disease | Phase II Clinical Trial |
| *MAPT* | Central Nervous System Diseases | Phase III Clinical Trial |
| *DRD2* | Central Nervous System Neoplasms | Approved |
| *P2RY12* | Cerebral Infarction | Approved |
| *PDE4B* | Cerebral Infarction | Approved |
| *DRD2* | Cocaine-Related Disorders | Phase II Clinical Trial |
| *GRM5* | Cocaine-Related Disorders | Phase II Clinical Trial |
| *PDE4B* | Colitis, Ulcerative | Phase III Clinical Trial |
| *FGFR1* | Colorectal Neoplasms | Phase III Clinical Trial |
| *DRD2* | Colorectal Neoplasms | Phase II Clinical Trial |
| *ERBB4* | Colorectal Neoplasms | Phase II Clinical Trial |
| *IL1RAP* | Colorectal Neoplasms | Phase II Clinical Trial |
| *DRD2* | Conduct Disorder | Phase III Clinical Trial |
| *PDE4B* | Conjunctivitis, Allergic | Approved |
| *ESR1* | Constriction, Pathologic | Phase II Clinical Trial |
| *PDE4B* | Constriction, Pathologic | Approved |
| *PDE4B* | Crohn Disease | Phase III Clinical Trial |
| *PDE4B* | Cystic Fibrosis | Phase II Clinical Trial |
| *DRD2* | Dementia | Approved |
| *DRD2* | Dementia, Vascular | Approved |
| *CHAT* | Depression | Phase III Clinical Trial |
| *DRD2* | Depression | Approved |
| *GRM5* | Depression | Phase I Clinical Trial |
| *PDE4B* | Depression | Phase I Clinical Trial |
| *GRIN2B* | Depression | Phase I Clinical Trial |
| *DRD2* | Depressive Disorder, Major | Approved |
| *GRM5* | Depressive Disorder, Major | Phase II Clinical Trial |
| *GRIN2B* | Depressive Disorder, Major | Phase II Clinical Trial |
| *PDE4B* | Dermatitis, Atopic | Approved |
| *ESR1* | Dermatitis, Seborrheic | Approved |
| *FGFR1* | Diabetes Complications | Approved |
| *DRD2* | Diabetes Complications | Approved |
| *MTTP* | Diabetes Mellitus, Type 2 | Phase II Clinical Trial |
| *DRD2* | Diabetes Mellitus, Type 2 | Approved |
| *VEGFB* | Diabetes Mellitus, Type 2 | Phase I Clinical Trial |
| *PDE4B* | Diabetes Mellitus, Type 2 | Phase I Clinical Trial |
| *VEGFB* | Diabetic Nephropathies | Phase I Clinical Trial |
| *PDE4B* | Diabetic Nephropathies | Phase I Clinical Trial |
| *GRIN2B* | Diabetic Neuropathies | Phase II Clinical Trial |
| *DRD2* | Disease | Phase I Clinical Trial |
| *PDE4B* | Disease | Phase I Clinical Trial |
| *DRD2* | Dyskinesia, Drug-Induced | Phase I Clinical Trial |
| *GRM5* | Dyskinesia, Drug-Induced | Phase II Clinical Trial |
| *DRD2* | Dyskinesias | Approved |
| *ESR1* | Dysmenorrhea | Approved |
| *DRD2* | Dysmenorrhea | Approved |
| *ESR1* | Dyspareunia | Approved |
| *DRD2* | Dyspepsia | Approved |
| *GRM5* | Dystonia | Phase I Clinical Trial |
| *FGFR1* | Endometrial Neoplasms | Phase II Clinical Trial |
| *ESR1* | Endometrial Neoplasms | Phase II Clinical Trial |
| *DRD2* | Endometrial Neoplasms | Phase II Clinical Trial |
| *ERBB4* | Endometrial Neoplasms | Phase II Clinical Trial |
| *ESR1* | Endometriosis | Approved |
| *GRIN2B* | Epilepsy | Phase II Clinical Trial |
| *DRD2* | Erectile Dysfunction | Approved |
| *FGFR1* | Esophageal Neoplasms | Phase II Clinical Trial |
| *ERBB4* | Esophageal Neoplasms | Phase II Clinical Trial |
| *CHAT* | Essential Tremor | Phase II Clinical Trial |
| *FGFR1* | Fallopian Tube Neoplasms | Phase III Clinical Trial |
| *DRD2* | Fibromyalgia | Phase II Clinical Trial |
| *FGFR1* | Fractures, Bone | Phase II Clinical Trial |
| *GRM5* | Fragile X Syndrome | Phase II Clinical Trial |
| *MAPT* | Frontotemporal Dementia | Phase III Clinical Trial |
| *DRD2* | Galactorrhea | Approved |
| *DRD2* | Gastritis | Approved |
| *DRD2* | Gastroesophageal Reflux | Approved |
| *GRM5* | Gastroesophageal Reflux | Phase II Clinical Trial |
| *DRD2* | Gastrointestinal Diseases | Approved |
| *FGFR1* | Gastrointestinal Neoplasms | Phase II Clinical Trial |
| *ERBB4* | Gastrointestinal Neoplasms | Phase II Clinical Trial |
| *DRD2* | Gastroparesis | Approved |
| *ESR1* | Glaucoma | Phase I Clinical Trial |
| *ESR1* | Gynecomastia | Phase II Clinical Trial |
| *FGFR1* | Head and Neck Neoplasms | Phase II Clinical Trial |
| *ERBB4* | Head and Neck Neoplasms | Phase II Clinical Trial |
| *DRD2* | Headache | Approved |
| *DRD2* | Heart Failure | Phase III Clinical Trial |
| *PDE4B* | Heart Failure | Approved |
| *FGFR1* | Hematologic Neoplasms | Phase II Clinical Trial |
| *ESR1* | Hirsutism | Approved |
| *DRD2* | Huntington Disease | Phase III Clinical Trial |
| *GRM5* | Huntington Disease | Phase II Clinical Trial |
| *PDE4B* | Huntington Disease | Phase I Clinical Trial |
| *GRIN2B* | Huntington Disease | Phase III Clinical Trial |
| *MTTP* | Hypercholesterolemia | Approved |
| *MTTP* | Hyperlipidemias | Phase II Clinical Trial |
| *MTTP* | Hyperlipoproteinemia Type II | Approved |
| *DRD2* | Hyperprolactinemia | Approved |
| *DRD2* | Hypertension | Phase II Clinical Trial |
| *DRD2* | Hypertension, Pulmonary | Phase II Clinical Trial |
| *MTTP* | Hypertriglyceridemia | Phase II Clinical Trial |
| *PDE4B* | Hypertriglyceridemia | Phase II Clinical Trial |
| *ESR1* | Hypogonadism | Phase III Clinical Trial |
| *ALPL* | Hypophosphatasia | Approved |
| *PDE4B* | Idiopathic Pulmonary Fibrosis | Phase II Clinical Trial |
| *PDE4B* | Immune System Diseases | Phase I Clinical Trial |
| *ESR1* | Infertility, Female | Approved |
| *LIFR* | Infertility, Female | Phase II Clinical Trial |
| *DRD2* | Infertility, Female | Approved |
| *PDE4B* | Inflammation | Approved |
| *ESR1* | Inflammatory Bowel Diseases | Phase I Clinical Trial |
| *PDE4B* | Inflammatory Bowel Diseases | Phase III Clinical Trial |
| *CHAT* | Influenza, Human | Phase II Clinical Trial |
| *FGFR1* | Intermittent Claudication | Phase I Clinical Trial |
| *P2RY12* | Intracranial Thrombosis | Approved |
| *FGFR1* | Kidney Neoplasms | Approved |
| *FGFR1* | Leiomyosarcoma | Phase III Clinical Trial |
| *FGFR1* | Leukemia, Lymphocytic, Chronic, B-Cell | Phase I Clinical Trial |
| *FGFR1* | Leukemia, Myelogenous, Chronic, BCR-ABL Positive | Approved |
| *FGFR1* | Leukemia, Myeloid, Acute | Phase I Clinical Trial |
| *PTPRC* | Leukemia, Myeloid, Acute | Phase III Clinical Trial |
| *DRD2* | Leukemia, Myeloid, Acute | Phase II Clinical Trial |
| *FGFR1* | Liposarcoma | Phase I Clinical Trial |
| *FGFR1* | Liver Neoplasms | Approved |
| *ERBB4* | Liver Neoplasms | Phase I Clinical Trial |
| *FGFR1* | Lung Neoplasms | Phase I Clinical Trial |
| *TNKS* | Lung Neoplasms | Phase II Clinical Trial |
| *ERBB4* | Lung Neoplasms | Phase I Clinical Trial |
| *PDE4B* | Lupus Erythematosus, Cutaneous | Phase II Clinical Trial |
| *ESR1* | Lupus Erythematosus, Systemic | Phase I Clinical Trial |
| *FGFR1* | Lymphoma | Phase I Clinical Trial |
| *EIF4E* | Lymphoma | Phase I Clinical Trial |
| *TNKS* | Lymphoma, B-Cell | Phase II Clinical Trial |
| *DRD2* | Lymphoma, Non-Hodgkin | Phase II Clinical Trial |
| *FGFR1* | Melanoma | Phase II Clinical Trial |
| *TNKS* | Melanoma | Phase II Clinical Trial |
| *ERBB4* | Melanoma | Phase I Clinical Trial |
| *ESR1* | Menorrhagia | Approved |
| *FGFR1* | Mesothelioma | Phase I Clinical Trial |
| *ESR1* | Migraine Disorders | Phase II Clinical Trial |
| *DRD2* | Migraine Disorders | Approved |
| *GRM5* | Migraine Disorders | Phase I Clinical Trial |
| *FGFR1* | Multiple Myeloma | Phase II Clinical Trial |
| *DRD2* | Multiple Myeloma | Phase II Clinical Trial |
| *PDE4B* | Multiple Sclerosis | Phase II Clinical Trial |
| *PDE4B* | Multiple Sclerosis, Chronic Progressive | Phase II Clinical Trial |
| *ESR1* | Multiple Sclerosis, Relapsing-Remitting | Phase II Clinical Trial |
| *PDE4B* | Multiple Sclerosis, Relapsing-Remitting | Phase II Clinical Trial |
| *DRD2* | Myelodysplastic Syndromes | Phase II Clinical Trial |
| *PDE4B* | Myelodysplastic Syndromes | Phase II Clinical Trial |
| *ESR1* | Myocardial Infarction | Phase III Clinical Trial |
| *P2RY12* | Myocardial Infarction | Approved |
| *PDE4B* | Myocardial Infarction | Approved |
| *ERBB4* | Nasopharyngeal Neoplasms | Phase I Clinical Trial |
| *NTN1* | Neoplasms | Phase I Clinical Trial |
| *FGFR1* | Neoplasms | Phase III Clinical Trial |
| *ESR1* | Neoplasms | Phase II Clinical Trial |
| *EGF* | Neoplasms | Phase I Clinical Trial |
| *MFGE8* | Neoplasms | Phase I Clinical Trial |
| *DRD2* | Neoplasms | Phase I Clinical Trial |
| *EIF4E* | Neoplasms | Phase I Clinical Trial |
| *METAP1* | Neoplasms | Phase I Clinical Trial |
| *TNKS* | Neoplasms | Phase II Clinical Trial |
| *ERBB4* | Neoplasms | Phase II Clinical Trial |
| *ERBB4* | Neoplasms, Squamous Cell | Phase I Clinical Trial |
| *DRD2* | Nervous System Diseases | Approved |
| *GRIN2B* | Nervous System Diseases | Phase II Clinical Trial |
| *PREP* | Neurocognitive Disorders | Phase II Clinical Trial |
| *ESR1* | Neurocognitive Disorders | Phase II Clinical Trial |
| *PDE4B* | Neurocognitive Disorders | Phase II Clinical Trial |
| *FGFR1* | Neuroectodermal Tumors | Phase II Clinical Trial |
| *FGFR1* | Neuroendocrine Tumors | Phase III Clinical Trial |
| *DRD2* | Nociceptive Pain | Approved |
| *PDE4B* | Nociceptive Pain | Approved |
| *PDE4B* | Non-alcoholic Fatty Liver Disease | Phase II Clinical Trial |
| *DRD2* | Nutritional and Metabolic Diseases | Approved |
| *FGFR1* | Obesity | Phase I Clinical Trial |
| *MTTP* | Obesity | Phase II Clinical Trial |
| *DRD2* | Obesity | Phase II Clinical Trial |
| *DRD2* | Obsessive-Compulsive Disorder | Phase III Clinical Trial |
| *GRM5* | Obsessive-Compulsive Disorder | Phase II Clinical Trial |
| *SLC6A9* | Obsessive-Compulsive Disorder | Phase II Clinical Trial |
| *PDE4B* | Opioid-Related Disorders | Phase II Clinical Trial |
| *PDE4B* | Osteoarthritis | Phase II Clinical Trial |
| *ESR1* | Osteoporosis | Approved |
| *FGFR1* | Osteosarcoma | Phase II Clinical Trial |
| *FGFR1* | Ovarian Neoplasms | Phase III Clinical Trial |
| *DRD2* | Ovarian Neoplasms | Phase II Clinical Trial |
| *TNKS* | Ovarian Neoplasms | Phase II Clinical Trial |
| *ERBB4* | Ovarian Neoplasms | Phase II Clinical Trial |
| *FGFR1* | Pancreatic Neoplasms | Phase II Clinical Trial |
| *TNKS* | Pancreatic Neoplasms | Phase II Clinical Trial |
| *ERBB4* | Pancreatic Neoplasms | Phase II Clinical Trial |
| *IL1RAP* | Pancreatic Neoplasms | Phase II Clinical Trial |
| *DRD2* | Panic Disorder | Phase I Clinical Trial |
| *SLC6A9* | Panic Disorder | Phase II Clinical Trial |
| *DRD2* | Parkinson Disease | Approved |
| *GRM5* | Parkinson Disease | Phase II Clinical Trial |
| *PDE4B* | Parkinson Disease | Phase I Clinical Trial |
| *MAPT* | Parkinson Disease | Phase I Clinical Trial |
| *GRIN2B* | Parkinson Disease | Phase I Clinical Trial |
| *FGFR1* | Peripheral Vascular Diseases | Phase I Clinical Trial |
| *DRD2* | Peripheral Vascular Diseases | Phase II Clinical Trial |
| *P2RY12* | Peripheral Vascular Diseases | Approved |
| *FGFR1* | Peritoneal Neoplasms | Phase III Clinical Trial |
| *ESR1* | Peritoneal Neoplasms | Phase II Clinical Trial |
| *DRD2* | Phobia, Social | Phase III Clinical Trial |
| *ESR1* | Pituitary ACTH Hypersecretion | Approved |
| *ESR1* | Polycystic Ovary Syndrome | Approved |
| *DRD2* | Postoperative Nausea and Vomiting | Approved |
| *FGFR1* | Precursor Cell Lymphoblastic Leukemia-Lymphoma | Approved |
| *DRD2* | Precursor Cell Lymphoblastic Leukemia-Lymphoma | Phase II Clinical Trial |
| *EIF4E* | Precursor Cell Lymphoblastic Leukemia-Lymphoma | Phase I Clinical Trial |
| *ESR1* | Premenstrual Syndrome | Approved |
| *FGFR1* | Pressure Ulcer | Approved |
| *FGFR1* | Prostatic Neoplasms | Phase II Clinical Trial |
| *ESR1* | Prostatic Neoplasms | Approved |
| *EGF* | Prostatic Neoplasms | Phase II Clinical Trial |
| *DRD2* | Prostatic Neoplasms | Phase II Clinical Trial |
| *EIF4E* | Prostatic Neoplasms | Phase II Clinical Trial |
| *ERBB4* | Prostatic Neoplasms | Phase I Clinical Trial |
| *PDE4B* | Pruritus | Phase I Clinical Trial |
| *PDE4B* | Psoriasis | Approved |
| *DRD2* | Psychotic Disorders | Approved |
| *PDE4B* | Pulmonary Disease, Chronic Obstructive | Approved |
| *PDE4B* | Reperfusion Injury | Phase II Clinical Trial |
| *ESR1* | Reproductive Tract Infections | Approved |
| *DRD2* | Restless Legs Syndrome | Approved |
| *DRD2* | Rett Syndrome | Phase III Clinical Trial |
| *FGFR1* | Rhabdomyosarcoma | Phase II Clinical Trial |
| *PDE4B* | Rhinitis, Allergic, Seasonal | Phase II Clinical Trial |
| *PDE4B* | Sarcoidosis | Phase II Clinical Trial |
| *FGFR1* | Sarcoma | Approved |
| *FGFR1* | Sarcoma, Ewing | Phase II Clinical Trial |
| *FLI1* | Sarcoma, Ewing | Phase I Clinical Trial |
| *FGFR1* | Sarcoma, Synovial | Phase III Clinical Trial |
| *DRD2* | Schizophrenia | Approved |
| *GRM5* | Schizophrenia | Phase I Clinical Trial |
| *PDE4B* | Schizophrenia | Phase I Clinical Trial |
| *SLC6A9* | Schizophrenia | Phase III Clinical Trial |
| *ESR1* | Sjogren's Syndrome | Phase II Clinical Trial |
| *ERBB4* | Skin Neoplasms | Phase II Clinical Trial |
| *DRD2* | Sleep Initiation and Maintenance Disorders | Phase III Clinical Trial |
| *MFGE8* | Small Cell Lung Carcinoma | Phase II Clinical Trial |
| *GRM7* | Spasms, Infantile | Phase I Clinical Trial |
| *FGFR1* | Spinal Cord Injuries | Phase II Clinical Trial |
| *PDE4B* | Spondylitis, Ankylosing | Phase III Clinical Trial |
| *GRM7* | Status Epilepticus | Phase I Clinical Trial |
| *FGFR1* | Stomach Neoplasms | Phase II Clinical Trial |
| *ERBB4* | Stomach Neoplasms | Phase III Clinical Trial |
| *DRD2* | Stress Disorders, Post-Traumatic | Phase III Clinical Trial |
| *DRD2* | Substance-Related Disorders | Approved |
| *MAPT* | Supranuclear Palsy, Progressive | Phase II Clinical Trial |
| *FGFR1* | Telangiectasia, Hereditary Hemorrhagic | Phase II Clinical Trial |
| *FGFR1* | Testicular Neoplasms | Phase II Clinical Trial |
| *SLC6A9* | Thalassemia | Phase II Clinical Trial |
| *P2RY12* | Thrombosis | Approved |
| *PDE4B* | Thrombosis | Approved |
| *FGFR1* | Thyroid Neoplasms | Approved |
| *DRD2* | Tourette Syndrome | Approved |
| *ESR1* | Turner Syndrome | Phase III Clinical Trial |
| *FGFR1* | Ulcer | Phase III Clinical Trial |
| *FGFR1* | Urethral Neoplasms | Phase II Clinical Trial |
| *FGFR1* | Urinary Bladder Neoplasms | Phase II Clinical Trial |
| *ERBB4* | Urinary Bladder Neoplasms | Phase II Clinical Trial |
| *FGFR1* | Uterine Cervical Neoplasms | Phase II Clinical Trial |
| *ERBB4* | Uterine Cervical Neoplasms | Phase II Clinical Trial |
| *ESR1* | Vaginitis | Approved |
| *ALPL* | Vascular Calcification | Phase I Clinical Trial |
| *DRD2* | Vomiting | Approved |
| *FGFR1* | Wounds and Injuries | Approved |
| *ESR1* | Wounds and Injuries | Phase III Clinical Trial |
