# Supplementary figures and images for: Gene-based polygenic risk scores analysis of alcohol use disorder in African Americans
Source: Transl Psychiatry. 2022 Jul 5;12:266. doi: 10.1038/s41398-022-02029-2 (PMC9256707; doi:10.1038/s41398-022-02029-2)

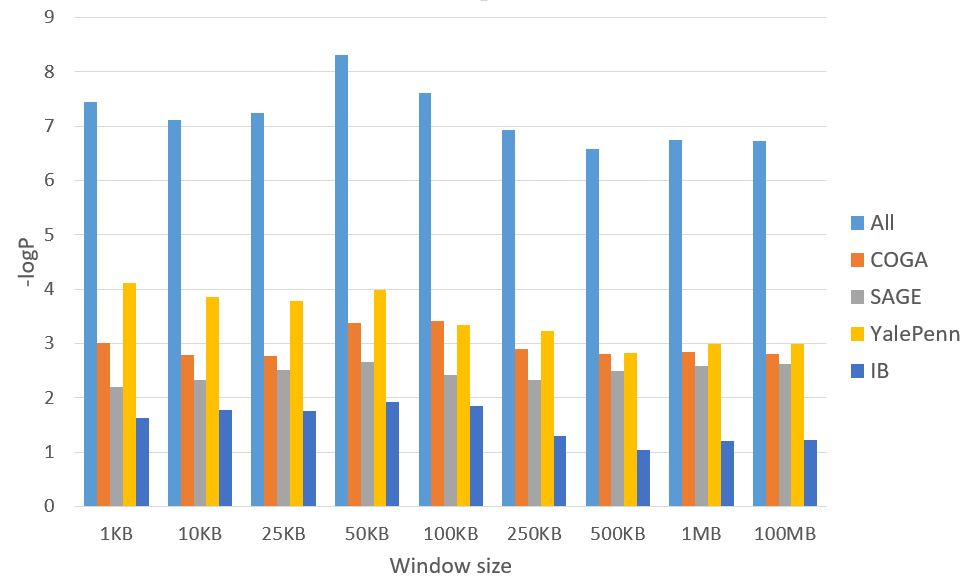

Supplement: Supplementary file 7 — Negative logP-values of PRSgene using different window sizes to extend gene boundaries. [file 41398_2022_2029_MOESM7_ESM.jpg]

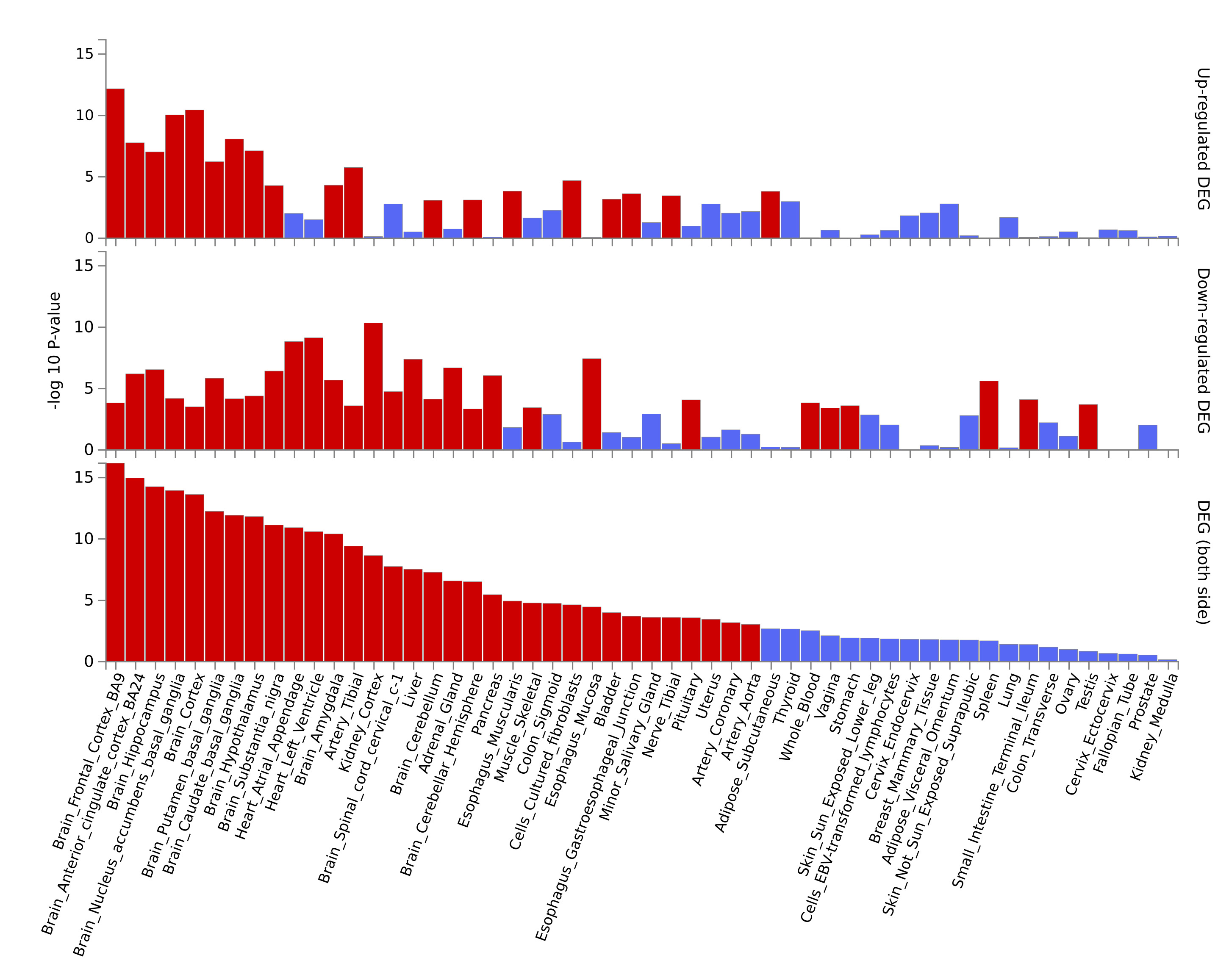

Supplement: Supplementary file 8 — Tissue-specific Differentially Expressed Gene enrichment analysis. [file 41398_2022_2029_MOESM8_ESM.jpg]
